# Efflux pump inhibitory potential of indole derivatives as an arsenal against *norA* over-expressing *Staphylococcus aureus*

**DOI:** 10.1128/spectrum.04876-22

**Published:** 2023-09-27

**Authors:** Nishtha Chandal, Rushikesh Tambat, Ritu Kalia, Gautam Kumar, Nisha Mahey, Sanjay Jachak, Hemraj Nandanwar

**Affiliations:** 1 Clinical Microbiology and Antimicrobial Research Laboratory, CSIR-Institute of Microbial Technology, Chandigarh, India; 2 Academy of Scientific & Innovative Research (AcSIR), Ghaziabad, Uttar Pradesh, India; 3 Department of Natural Products, National Institute of Pharmaceutical Education and Research, Mohali, Punjab, India; 4 Department of Natural Products, Chemical Sciences, National Institute of Pharmaceutical Education and Research- Hyderabad, Balanagar, Telangana, India; University of North Carolina at Chapel Hill, Chapel Hill, North Carolina, USA

**Keywords:** *Staphylococcus aureus*, major facilitator superfamily (MFS), *norA* efflux pump, efflux pump inhibitors (EPIs), multidrug resistance (MDR)

## Abstract

NorA, an extensively studied efflux pump in *Staphylococcus aureus*, has been connected to fluoroquinolone, antiseptic, and disinfection resistance. Several studies have also emphasized how efflux pumps, including NorA, function as the first line of defense of *S. aureus* against antibiotics. In this study, we have screened some chemically synthesized indole derivatives for their activity as efflux pump inhibitors (EPIs). The derivative SMJ-5 was found to be a potent NorA efflux pump inhibitor among the screened indole derivatives, owing to increased ethidium bromide and norfloxacin accumulation in *norA* over-expressing *S. aureus*. The combination of SMJ-5 and ciprofloxacin demonstrated the eradication of *S. aureus* biofilm and prolonged the post-antibiotic effect more than ciprofloxacin alone. SMJ-5 was able to inhibit staphyloxanthin virulence. In *in vitro* time-kill trials and *in vivo* efficacy investigations, the combination enhanced the bactericidal activity of ciprofloxacin against *S. aureus*. Additionally, reverse transcription PCR results revealed that SMJ-5 also inhibits the NorA efflux pump indirectly at the transcriptional level.

**IMPORTANCE:**

The NorA efflux pump is the most effective resistance mechanism in *S. aureus*. The clinical importance of NorA efflux pumps is demonstrated by the expression of pump genes in *S. aureus* strains in response to fluoroquinolones and biocides. Along with the repercussions of decreased fluoroquinolone sensitivity, increasing expression of efflux pump genes by their substrate necessitates the importance of efflux pump inhibitors. Reserpine and verapamil are clinically used to treat ailments and have proven NorA inhibitors, but, unfortunately, the concentration needed for these drugs to inhibit the pump is not safe in clinical settings. In the current study, we have screened some indole derivatives, and among them, SMJ-5 was reported to potentiate norfloxacin and ciprofloxacin at their sub-inhibitory concentration by inhibiting the *norA* gene transcriptionally. Here we highlight the promising points of this study, which could serve as a model to design a therapeutic EPI candidate against *norA* over-expressing *S. aureus*.

## INTRODUCTION

Multidrug resistance (MDR) is one of the primary causes of death worldwide and continues to be a severe challenge in treating infectious diseases (1). The advent and fast spread of multidrug-resistant bacteria pose a serious threat to global healthcare. *S. aureus*, an opportunistic pathogen, can cause many complications with mild to life-threatening outcomes. *S. aureus* can also form biofilms, leading to higher antibiotic tolerance (2). Fluoroquinolones have a broad spectrum of activity against a range of bacterial species, including methicillin-resistant *Staphylococcus aureus* (MRSA) (3). However, the clinical relevance of these antibiotics was somewhat debilitated due to their abrupt evolution in resistance (4). Around 25% of *S. aureus* isolates in Europe are resistant to fluoroquinolones, which comprise about 90% of MRSA isolates (5). Also, studies have proved that resistance against fluoroquinolones is related to a mutation in GrlA and GrlB proteins of topoisomerase IV and GyrA and GyrB proteins of DNA gyrase (6).

Among the various factors that cause resistance, one of the significant factors is the efflux pumps, which transport structurally distinct, noxious compounds such as antibiotics outside the cell; therefore, these efflux pumps reduce their intracellular concentration and lead to multidrug resistance (7, 8). The chromosomally encoded NorA is a 388-amino acid efflux pump with 12 transmembrane helices and a molecular weight of 42.2 kDa (9). The NorA efflux pump has a wide range of substrates, including fluoroquinolones, dyes, antiseptics, and quaternary ammonium compounds (10). NorA efflux pump uses proton-motive force to expel these compounds out of the cell.

Moreover, efflux pumps have been linked to virulence and biofilm formation. Efflux pumps enhance biofilm formation by excreting toxic compounds and signaling molecules while influencing surface adhesion (10, 11). Efflux pump inhibitors (EPIs) have been observed to potentiate the existing antibiotics, overcoming the resistance in bacteria (10). *S. aureus* strains were unable to form biofilms, and the expression of the *norA* efflux pump genes was reduced by toluidine blue O-mediated photodynamic treatment (12). Nilotinib was reported as a NorA efflux pump inhibitor. It significantly reduced the growth of *S. aureus* biofilms, indicating that this substance interacted with the NorA efflux pump and decreased activity (10). The anti-fungal ketoconazole inhibits the NorA efflux pump and biofilm development in *S. aureus* (13). Considering the role of efflux pumps in antibiotic resistance, biofilm formation, and virulence, we found that there have been various efforts to discover and develop efflux pump inhibitors. Still, none has been approved due to their toxicity. With the barren antibiotic pipeline in today’s era, there is a dire need to adopt new strategies to revitalize existing antibiotics using antibiotic potentiators.

This study observed the efflux pump inhibitory effect and the ciprofloxacin potentiation efficacy of indole derivatives in *S. aureus* by inhibiting the NorA efflux pump. Our findings shed light on the efflux pump inhibitory effect of indole derivatives, which helps to reduce the pre-formed *S. aureus* biofilm. This study also demonstrates the efficacy of the most potent efflux pump inhibitor, SMJ-5, in *in vivo* murine infection models.

## RESULTS

### Effect of indole-based derivatives on the MIC of antibiotics and its structure-activity relationship

We have synthesized eight derivatives of the indole scaffold (Table S1). The ^1^H NMR and ^13^C NMR spectra of 2-(2′-aminophenyl) indole [RP2] (Fig. S1) and mass spectra, ^1^H NMR, and ^13^C NMR spectra of its indole derivatives are mentioned in the supplemental data (Fig. S2). The antibiotic boosting activity of indole-based derivatives was quantified by observing the reduction in minimum inhibitory concentrations (MICs) of antibiotics. The MIC value of the antibiotics ciprofloxacin and norfloxacin was reduced when coupled with indole-based derivatives at sub-inhibitory concentrations (1/4×, 1/8×, and 1/16× MIC), where no intrinsic anti-bacterial activity was observed. SMJ-3, SMJ-5, SMJ-9, and SMJ-10 demonstrated a momentous fall in ciprofloxacin and norfloxacin MICs with a fold change of 32, 16, 32, 16, and 16, 32, 8, and 16, respectively, in *S. aureus* SA-1199B (*norA*-over-expressed strain). The fractional inhibitory concentration index (FICI) value for SMJ-3, SMJ-5, SMJ-9, and SMJ-10 was ≤0.5, indicating synergy with ciprofloxacin and norfloxacin (Table 1). However, a minor or no change in modulation was observed in the MIC value for *S. aureus* K-1758 (*norA* deletion strain), and a FICI value of >0.5 to <4.0 was observed, which indicates indifference (Table S2). Hence, synergy observed with *S. aureus* SA-1199B *norA* over-expressing strain may be due to indole derivatives behaving as NorA efflux pump inhibitors.

**TABLE 1:** Checkerboard synergy assay of indole derivatives against *S. aureus* SA-1199B (*norA*-over-expressed strain)^*a*^

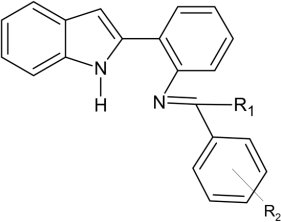

Compound	R_1_	R_2_	Intrinsic MIC (µg/mL)	Concentration	Norfloxacin			Ciprofloxacin		
					MIC (µg/mL)	Modulation factor	FICI	MIC (µg/mL)	Modulation Factor	FICI
SMJ-1	CH_3_	−CH_3_	2	0.5	16	4	0.5	2	4	0.50
				0.25	32	2	0.625	4	2	0.625
				0.125	32	2	0.562	4	2	0.562
				0	64	–	1	8	–	1
SMJ-3	CH_3_	−OH	16	4	4	16	0.312	0.25	32	0.281
				2	8	8	0.25	0.5	16	0.187
				1	16	4	0.312	2	4	0.312
				0	64	–	1	8	–	1
SMJ-5	CH_3_	−Dihydroxyl	128	32	2	32	0.281	0.5	16	0.312
				16	4	16	0.187	1	8	0.25
				8	16	4	0.312	4	2	0.562
				0	64	–	1	8	–	1
SMJ-6	H	−F	4	1	16	4	0.5	4	2	0.75
				0.5	32	2	0.625	4	2	0.625
				0.25	64	–	1.062	8	–	1.062
				0	64	–	1	8	-	1
SMJ-7	CH_3_	−NO_2_	128	32	8	8	0.375	1	8	0.375
				16	16	4	0.375	2	4	0.375
				8	32	2	0.562	8	–	1.062
				0	64	–	1	8	–	1
SMJ-8	H	−CF_3_	64	16	64	–	1.25	8	–	1.25
				8	64	–	1.125	8	–	1.125
				4	64	–	1.062	8	–	1.062
				0	64	–	1	8	–	1
SMJ-9	CH_3_	−SCH_3_	16	4	8	8	0.375	0.25	32	0.281
				2	16	4	0.375	0.5	16	0.187
				1	16	4	0.312	0.5	16	0.125
				0	64	–	1	8	–	1
SMJ-10	CH_3_	NH_2_	128	32	4	16	0.312	0.5	16	0.312
				16	8	8	0.25	2	4	0.372
				8	32	2	0.562	4	2	0.562
				0	64	–	1	8	–	1
Reserpine			128	32	8	8	0.381	0.5	16	0.318
				16	16	4	0.378	1	8	0.253
				8	8	8	0.314	2	4	0.314
				0	64	–	1	8	–	1

^*a*^ Modulation factor = fold change in MIC of antibiotic in the presence of the indole derivatives.

Also, the indole derivatives, when coupled with erythromycin in a strain expressing MsrA (*S. aureus* RN-4220), showed no synergism but was indicative of indifference as the FICI observed was >0.5 and <4.0 (Table S3). The indole derivatives were also coupled with NorB substrate moxifloxacin (14), and a checkerboard synergy assay was performed with the over-expressing NorA strain (*S. aureus* SA-1199B). Since moxifloxacin is not the substrate of NorA efflux pumps, no synergy was observed (Table S4a) (15). The development of combination therapy will be greatly aided by the use of currently prescribed drugs and NorA inhibitors. Also, the MIC spectrum of fluoroquinolones (ciprofloxacin, norfloxacin, levofloxacin and moxifloxacin) and tetracycline is shown on *S. aureus* SA-1199B (*norA* over-expressing), SA-1199 (*norA* wild-type), and K-1758 (*norA* deletion) strains (Table S4b) to show the susceptibility profile of these strains toward fluoroquinolones. These findings collectively imply that NorA is a target of the derivatives used in the investigation.

According to the structure-activity relationship (SAR), hydrogen, a weak electron-donating group attached to the Schiff’s base (at the R_1_ position), reduces the EPI activity in the case of SMJ-6 and SMJ-8, whereas the derivatives (SMJ-1, SMJ-3, SMJ-5, SMJ-7, SMJ-9, and SMJ-10) having methyl substituent at R_1_ position, being a strong electron-donating group than hydrogen, attached to the Schiff’s base increased the EPI activity. Introducing electron-withdrawing substituents at the *R*_2_ position of the phenyl ring attached to the Schiff base of the indole scaffold resulted in reduced activity (SMJ-6, SMJ-7, and SMJ-8). SMJ-8 containing –CF_3_ at the *R*_2_ position, which is highly electron-withdrawing, loses its activity ultimately compared to –F and –NO_2_ substituents at the *R*_2_ position of SMJ-6 and SMJ-7, respectively, which is weakly active. The SMJ-1-bearing methyl (–CH_3_) group at the *R*_2_ position, being the weakest electron donating group among -OH, -dihydroxyl, -SCH_3_, and NH_2_, present in SMJ-3, SMJ-5, SMJ-9, and SMJ-10, respectively, has a minor activity. However, strong electron-donor groups at the *R*_2_ position increase the EPI activity. Hence, the SAR revealed that strong electron-donating groups at the R_1_ and *R*_2_ positions are the critical feature of bioactivity (SMJ-3, SMJ-5, SMJ-9, and SMJ-10) (Fig. S3).

### Effect of SMJ-5 on clinical strains

To check the efficacy of the combination SMJ-5 (1/4× MIC) and ciprofloxacin in a clinical setting, we studied the synergy of the combination in 17 clinical isolates. Moreover, SMJ-5, along with ciprofloxacin, displayed an activity ranging from 2- to 64-fold modulation. According to previous studies, a decrease in the MIC by at least fourfold compared to their initial concentration in the presence of EPI indicated efflux inhibition (16). Here, we observed a ≥4-fold reduction in 14 strains out of 17, and the combination brings 12 strains out of 17 below the clinical resistance breakpoint, emphasizing the combination can be of high applicability in clinical practice (Table S5) (17, 18). The combination of SMJ-5 and ciprofloxacin exhibits ≥8-fold modulation on five clinical strains. The expression of the *norA* gene in *S. aureus* clinical strains was confirmed through reverse transcription PCR (RT-PCR) analysis, and all the strains showed higher norA expression than the deletion strain, *S. aureus* K-1758. *S. aureus* American Type Culture Collection (ATCC) 29213 was used as a reference sample strain because it has a basal level of NorA efflux pump expression (19). Relative gene expression was determined using the power 2^−ΔΔCT^ technique. The housekeeping gene 16s rRNA was used as an endogenous control to normalize the expression levels of target genes (Fig. S4).

Also, to verify that the result is not due to any other factor than the NorA efflux pump, we assessed the combinatorial action of SMJ-5 with erythromycin and moxifloxacin. Additionally, no discernible decrease in MIC was found with these antibiotics (Table S6).

### Uptake of EtBr and norfloxacin by *S. aureus* SA-1199B cells in the presence of indole derivatives

**FIG 1:**
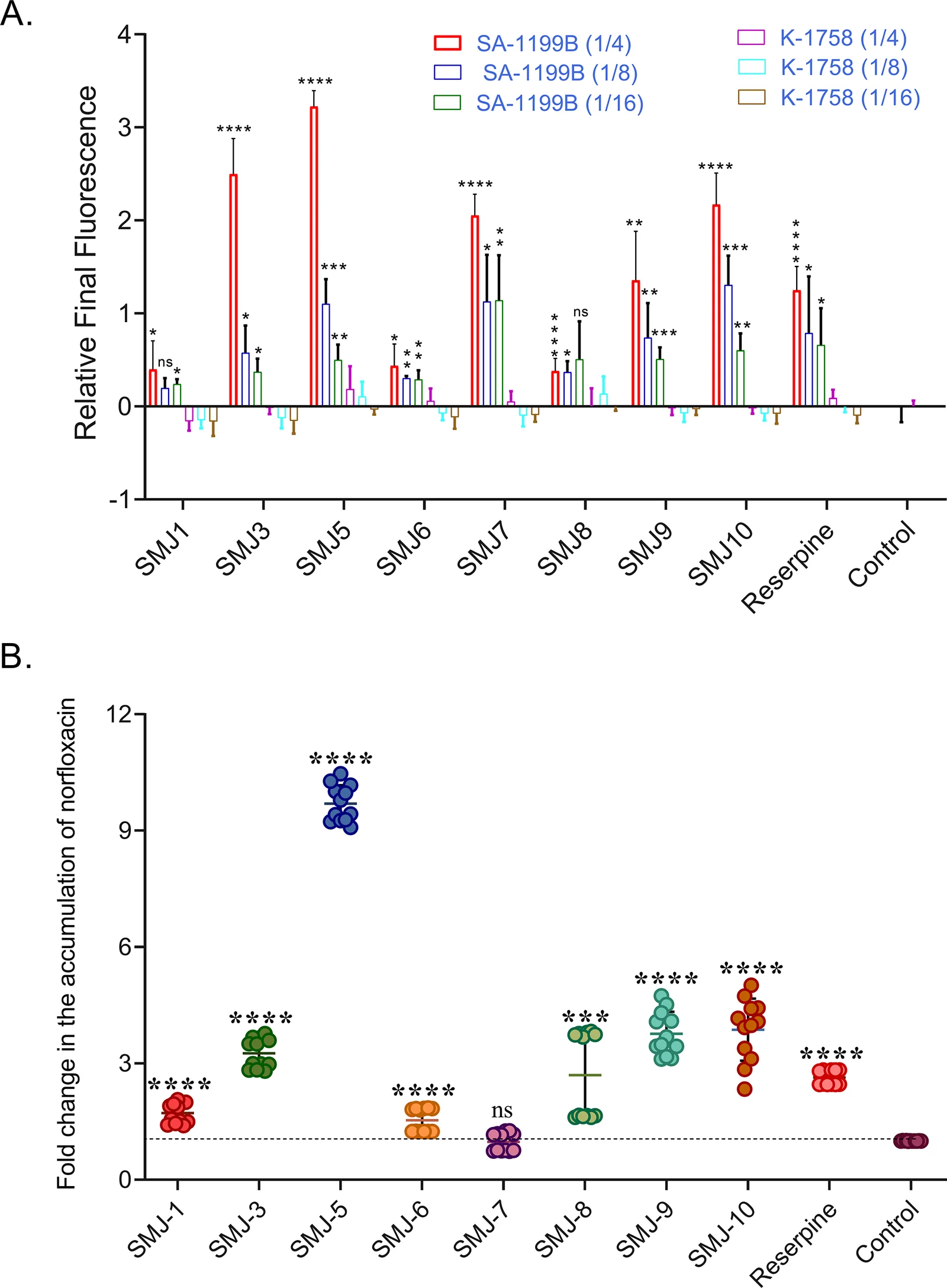
(A) EtBr accumulation assay, RFF value of indole derivatives at 1/4×, 1/8×, and 1/16× MIC in the presence of EtBr on *S. aureus* SA-1199B (*norA* over-expression) and K-1758 (*norA* knockout) strain. (**B**) Norfloxacin accumulation assay on *S. aureus* SA-1199B strain. *P < 0.05, **P < 0.01, ***P < 0.001, ****P < 0.0001. *P* value calculated using 95% class interval. Both experiments were done in biological duplicates, and this is the representation of one, comprising three and six technical replicates, respectively; panel **A** corresponds to average ± SD, and panel **B **is the representation of individual readings ± SD. ns, non-significant; SD, standard deviation.

To corroborate the evidence that indole derivatives inhibit the NorA efflux pump, we further monitored the ethidium bromide (EtBr) and norfloxacin uptake, which are fluorescent NorA efflux pump substrates that bind to the major groove of DNA and DNA gyrase, respectively. Inhibition of the NorA pump by indole derivatives was evident by an increased fluorescence of EtBr at 600 nm in the presence of indole derivatives relative to the fluorescence in the absence of indole derivatives, indicating EtBr accumulation. Among the indole derivatives, SMJ-3 and SMJ-5 displayed significantly enhanced EtBr accumulation, even higher than the positive control reserpine. The relative final fluorescence (RFF) values of SMJ-3 and SMJ-5 at 1/4× MIC were found to be 2.49 ± 0.38 and 3.22 ± 0.17, respectively (Fig. 1A). In contrast, the RFF for positive control reserpine was 1.24 ± 0.25 at 1/4× MIC after 20-min incubation. In the presence of reserpine, EtBr fluorescence was slightly increased in the *S. aureus* K-1758 strain. Still, the negative RFF value for indole derivatives suggested that the treated cells had negligible or no EtBr accumulation than the control cells, suggesting that the NorA efflux pump could be a target for indole derivatives.

The norfloxacin accumulation assay validated the NorA efflux pump inhibition and NorA efflux pump substrate accumulation. Norfloxacin (16 µg/mL) with the sub-inhibitory concentration of indole derivatives demonstrated a substantial increase in norfloxacin accumulation. The fold change in norfloxacin accumulation was lower than that of reserpine for SMJ-1, SMJ-6, and SMJ-7, slightly higher for SMJ-3, SMJ-9, and SMJ-10, and significantly higher for SMJ-5. SMJ-5 accumulated norfloxacin 8.69- and 7.06-folds more elevated than the control (without EPI) and reserpine (positive control), respectively, indicating that it is a potent efflux pump inhibitor (Fig. 1B).

### Rate of extrusion of EtBr in the presence of indole derivatives

**FIG 2:**
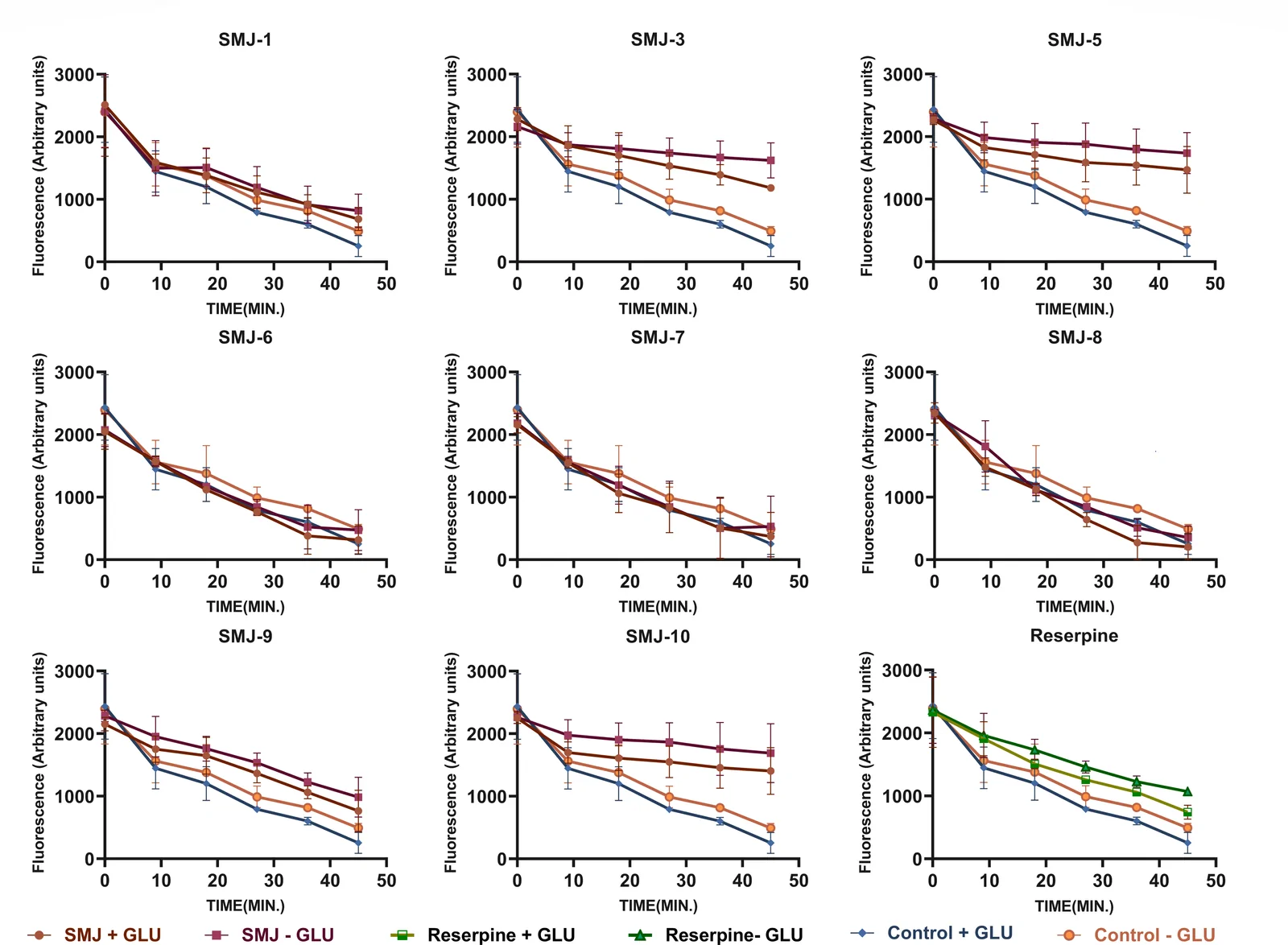
EtBr efflux inhibition assay of indole derivatives at the sub-inhibitory concentration (1/4× MIC) in EtBr presence at 4 µg/mL concentration in the presence and absence of glucose. Reserpine was used as the positive control. The results correspond to the mean of two biological repeats performed in triplicates.

We observed EtBr extrusion over time in *S. aureus* SA-1199B in the presence and absence of indole derivatives. It was found to be significantly reduced in the presence of indole derivatives. These EtBr extrusion results are consistent with the EtBr accumulation assay, and it was determined that SMJ-3, SMJ-5, SMJ-9, and SMJ-10 have a high potential to inhibit efflux pumps. The addition of glucose was observed to lower the efflux pump inhibitory activity of the indole derivatives and reserpine, re-energizing them to promote active efflux (Fig. 2).

### SMJ-5 does not entail membrane depolarization and permeabilization

**FIG 3:**
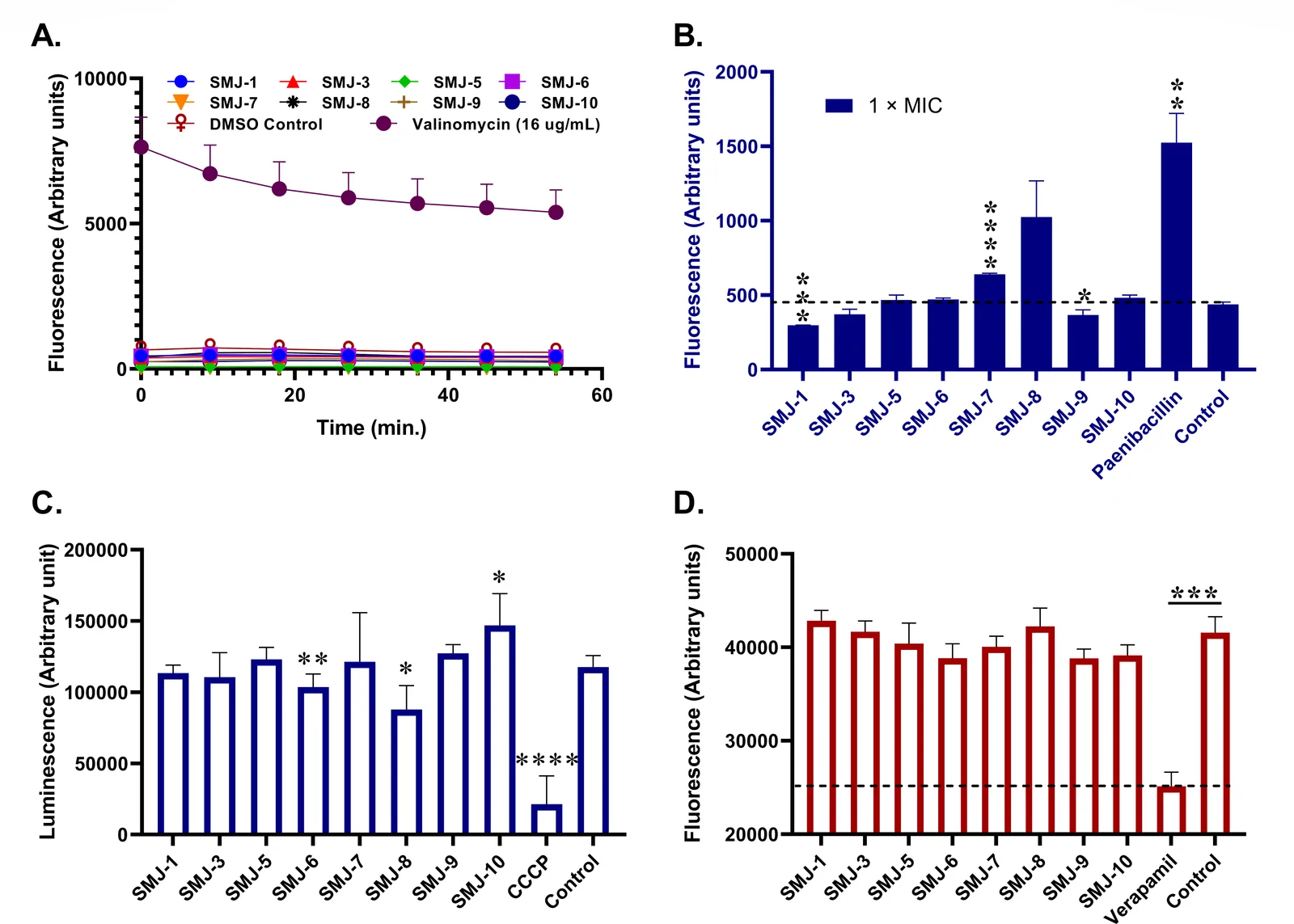
(A) Membrane depolarization was detected using fluorescence of DiSC_3_(5) showing high fluorescence of positive control valinomycin; drug-free control was used as the negative control. (**B**) Membrane permeability was measured using nucleic acid binding dye propidium iodide; paenibacillin was used as the positive control, and drug-free control was used as the negative control. (**C**) ATP luciferin-luciferase Bioluminescence detection assay; CCCP, the positive control, quenches ATP compared to control. (**D**) Mammalian Ca^2+^ channel assay on human embryonic kidney cells. All the experiments represent biological duplicates performed in technical triplicates, and results correspond to average ± SD. *P < 0.05, **P < 0.01, ***P < 0.001, and ****P < 0.0001. *P* value calculated using 95% class interval. CCCP, carbonyl cyanide *m*-chlorophenyl hydrazone.

To track the phenomenon of membrane depolarization, we used the membrane potential-sensitive dye DiSC_3_(5) to quantify changes in the electrical potential gradient in intact bacteria. Valinomycin, used as a positive control, caused massive fluorescence leakage, demonstrating its membrane-depolarizing property (20). In contrast, no such increase in fluorescence was observed in the case of indole derivatives at MIC and 1/2× MIC concentrations (Fig. 3A). The membrane permeabilization was also assessed in the presence of the indole derivatives using propidium iodide (PI). An increase in fluorescence was observed in positive control paenibacillin (21) and SMJ-8. In contrast, no such increase appeared in the case of other indole derivatives, as PI could not enter the viable cell and bind to the target (Fig. 3B). The results confirmed that indole derivatives do not permeabilize the membrane.

### Effect of indole derivatives on the electron transport chain by luciferase luminescence assay

Membrane dysfunction disturbs the respiratory chain functions, resulting in reduced ATP levels. The *S. aureus* SA-1199B isolate was incubated with indole derivatives for 5 h, and the effect of indole derivatives on the intracellular ATP level was evaluated. The ATP levels remained constant compared to the drug-free control, indicating no change in transmembrane potential, ruling out the possibility of ATP depletion as the cause of efflux inhibition. In contrast, a significant reduction in the intracellular ATP was observed in the case of carbonyl cyanide *m*-chlorophenyl hydrazone (CCCP) (positive control), which is a potent mitochondrial uncoupling agent that increases the proton permeability across the mitochondrial inner membranes (22) (Fig. 3C).

### Effect of indole derivatives on mammalian Ca^2+^ channels

We were cognizant that many bacterial efflux inhibitors limit mammalian Ca^2+^ channel activity in clinical settings, such as verapamil, inhibits host Ca^2+^ channels, eliciting human neurotoxicity (23). The mammalian Ca^2+^ channel blocking was observed using the Fluo-4 Direct calcium channel assay kit on human embryonic kidney (HEK) 293T cells; the Ca^2+^ channel activity was not affected by indole derivatives. However, the positive control, verapamil, prevented the accumulation of Ca^2+^ ions in the cytoplasm. None of the indole derivatives reduced Ca^2+^ channel activity, overcoming the problem that has hampered the development of efflux pump inhibitors (Fig. 3D).

### Lack of toxicity of indole derivatives toward eukaryotic cells

**FIG 4:**
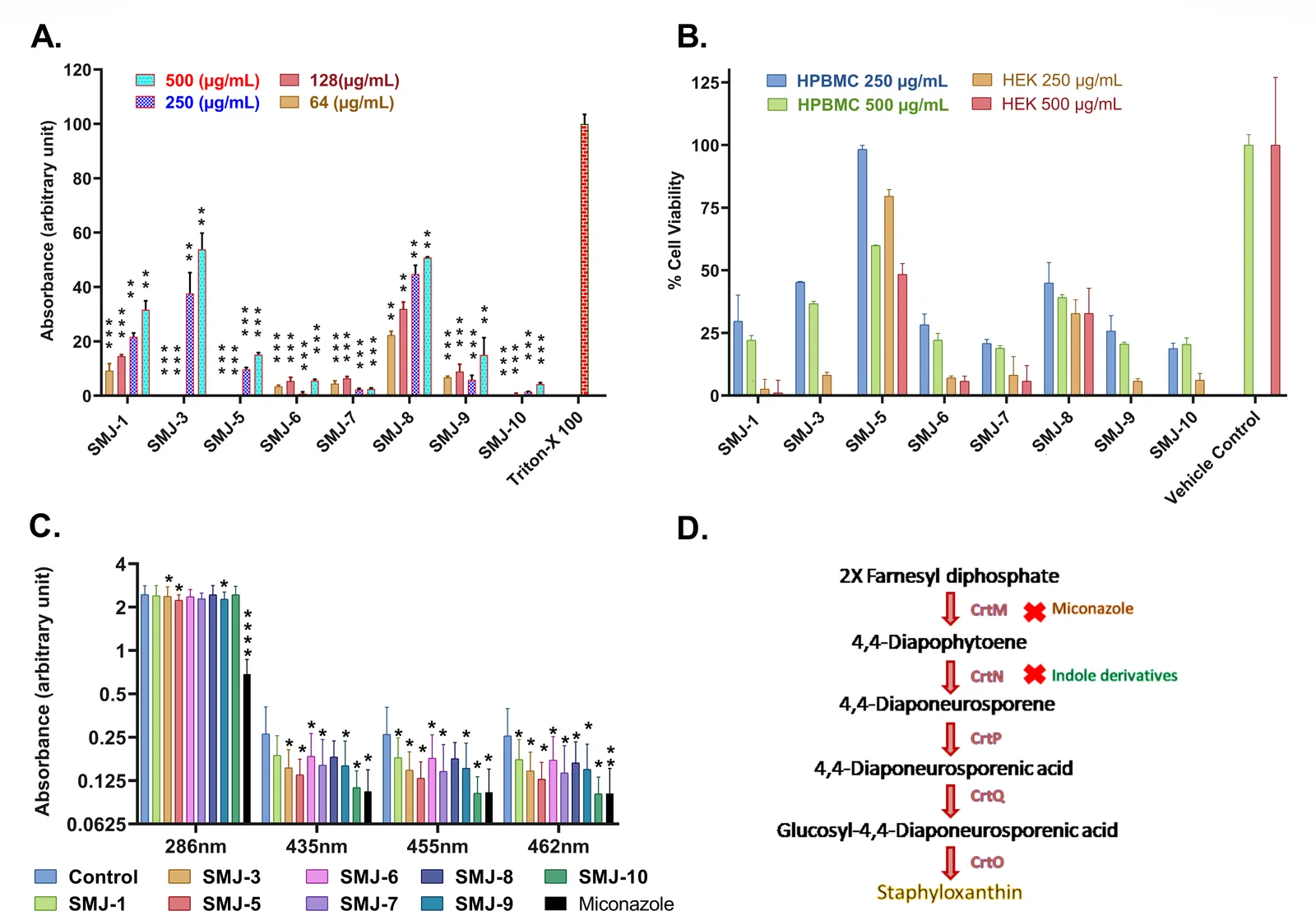
(A) Hemolysis assay on 4% red blood cell (RBC) at 64-, 128-, 250-, and 500-µg/mL concentrations. (**B) **Cytotoxicity assay on human peripheral blood mononuclear cells and human embryonic kidney cells (HEK 293T) at 500- and 250-µg/mL concentrations.** (C) **Spectrometric quantification of staphyloxanthin intermediates, namely, 4,4-diapophytoene (286 nm), 4,4-diaponeurosporene (435 nm), 4,4-diaponeurosporenic acid (455 nm), and staphyloxanthin (462 nm) extracted from control and indole derivatives (1/4 × MIC) treated ATCC 29,213 cells. Results were considered significant (*) when *P* < 0.05 and highly significant (**) when *P* < 0.01 and (***) *P* < 0.001. *P* values were calculated using 95% class interval. Panels A through C represent two biological readings performed in triplicates. (D) Schematic representation of the staphyloxanthin biosynthesis pathway. The figure demonstrates SMJ-5 inhibits dehydrosqualene synthase (CrtM) while miconazole inhibits 4,4-diapophytoene desaturase (CrtN). CrtM is an enzyme that catalyzes the condensation of two farnesyl diphosphates to produce 4,4-diapophytoene, further converted to 4,4-diaponeurosporene by CrtN.

To develop a preliminary idea of the toxicity of indole derivatives, we determined the hemolytic and mammalian cell viability assays. Hemolytic activity was evaluated by measuring the release of hemoglobin from rabbit erythrocytes as a function of concentration. The indole derivatives SMJ-5, SMJ-6, SMJ-7, SMJ-9, and SMJ-10 demonstrated a low hemolytic activity of less than 20% at a significantly higher concentration than their working concentration, whereas SMJ-1, SMJ-3, and SMJ-8 had greater than 20% hemolysis. The resulting dose-response for SMJ-5 was 15% at 500-µg/mL concentration (relatively higher than the effective concentration) (Fig. 4A). Mammalian cell viability assays were conducted on human peripheral blood mononuclear cells (PBMC) and human embryonic kidney cells (HEK 293T) using 3-(4,5-dimethylthiazol-2-yl)-2,5-diphenyltetrazolium bromide (MTT assay at two varying concentrations of 500 and 250 µg/mL. In PBMCs, high cell viability of 59.87 ± 0.21 and 98.31 ± 1.59 for SMJ-5 was observed at 500- and 250-µg/mL concentrations, respectively. In contrast, on HEK 293T cells, SMJ-5 displayed cell viability of 48.35 ± 4.32 and 79.56 ± 2.66 at 500- and 250-µg/mL concentrations, respectively (Fig. 4B).

### Effect of indole derivatives on virulence factors

Staphyloxanthin is a yellow-colored carotenoid and a known virulence factor that promotes resistance to reactive oxygen species and the host immune system found in *S. aureus* (24). Staphyloxanthin and its metabolic intermediates were quantified in the presence and absence of sub-inhibitory concentration of indole derivatives. Indole derivatives were previously reported to inhibit staphyloxanthin formation, and *S. aureus* ATCC 29213 is the standard strain reported to produce staphyloxanthin (24). Therefore, out of curiosity, we also checked the staphyloxanthin inhibiting property of indole derivatives apart from its efflux pump inhibiting property. Hence, we studied the activity of our indole derivatives; we evaluated their activity on all intermediates involved. We found indole derivatives showed a significant reduction in spectrometric measurements of staphyloxanthin biosynthesis pathway metabolic intermediates including 4,4-diaponeurosporene, 4,4-diaponeurosporenic acid, and staphyloxanthin, except 4,4-diapophytoene. SMJ-5, SMJ-7, SMJ-9, and SMJ-10 interfered with the condensation of 4,4′-diapophytoene to 4,4′-diaponeurosporene, indicating these derivatives inhibit CrtN. While the positive control miconazole (25) inhibits staphyloxanthin formation by inhibiting dehydrosqualene synthase (CrtM) (Fig. 4C and D). CrtM catalyzes the condensation of two farnesyl diphosphates to create 4,4-diapophytoene and is the first enzyme in *S. aureus* biosynthetic route for staphyloxanthin, while CrtN catalyzes the formation of 4,4-diaponeurosporene from 4,4-diapophytoene (25). Controlling the carotenoid pigment’s biosynthesis pathway would be very beneficial since it would reduce the persistence of *S. aureus* infections in individuals and make the pathogen more vulnerable to the host immune response.

### Efflux pumps as the target to inhibit biofilm formation and eradicate pre-formed biofilms

Efflux pumps are highly active in bacterial biofilms, making them an excellent target for developing anti-biofilm strategies (26). Encouraged by the potentiation of ciprofloxacin by SMJ-5, we evaluated the effect of the SMJ-5 and ciprofloxacin combination on the *S. aureus* SA-1199B biofilm. The minimum biofilm inhibitory concentration that inhibits ≥50% of the biofilm (MBIC_50_) and minimum biofilm eradication concentration that eradicates ≥50% of the biofilm (MBEC_50_) concentrations of ciprofloxacin were determined. Ciprofloxacin had an MBIC_50_ and MBEC_50_ of 8 and 16 µg/mL, respectively. The MBIC_50_ and MBEC_50_ concentrations of ciprofloxacin, in combination with 1/4× MIC of SMJ-5, demonstrated significant inhibition in biofilm formation and eradication of pre-formed biofilm (16-fold) (Table S7a).

Furthermore, biofilm viability was determined calorimetrically by measuring the reduction in insoluble formazan produced by metabolically active biofilm cells from MTT. SMJ-5 at its sub-inhibitory concentration (1/4× MIC) combined with ciprofloxacin (1 µg/mL) reduced biofilm viability by eightfold, comparable to the reduction seen with ciprofloxacin at 8 µg/mL (Table S7a). Two-fold increase was observed in biofilm inhibition and biofilm eradication capability of ciprofloxacin in presence of SMJ-5 on *S. aureus* SA-1199 strain (Table S7b).

### Confocal microscopy for visualization of biofilms

**FIG 5:**
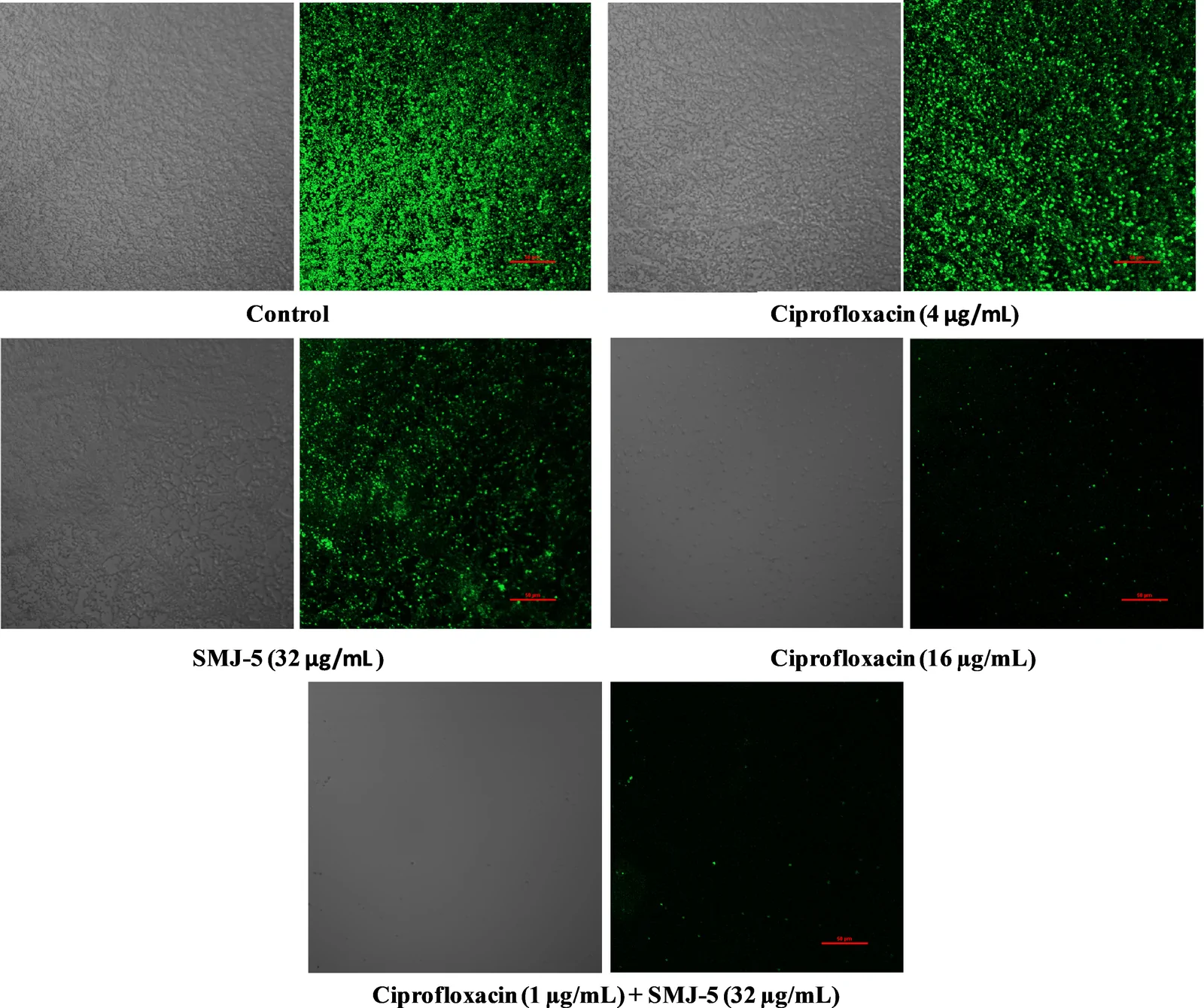
Confocal microscopic images of biofilm disruption in the presence of ciprofloxacin and SMJ-5 alone and in combination after 24 h of treatment on poly-L-lysine-coated glass coverslips. Confocal microscopy of biofilm in the sequence control, ciprofloxacin (4 µg/mL), SMJ-5 (32 µg/mL), ciprofloxacin (16 µg/mL), and ciprofloxacin (1 µg/mL) + SMJ-5 (32 µg/mL). Scale 50 µm. The image is the best representation of the biological triplicates.

The effect of SMJ-5 and ciprofloxacin alone and in combination was assessed using microscopic examination of the *S. aureus* SA-1199B biofilm. Ciprofloxacin alone at 4 µg/mL concentration (1/2× MIC) could not eradicate mature pre-formed biofilm. SMJ-5 (32 µg/mL) combined with ciprofloxacin (1 µg/mL) eradicated the pre-formed biofilm to the same extent as MBEC_50_ of ciprofloxacin alone at 16 µg/mL. Ciprofloxacin’s (1 µg/mL) biofilm eradication efficiency was increased by 16-fold in the presence of SMJ-5. The quantitative data were observed to follow the microscopic study (Fig. 5).

### Enhanced post-antibiotic life of ciprofloxacin in the presence of SMJ-3, 5, 9, and 10

**FIG 6:**
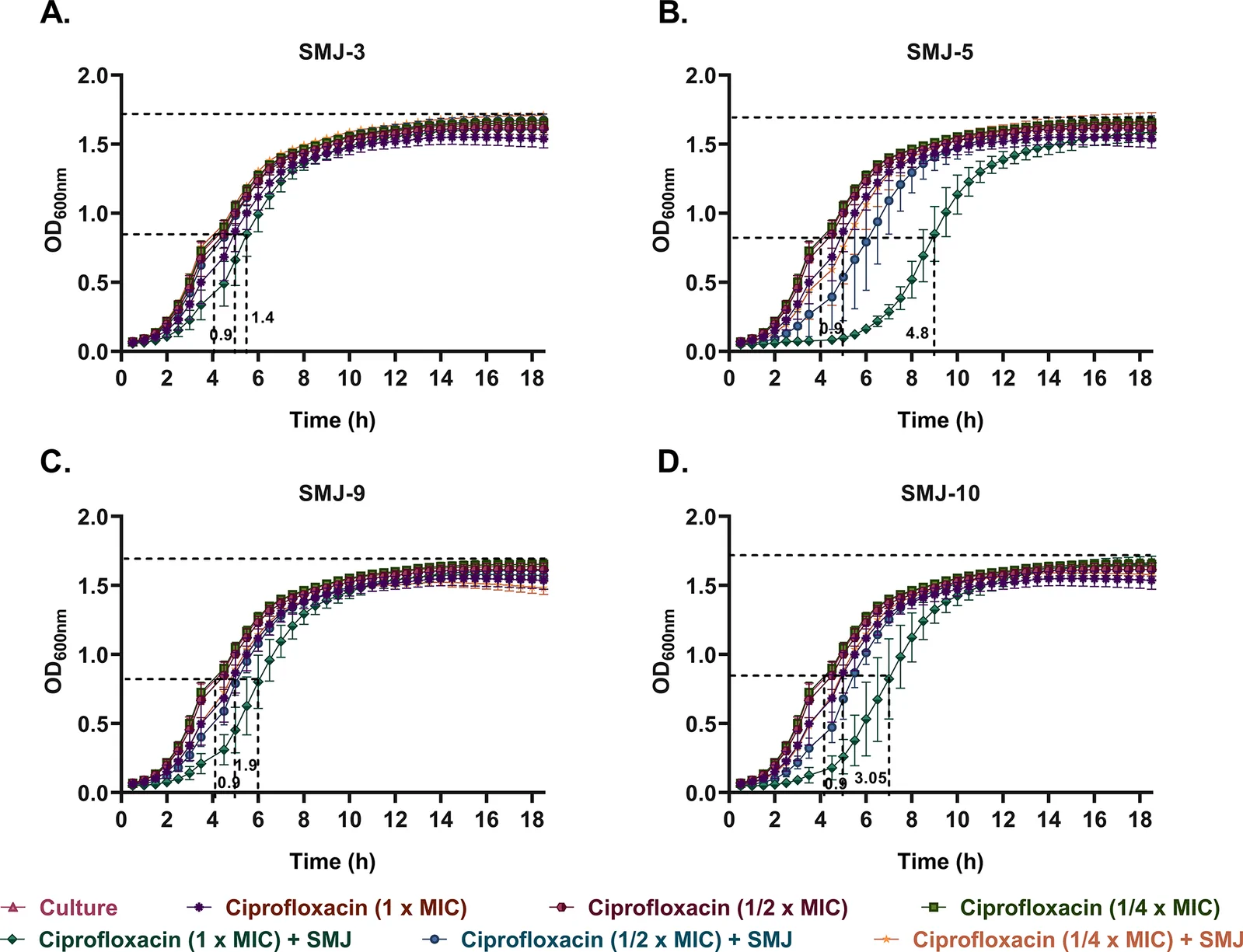
Post-antibiotic effect of ciprofloxacin at 1×, 1/2×, and 1/4× MIC alone and in combination with SMJ-3, SMJ-5, SMJ-9, and SMJ-10 at 1×, 1/2×, and 1/4× MIC by turbiditory method on *S. aureus* SA-1199B strain in H; the results are the representation of two biological repeats performed in triplicates ± SD.

From the above experiments, we evaluated SMJ-3, SMJ-5, SMJ-9, and SMJ-10 to be the best hits, showing low *in vitro* general toxicity in mammalian cells and efficiently eradicating pre-formed biofilm. A post antibiotic effect (PAE) of 0.9 h was observed in an exponential-phase culture of *S. aureus* SA-1199B subjected to 1× MIC of ciprofloxacin alone, while an enhanced PAE of 1.4, 4.8, 1.9, and 3.05 h was observed in combination with SMJ-3, SMJ-5, SMJ-9, and SMJ-10. The combination showed an increase of 0.5, 3.9, 1.0, and 2.15 h compared to when ciprofloxacin was tested alone at 1× MIC. For ciprofloxacin alone and combined with SMJ-3, SMJ-5, SMJ-9, and SMJ-10, an extension of 0.4, 0, 2.0, 0.9, and 1.4 h was seen at 1/2× MIC. Ciprofloxacin did not exhibit any PAE at 1/4× MIC, and among the combinations, SMJ-3, SMJ-5, SMJ-9, and SMJ-10 displayed a PAE of 0, 1.2, 0.8, and 0.9 h. The PAE results demonstrate an extended post-antibiotic life of SMJ-5 compared to SMJ-3, SMJ-9, and SMJ-10 (Fig. 6). Considering the data above, we discovered SMJ-5 as the most potent hit and chose SMJ-5 for further research.

### Mutation frequency analysis of SMJ-5 to check the development of its resistant mutants

A compound’s ability to limit or minimize the development of resistant mutants is represented by its mutation prevention concentration (MPC). The occurrence of mutants anticipates the number of survivors divided by CFU plated. Ciprofloxacin alone displayed an MPC at 16× MIC. In comparison, SMJ-5 at 32 µg/mL (1/4× MIC) reduced the MPC of ciprofloxacin by 64-folds in the *S. aureus* SA-1199B strain, indicating that SMJ-5 holds a clinical relevance in the era of emerging resistance (Table 2).

**TABLE 2:** Mutation prevention concentration of ciprofloxacin alone at various concentrations (0.125 to 16× MIC) and in combination with SMJ-5 at 32 µg/mL on *S. aureus* SA-1199B^*a*^ ^*,^b^*^

Ciprofloxacin (concentration)	0.125× MIC	0.25× MIC	0.5× MIC	1× MIC	2× MIC	4× MIC	8× MIC	16× MIC
Ciprofloxacin (CFU)	UC	UC	UC	UC	UC	6.01 × 10^−11^ ± 1.11 × 10^−11^	0.4 × 10^−10^ ±3.11 × 10^−12^	<10^−10^
Ciprofloxacin + SMJ-5 (32 µg/mL) (CFU)	4.01 × 10^−8^ ±1.32 × 10^−8^	<10^−10^	<10^−10^	<10^−10^	<10^−10^	<10^−10^	< 0^−10^	<10^−10^

^*a*^ Ciprofloxacin (MIC) = 8 µg/mL.

^*b*^ UC, uncountable.

### Effect of indole-based inhibitor SMJ-5 on the time-kill kinetics of ciprofloxacin

**FIG 7:**
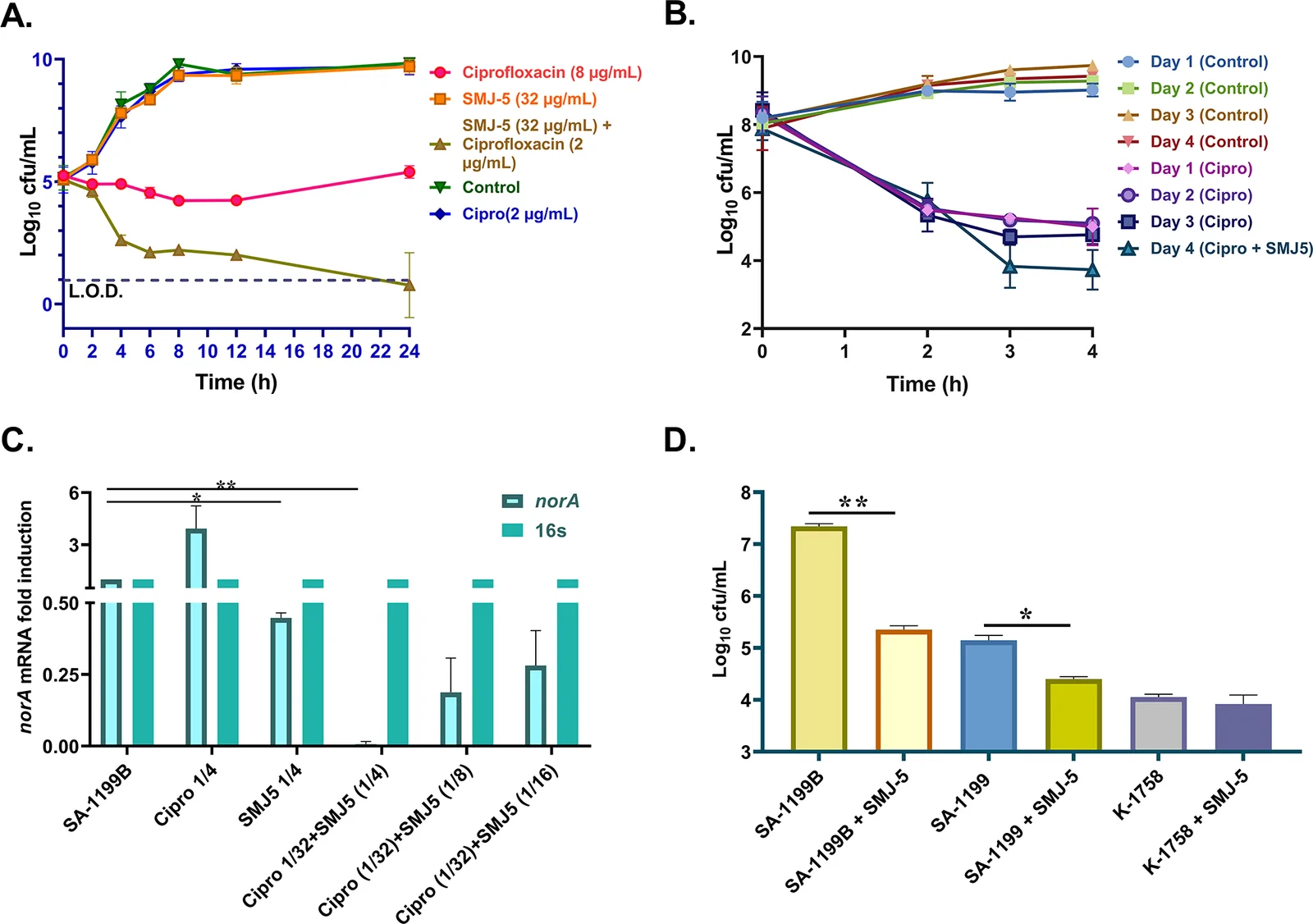
(A) Time-kill kinetic assay, the combination of ciprofloxacin (2 µg/mL) and SMJ-5 (32 µg/mL), demonstrated a bactericidal effect as compared to ciprofloxacin alone against *S. aureus* SA-1199B strain. (**B**) Persister-kill kinetics, ciprofloxacin at 20× MIC, forms persisters hereditarily for 3 days on *S. aureus* ATCC 29213; on the fourth day, combination of ciprofloxacin at 20× MIC and SMJ-5 at 1/4× MIC lowers the persister count by 2 log_10_ CFU/mL. (**C**) RT-PCR of *S.aureus* SA-1199B *norA* in untreated, ciprofloxacin (1/4× MIC), SMJ-5 (1/4× MIC), and ciprofloxacin (1/32 MIC) + SMJ-5 (1/4× MIC), ciprofloxacin (1/32× MIC) + SMJ-5 (1/8× MIC) and ciprofloxacin (1/32× MIC) + SMJ-5 (1/16× MIC). (**D**) Macrophage invasion assay on *S. aureus* SA-1199B (*norA* over-expressing), SA-1199 (*norA* wild type), and K-1758 (*norA* knockout) strain in the presence of SMJ-5 at sub-inhibitory concentration; the results in B correspond to the mean of triplicate readings ± SD. Experiments A and D are representations of three biological repeats, and experiments B and C are the representations of two biological repeats. Results were considered significant (*) when *P* < 0.05 and highly significant (**) when *P* < 0.01 . *P* value calculated using 95% class interval.

The time-kill kinetic study establishes the rate at which a compound kills a micro-organism as a function of survival recorded at various exposure time points. Ciprofloxacin was used in this assay at inhibitory (8 µg/mL) and sub-inhibitory (2 µg/mL) concentrations alone and combined with SMJ-5. Ciprofloxacin and SMJ-5 showed comparable growth to the untreated control cells at sub-inhibitory concentrations. Ciprofloxacin alone at 1× MIC (8 µg/mL) displayed a reduction of 2.3 log_10_ at a 12-h time point but later began to regrow, whereas ciprofloxacin (2 µg/mL) in combination with SMJ-5 (32 µg/mL) reduced CFU by 3.4 log_10_ at the 12-h time point and 4.6 log_10_ at the 24-h time point (Fig. 7A).

### Effect of SMJ-5 on persister heritability

As per the literature, the persisters develop as an epigenetic variant of their wild-type strain; their survival increase does not transfer to the next generation (19, 27). A typical biphasic killing of the cells was observed when an overnight culture of *S. aureus* ATCC 29213 was treated with 20× MIC of ciprofloxacin, leading to a fraction of persisters. The persisters survived every cycle of repeated growth and treatment. The cycle was repeated two times. The results in each cycle were identical to those of the original population, demonstrating that persistence is not heritable under the conditions depicted for the experiment. The persisters did not reduce even after two more cycles. Eradication of stationary-phase persister cells was achieved when they were subjected to ciprofloxacin and SMJ-5 combination treatment. The persister biphasic time-kill kinetic assay revealed the excellent anti-persister potential of the combination, demonstrating a 2 log_10_ reduction compared to ciprofloxacin alone (Fig. 7B).

### SMJ-5 has a transcriptional inhibitory impact on *norA* induction

The expression of the *norA* mRNA was done following a previous study (28). The expression of *norA* mRNA in vehicle-treated bacteria was adjusted to a onefold induction, while ciprofloxacin (1/4× MIC) significantly boosted *norA* expression compared to the vehicle. The expression of *norA* was considerably reduced in the *S. aureus* SA-1199B strain following incubation with SMJ-5 (1/4× MIC), but the addition of ciprofloxacin increased the expression of *norA.* Further, we observed that SMJ-5 (1/4× MIC) in combination with ciprofloxacin (1/32× MIC) significantly reduced the ciprofloxacin-induced expression of *norA* at the mRNA level, aiding in the treatment of *norA*-over-expressed *S. aureus* SA-1199B (Fig. 7C). However, as the concentration of SMJ-5 reduced to 1/8× MIC and 1/16× MIC, the *norA* expression increased, but it was still less than ciprofloxacin-induced expression. A similar pattern was observed with *norA* wild type expressing *S. aureus* SA-1199 strain (Fig. S5A). The results depict that SMJ-5 also inhibits the NorA efflux pump indirectly at the transcriptional level. Further, we wanted to know the effect of SMJ-5 on *mgrA* as *mgrA* is the master regulator of *norA* (29). Moreover, we found that SMJ-5 downregulated *mgrA* expression in a concentration-dependent manner at 1/4× MIC (32 µg/mL), 1/8× MIC (16 µg/mL), and 1/16× MIC (8 µg/mL) (Fig. S5B).

### Role of SMJ-5 to reduce macrophage invasion of NorA over-expressing *S. aureus*

The *S. aureus* strains SA-1199B (NorA over-expressing strain), SA-1199 (wild-type strain), and K-1758 (NorA knockout strain) were assessed for the intracellular invasion of the macrophage THP1 cells (10^5^ cells/well) in the presence or absence of SMJ-5 (32 µg/mL). We found that *S. aureus* SA-1199B had nearly 1.97 log_10_ higher intracellular invasions than the wild-type SA-1199 strain, while *S. aureus* K-1758 had no significant invasion. SMJ-5 (32 µg/mL) did not affect *S. aureus* K-1758 penetration. In contrast, the presence of SMJ-5 (32 µg/mL) reduced the invasion of *S. aureus* SA-1199B to 2.32 log_10_ compared to a 1.02 log_10_ reduction in the wild-type SA-1199 (Fig. 7D).

### Acute toxicity in animal model

**FIG 8:**
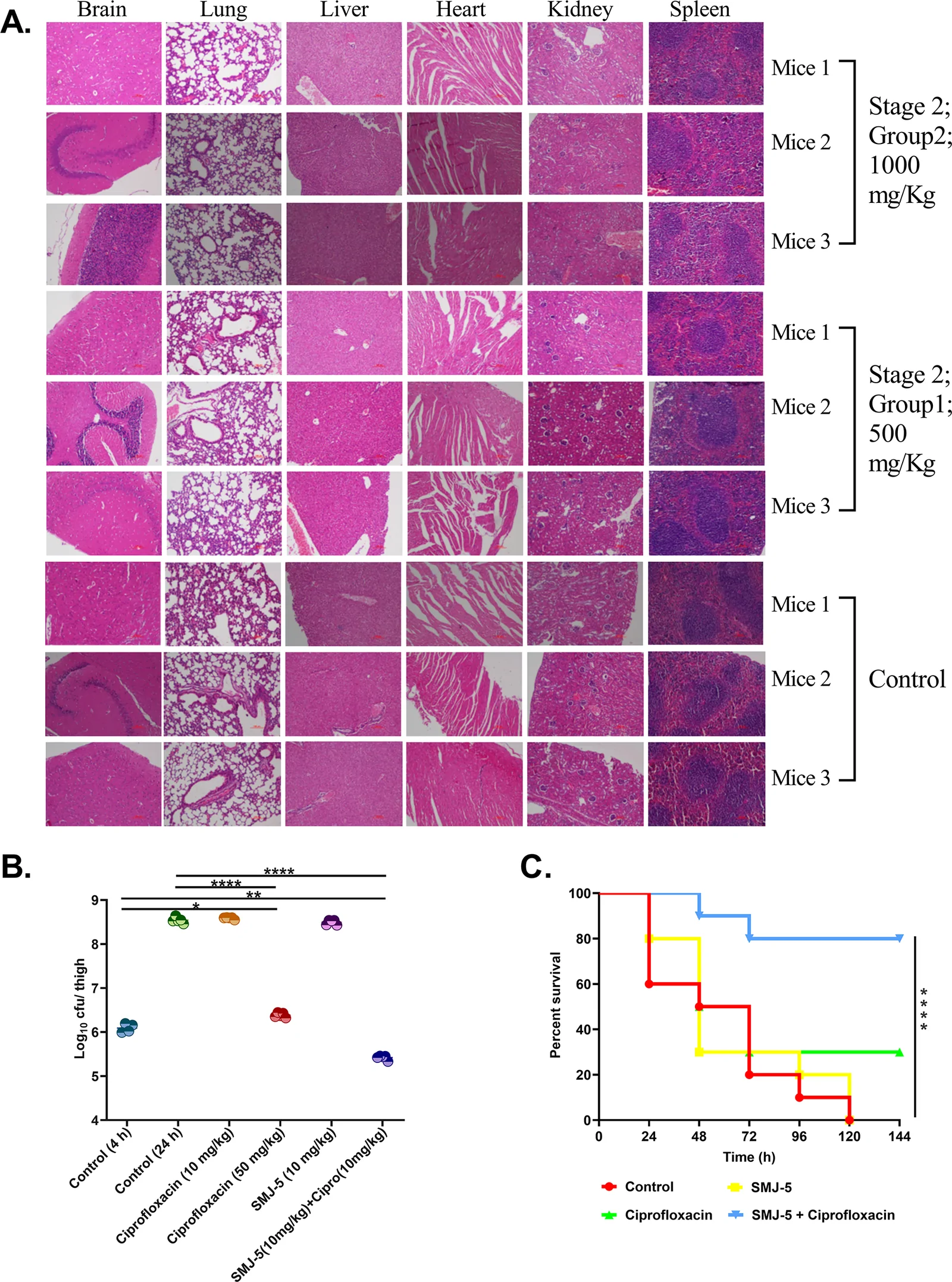
(A) Histopathological analysis of six major organs of BALB/c mice after sub-cutaneous administration of 1,000- and 500-mg/kg SMJ-5 and control, respectively (*n* = 3); and ciprofloxacin-SMJ-5 combination is efficacious in two mouse models of infection. (**B**) Neutropenic mouse thigh infection model: single sub-cutaneous dose treatment (10-mg/kg ciprofloxacin and 10-mg/kg SMJ-5 alone and in combination, six mice per group, 4 h after infection). CFU was determined 24 h post-infection for drug-treated mice; for controls, CFU was calculated 4 and 24 h post-infection. (**C**) Mouse peritonitis survival model: three sub-cutaneous dose treatments (10-mg/kg ciprofloxacin and 10-mg/kg SMJ-5 alone and in combination, 10 mice per group; after 1, 4, and 6 h post-infection). *P < 0.05, **P < 0.01, ****P < 0.0001. *P* values were calculated using 95% class interval.

Unwanted effects that cause biochemical lesions and functional impairments in organs can change a person’s overall functioning or the functioning of specific organs (30, 31). The positive *in vitro* toxicity results on mammalian cell lines prompted us to investigate SMJ-5’s *in vivo* acute toxicity to understand the compound’s therapeutic potential better. SMJ-5 was administered sub-cutaneously in two stages: stage 1 consisted of three groups and stage 2 consisted of two groups at a dose of 50 mg/kg (stage 1, group 1), 100 mg/kg (stage 1, group 2), and 250 mg/kg (stage 1, group 3) and in stage 2 and at a dose of 500 mg/kg (stage 2, group 1), and 1000 mg/kg (stage 2, group 2) (Table S8). The experiment was performed in triplicates. None of the animals died during the administration of SMJ-5, and no major change in body weight or blood glucose levels was observed after 7 days of administration. The preliminary acute toxicity study states that SMJ-5 is safe at ≥1,000 mg/kg, which can be further studied for better accuracy. The behavioral pattern of treated mice was the same as that of the placebo group. The mice were sacrificed for histopathological examination, and blood was collected through the retro-orbital route for biochemical tests. Histopathological analyses displayed similar results to the control group’s (Fig. 8A). The renal function test of treated mice resulted in normal calcium, blood urea, serum creatinine, and uric acid level in the body, similar to that of the control group. Liver function test demonstrated that the treated mice’s bilirubin (total); bilirubin, direct (conjugated); bilirubin, indirect (unconjugated); serum glutamic-oxaloacetic transaminase [S.G.O.T. (ALT)] , serum glutamic-pyruvic transaminase [S.G.P.T. (ALT)], total protein, albumin, globulin, A:G ratio, and total cholesterol were all within normal limits (Table S9).

### Murine *in vivo* thigh infection model

The high efficacy of the combination prompted us to evaluate the effect of SMJ-5 and ciprofloxacin combination on the soft-tissue infection model in mice. Using a neutropenic mouse thigh infection model, we assessed the *in vivo* effectiveness of the SMJ-5 and ciprofloxacin combination. Mice were given 1.865 × 10^6^ CFU of *S. aureus* SA-1199B infection intramuscularly. After 4 h, the first control group was sacrificed to check if the infection was established successfully, then the right thigh of the mice was homogenized and plated. The CFU count in the control group sacrificed at 4 h was found to be approximately 1.44 × 10^6^. Ciprofloxacin (10 mg/kg) and SMJ-5 (10 mg/kg) alone, administered 4 h after infection, did not lower the bacterial counts 20 h after treatment. However, the combination of SMJ-5 and ciprofloxacin administered 4 h after infection proved very efficacious, demonstrating a reduction of approximately 0.8 and 3.191 log_10_ compared to 4 and 24 h of untreated control (Fig. 8B).

### Effect of the combination on the mouse peritonitis model

A mouse septicemia model was used to perform an animal efficacy study. To initiate septicemia, mice were infected intraperitoneally with 500 µL of 2 × 10^9^ CFU/mL of *S. aureus* SA-1199B, a dose that leads to ≥90% mortality. SMJ-5 (10 mg/kg) and ciprofloxacin (10 mg/kg) alone and in combination were injected three times in a single day at 1, 3, and 5 h after infection. SMJ-5-treated mice did not survive the infection after 5 d, whereas 30% of ciprofloxacin-treated mice survived, and the group treated with the combination of SMJ-5 and ciprofloxacin cured 80% of the animals, that is, the survival increased to 50% in the combination compared to ciprofloxacin alone (Fig. 8C).

## DISCUSSION

*S. aureus* is an opportunistic, commensal bacterium that colonizes about one-third of the entire population asymptomatically (32) and successfully establishes infections using efflux pumps, genetic composition, and virulence factors (33). Efflux pumps have majorly contributed to the development of antibiotic resistance. In *S. aureus*, the NorA efflux pump is linked to fluoroquinolone resistance (10, 34, 35). NorA efflux pumps of the major facilitator superfamily play an essential role in innate and acquired antibiotic resistance in Gram-positive bacteria by extruding a myriad of anti-microbial metabolites (36). Previous studies have demonstrated that the lack of efflux pumps plays an essential role in attenuating the bacterial biofilm and virulence of bacteria and successfully treating drug-resistant infections (37).

An EPI 2-(2-aminophenyl) indole was reported by our group that was isolated from the terrestrial bacterial isolate, *Streptomyces* sp. IMTB 2501 (34). Using the scaffold as the rational, we successfully synthesized eight indole derivatives to improve their efficacy and activity profile. In the current study, we evaluated the efflux pump inhibitory potential of indole derivatives to enable better therapeutic intervention. This study demonstrates the remarkable efficacy of indole derivative SMJ-5 in potentiating fluoroquinolones in *norA* over-expressing *S. aureus*. To ensure that indole derivatives cause no bacterial death, the synergy with ciprofloxacin and norfloxacin is observed at sub-inhibitory concentrations of indole derivatives. In the presence of a potentiator, a minimum fourfold decrease in the MICs from their initial values was regarded as evidence of efflux inhibition (38). In the *norA* over-expressing strain, SMJ-5 synergized with ciprofloxacin and norfloxacin, resulting in lower MICs than the clinical resistance breakpoints of >4 µg/mL of ciprofloxacin and ≥16 µg/mL of norfloxacin (17, 18). In addition, SMJ-5 with ciprofloxacin showed synergy in 20 clinical strains of *S. aureus*. SMJ-3, SMJ-5, SMJ-9, and SMJ-10 show impressive EPI activity in the EtBr accumulation and inhibition assays, which is the gold standard for evaluating EPI functionality (34). The target of our EPIs, the NorA efflux pump, was partially validated by observing a more significant norfloxacin accumulation in the presence of indole derivatives compared to the control.

A chemical entity must complete a stringent checklist to be an EPI. The compound must not be anti-microbial; an anti-bacterial compound eventually results in the selection of resistant mutants, limiting its worth as an EPI. A true EPI should not hinder general metabolic processes limiting resistance development. Carbonyl cyanide *m*-chlorophenyl hydrazone is arguably the most well-known laboratory EPI (22). However, it is unlikely that such compounds could turn into medications because they can also be toxic. Thus, the clinical usage of efflux pump inhibitors should not impart toxicity on their own (39). Therefore, to rule out the possibility of any other effect on the bacterial membrane by the indole derivatives, we evaluated the effect of indole derivatives on general metabolic functions of bacterial cells, such as membrane depolarization, membrane permeability, ATP depletion, or antagonizing effects on the activation of eukaryotic Ca^2+^ channels as a corollary. Compounds disturbing the membrane can lead to resistance development in bacteria and identification of false EPIs, such as verapamil and CCCP (22). Among the four major hits, only SMJ-5, SMJ-9, and SMJ-10 do not disturb these metabolic functions and demonstrate the solidity of indole derivatives as true NorA efflux pump inhibitors.

Secondly, a true EPI must have excellent pharmacological characteristics such as low toxicity, high therapeutic and safety indices, and a favorable absorption, distribution, metabolism, excretion, and toxicity) profile (22). The known EPIs, despite being strong inhibitors, could not proceed to clinical trials due to their unavoidable toxicities. Reserpine, a plant alkaloid, is a general inhibitor of gram-positive efflux pumps, but its clinical utility is limited because later, it was found to be neurotoxic (40). In Gram-positive *Staphylococci* and Gram-negative *Campylobacter* spp., an EPI epigallocatechin gallate has been shown to increase the potency of tetracycline, erythromycin, and ciprofloxacin. However, due to toxicity concerns, no additional *in vivo* or pre-clinical trials were conducted (22). However, here, we found SMJ-5 to be negligibly hemolytic and displayed the highest viability in mammalian PBMC and HEK cell lines and was identified as a safe candidate for future investigation.

Efflux pumps are also known to contribute to biofilm development, and EPIs, such as thioridazine, boeravinone B, and nilotinib, are reported to reduce biofilm formation (10, 26, 41). As a corollary, we investigated the efficacy of indole derivatives in inhibiting biofilm formation and eradicating pre-formed biofilms. In crystal violet and MTT biofilm disruption assays, SMJ-3, SMJ-5, SMJ-7, SMJ-9, and SMJ-10 at sub-inhibitory concentrations successfully increased the efficacy of ciprofloxacin to eradicate the pre-formed *S. aureus* biofilm. We also used confocal microscopy to confirm SMJ-5’s involvement in biofilm eradication after being influenced by its biofilm eradication capabilities.

Hence, considering *in vitro* toxicity data, hemolysis data, and biofilm eradication capability results, we evaluated the post-antibiotic effect of ciprofloxacin in the presence of indole derivatives. An antibiotic agent produces PAE, per a prior study, when it results in an instantaneous growth delay of at least 0.5 h on a bacterial culture (42). SMJ-5 demonstrated a significant 3.9-h prolonged life of ciprofloxacin that may aid in maintaining wide dosage intervals. Given these findings, we decided to pursue SMJ-5 for further research.

The bacterial resistance to almost all antibiotics has led to the current state of anti-microbial resistance. To prevent the emergence of resistance to them, it is possible to use ideal EPIs in combination with them to have better therapeutic efficacy. In particular, it has been demonstrated that the non-anti-bacterial adjuvant that blocks the resistance mechanism of that antibiotic can reduce the development of resistance (43). Here we found a significantly high reduction of 64-fold in MPC of ciprofloxacin in the presence of SMJ-5 compared to that of ciprofloxacin alone during mutation frequency analysis demonstrating concurrent therapy to minimize resistance development. A reduction of 2 log_10_ CFU/mL in time-kill kinetics is referred to as synergy relative to its most active counterpart (here, ciprofloxacin). Moreover, it must be ≥2 log_10_ CFU/mL below the initial inoculum (44). The CFU decrease in combination was 2.3 log_10_ lower than ciprofloxacin alone at the 12-h time that establishes synergy. Anti-microbial persisters appear to be formed through multifactorial pathways involving efflux pumps, protein synthesis inhibition, and impaired cell development (19). As a result, we surmised that our EPIs might reduce the number of persister cells. Compared to ciprofloxacin alone, the combined therapy showed a 1 log_10_ reduction in persister cells, proving its efficacy. Indole derivatives are known to inhibit staphyloxanthin and its intermediates (24), which was validated when we found inhibition of staphyloxanthin and its intermediates by SMJ-5 at its sub-inhibitory concentration. Although *S. aureus* is not typically thought of as an intracellular pathogen, it might have a chance to survive and spread disease if it were to occupy an intracellular niche (32) momentarily. In this regard, our investigations supported the role of NorA overproduction in *S. aureus* intracellular invasion in macrophage THP-1 cell lines. Additionally, SMJ-5’s activity as an EPI in preventing NorA-overproducing *S. aureus* invasion was validated. The THP-1 macrophage invasion of *norA*-over-expressed *S. aureus* was observed to reduce by 2.32 log_10_ in the presence of SMJ-5, whereas no macrophage invasion was observed in the case of *norA* deletion *S. aureus.* Additionally, RT-PCR demonstrated that SMJ-5 dramatically decreased *norA*’s expression at the mRNA level after ciprofloxacin induction, indicating indirect inhibition of the NorA efflux pump by SMJ-5. Moreover, as it is known that *norA* is regulated by its master regulator *mgrA* (29), therefore we hypothesized SMJ-5 would also downregulate the *mgrA* gene. This was then proven by RT-PCR analysis that SMJ-5 downregulates *mgrA* expression. The outstanding *in vitro* results prompted us to assess the *in vivo* efficacy of the combination.

The *in vivo* effectiveness study was then conducted, and we found a significant reduction in CFU compared to the control. Also, the combination showed a 50% higher survival rate than ciprofloxacin alone in a mouse peritonitis model. *In vivo*, an acute toxicity study displayed that the safety of SMJ-5 is ≥1,000 mg/kg. No significant histopathological changes were observed.

Our findings conclude that SMJ-5 is a new compound of bacterial EPI that should be investigated further for its clinical therapeutic potential in conjunction with fluoroquinolones as a therapeutic drug.

## MATERIALS AND METHODS

### General chemistry

All chemicals and reagents used for the synthesis were procured from Sigma Aldrich (St. Louis, MO, USA), Alfa-aesar (Johnson Matthey Company, Ward Hill, MA, USA), and SDFineChem Ltd (India). Silica gel (#100–200) was used for column chromatography (CC), and thin layer chromatography (TLC) plates were purchased from Merck (Germany). The organic solvents used for CC were obtained from Finar and Rankem (India). ^1^H and ^13^C NMR spectra were recorded on BrukerAvance DPX 400 spectrometer (Brukers, Germany) at 400 and 100 MHz, and ^1^H spectra were also recorded for a few compounds on JNM-ECA 500 series NMR JEOL spectrometer (JEOL, Japan) at 500 MHz, respectively. NMR spectra were recorded in CDCl_3_, MeOD, and/or DMSO-*d*_6_ at room temperature. Chemical shifts values (*δ*) were expressed in parts per million (ppm) relative to internal standard tetramethylsilane. Coupling constants (*J*) are given in hertz. Data are reported in the following order: chemical shift, multiplicity (s = singlet, d = doublet, t = triplet, dd = doublet of doublets, dt = doublet of triplets, m = multiplet, br = broad), and combinations of these coupling constants and integration. The mass spectra were recorded on a Thermo LTQ-XL spectrometer and Agilent 6200 series TOF/6500 series Q-TOF 10.1.

### General procedure for the synthesis of 2-(2′-aminophenyl) indole [RP2] derivatives

**FIG 9:**
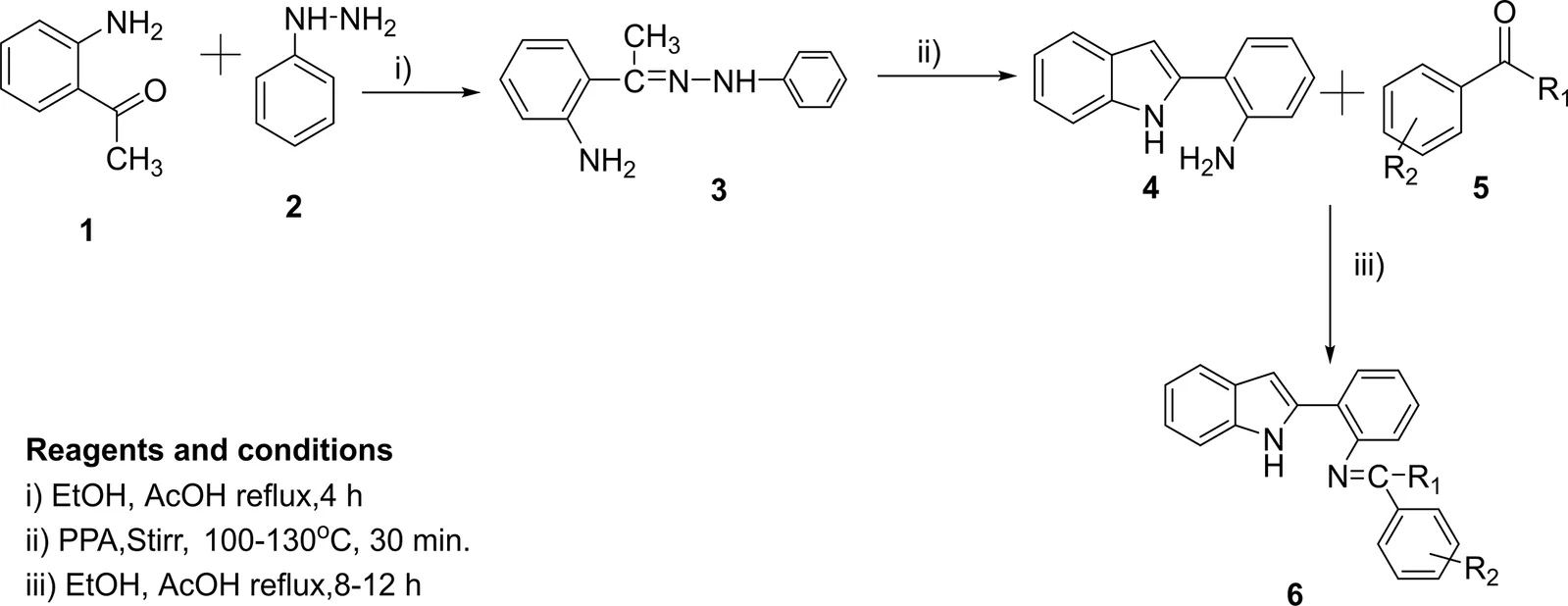
Protocol for the synthesis of 2-(2′-aminophenyl) indole [RP2] derivatives.

2-(2′-Aminophenyl)indole [4] was synthesized as per the method reported by Agarwal et al. (45). In brief, to a round-bottom flask containing phenyl hydrazine [2] (11.09 mmol) in absolute ethanol, *o*-aminoacetophenone [1] (7.3 mmol) was added with the addition of 5–10 drops of glacial acetic acid. The reaction mixture was refluxed for 4 h. After cooling, the precipitates (ppt’s) of phenylhydrazone [3] were washed with ethanol to give a solid orange color. Final cyclization was done by pouring phenylhydrazone [3] portionwise to the heated solution of polyphosphoric acid and stirred vigorously for 30 min at 80°C. The resulting mixture was cooled to room temperature and poured over crushed ice containing sodium hydroxide pellets. The solid ppt’s formed were washed with water and dried to afford crude product. Final purification was done using column chromatography over silica gel (ethyl acetate/*n*-hexane: 1.5/8.5) to give the desired product [4] (Fig. 9).

The subsequent step included synthesizing mono-2-(2-arylideneaminophenyl)indole derivatives [6] under reflux settings using various aromatic aldehydes/acetophenones [5] (46). The substituted aromatic aldehyde/acetophenone [5] (2.88 mmol) was introduced dropwise to a round-bottom flask containing 2-(2′-aminophenyl) indole [4] (2.88 mmol) in absolute ethanol. To catalyze the reaction, a few drops of glacial acetic acid were added. The reaction was maintained under reflux conditions for 8–12 h until the completion of the reaction. Completion of reaction was monitored by TLC until the starting material was consumed. After cooling, the mixture was extracted with ethyl acetate and washed with brine. The combined organic layer was dried over anhydrous sodium sulfate, and the solvent was evaporated under a vacuum. The resultant combination was purified using *n*-hexane-ethyl acetate gradient (*n*-hexane: 100% to *n*-hexane/ethyl acetate: 50/50) on a silica gel column chromatography to give the pure final product (Table S1).

### RP2

2-(2′-Aminophenyl) indole; white crystals; yield 74%; ^1^H NMR (500 MHz, CDCl_3_) *δ* ppm 8.45 (br s, 1 h), 7.63 (d, 1H, *J* = 7.5 Hz), 7.39 (t, 2 h, *J* = 7.5 Hz),7.21–7.12 (3H, m), 6.86 (m, 1 h), 6.81 (d, 1 h, *J* = 7.5 Hz),6.72 (m, 1 h), 3.15 (br s, 2 h); Electrospray ionization-mass spectrometry (ESI-MS) *m*/*z* calculated for C_14_H_13_N_2_ [M + H]^+^209.10, found 209.08.

#### SMJ-1

(E)−2-(1H-indol-2-yl)-N-(1-(p-tolyl)ethylidene)aniline; tan color solid; yield 82%; ^1^H NMR (400 MHz, DMSO-*d*_6_) *δ* ppm: 11.39 (s, 1 h), 7.46 (d, *J* = 7.5 Hz, 1 h), 7.35 (t, *J* = 8.7 Hz, 3 h), 7.25 (d, *J* = 7.8 Hz, 1 h), 7.02 (m, 3H), 6.94 (m, *J* = 8.9 Hz, 1 h), 6.87 (t, *J* = 15.0 Hz, 1 h),6.66 (m, 1 h), 6.54 (m, 2 h), 2.21 (s, 3 h), 2.05 (s, 3 h); ^13^C NMR (100 MHz, DMSO*-d*_6_) *δ* ppm: 19.0, 20.9, 30.0, 56.5, 58.5, 111.6, 113.2, 113.2, 113.7, 116.1, 119.3, 119.5, 121.3, 121.3, 125.7, 126.1, 128.7, 128.8, 131.1, 135.6, 137.7, 144.2, 147.3; ESI-HRMS *m*/*z*; calcd for C_23_H_21_N_2_[M + H] ^+^325.1705; found: 325.1704.

#### SMJ-3

(E)−4-(1-((2-(1H-indol-2-yl)phenyl)imino)ethyl)phenol; white color solid; yield 76%; ^1^H NMR (400 MHz, DMSO-*d*_6_) *δ* ppm: 11.35 (s, 1 h), 9.25 (s, 1 h), 7.45 (d, *J* = 7.6 Hz, 1 h), 7.31 (m, 3 h), 7.16 (d, *J* = 8 Hz, 1 h), 7.0 (t, *J* = 7.6 Hz, 1 h), 6.93 (t, *J* = 8 Hz, 1 h), 6. 84 (t, *J* = 15.2 Hz, 1 h), 6.63 (m, 3 h), 6.54 (t, *J* = 8 Hz, 1 h), 6.47 (s, 1 h), 2. 0 (s, 3 h);^13^C NMR (100 MHz, DMSO-*d*_6_) *δ* ppm: 30.5, 46.6, 58.2, 111.5, 113.2, 113.6, 114.8, 115.6, 115.9, 119.3, 119.4, 121.3, 121.4, 125.7, 127.5, 128.6, 130.9, 131.2, 137.7, 140.7, 144.2, 156.1; Electrospray ionization-high resolution mass spectrometry (ESI-HRMS) *m*/z; calcd for C_22_H_19_N_2_O [M + H] ^+^327.1497; found: 327.1494.

#### SMJ-5

(E)−5-(1-((2-(1H-indol-2-yl)phenyl)imino)ethyl)benzene-1,3-diol; blonde color; yield 91%; ^1^H NMR (400 MHz, DMSO-*d*_6_) *δ* ppm: 11.34 (s, 1 h), 9.01 (s, 2 h), 7.46 (d, *J* = 6.7 Hz, 1 h), 7.34 (d, *J* = 8.0 Hz, 1 h), 7.29 (d, *J* = 7.9 Hz, 1 h), 7.04 (t, *J* = 7.2 Hz, 1 h), 6.94 (dd, *J* = 1.3, 15.3 Hz, 1 h), 6.89 (m, 1 h), 6.66 (d, *J* = 7.7 Hz, 1 h), 6.55 (t, *J* = 7.4 Hz, 1 h), 6.47 (s, 1 h), 6.37 (d, *J* = 2.1 Hz, 2 h), 6.01 (t, *J* = 2.0 Hz, 1 h), 1.98 (m, 3 h); ^13^C NMR (100 MHz, DMSO-*d*_6_)*δ* ppm: 170.3, 157.6, 151.2, 143.7, 137.2, 130.5, 128.1, 125.2, 120.8, 120.7, 118.9, 118.9, 115.3, 113.0, 104.3, 100.43, 58.1, 48.4, 29.4, 28.9, 20.7, 17.2, 17.1; D_2_O exchange confirmed the presence of NH proton; ESI-HRMS *m*/z; calcd forC_22_H_18_N_2_O_2_[M + H] ^+^343.1447; found: 343.1446.

#### SMJ-6

(E)-N-(4-fluorobenzylidene)-2-(1H-indol-2-yl)aniline; white color solid; yield 54%; ^1^H NMR (400 MHz, DMSO-*d*_6_) *δ* ppm: 12.9 (s, 1 h), 8.60 (d, *J* = 1.0 Hz, 1 h), 8.58 (d, *J* = 1.2 Hz, 1 h), 8.15 (d, *J* = 0.8 Hz, 1 h), 7.90 (m, 3 h), 7.76 (m, 4 h), 7.54 (d, *J* = 8.0 Hz, 1 h), 7.47 (m, 4H) 7.17 (m, 1 h); ^13^C NMR (100 MHz, DMSO *d*_6_) *δ* ppm: 163.7, 154.3, 144.9, 141.0, 139.0, 137.1, 131.1, 131.0, 129.3, 128.5, 125.6, 125.4, 121.9, 121.6, 120.9, 120.3, 116.2, 115.4, 115.2, 111.9, 111.8.; ESI-HRMS *m*/*z*; calcd for C_21_H_14_FN_2_^+^313.1141; found: 313.1141.

#### SMJ-7

(E)−2-(1H-indol-2-yl)-N-(1-(4-nitrophenyl)ethylidene)aniline; orange color solid; yield 81%; ^1^H NMR (400 MHz, DMSO-*d*_6_) *δ* ppm: 11.54 (s, 1 h), 8.13 (d, *J* = 8 Hz, 2 h), 7.73 (d, *J* = 8 Hz, 2 h), 7.5 (d, *J* = 4 Hz, 1H), 7.36 (t, *J* = 8 Hz, 2 h), 7.06 (t, *J* = 7.2 Hz,1H), 6.98 (m, 1H),6.92 (t, *J* = 8 Hz, 1 h), 6.84 (s, 1H),6.7 (d, *J* = 8 Hz, 1 h), 6.6(t, *J* = 7.2 Hz, 1 h), 2.14 (s, 3H); ^13^C NMR (100 MHz, DMSO-*d*_6_) *δ* ppm: δ156.8, 145.9, 143.2, 137.3, 130.9, 128.5, 126.9,126.9, 124.9, 123.2,123.2, 121.2, 119.4, 118.6, 116.2, 113.2, 112.9, 111.3, 111.2, 58.4, 29.1; ESI-HRMS *m*/*z*; calcd for C_22_H_17_N_3_O_2_[M + H]^+^356.1399; found: 356.1393.

#### SMJ-8

(E)−2-(1H-indol-2-yl)-N-(4-trifluoromethyl)benzylidene)aniline; off-white solid; yield 77%; ^1^H NMR (400 MHz, DMSO-*d*_6_) *δ* ppm: 12.97 (s, 1 h), 8.6 (d, *J* = 8 Hz, 1H), 8.15 (d, *J* = 8 Hz, 1 h), 8.08 (m, 2 h), 8.0 (m, 2 h), 7.82–7.7 (m, 3 h), 7.4 (m, 2 h), 7.18 (t, *J* = 8 Hz, 1 h); ^13^C NMR (100 MHz, DMSO*-d*_6_) *δ* ppm:154.2, 145.3, 145.2, 141.6, 139.6, 130.3, 129.9, 129.8, 129.6, 129.2, 126.5, 126.2, 126.0, 125.9, 125.8, 123.5, 122.5, 121.7, 121.3, 120.9, 161.8, 112.5, 112.2; ESI-HRMS *m*/*z*; calcd for C_22_H_14_F_3_N_2_^+^, 363.1109; found: 365.1109.

#### SMJ-9

(E)−2-(1H-indol-2-yl)-N-(1-(4-(methylthio)phenyl)ethylidene)aniline; cream color solid; yield-82%; ^1^H NMR (400 MHz, DMSO-*d*_6_) *δ* ppm:11.40(s, 1 h) 7.47 (d, *J* = 6.7 Hz, 1 h), 7.41 (d, *J* = 8.5 Hz, 2 h), 7.35 (d, *J* = 8.0 Hz, 1 h), 7.28 (d, *J* = 8.0 Hz, 1 h), 7.14 (d, *J* = 8.5 Hz, 2 h), 7.03 (t, *J* = 7.2 Hz, 1 h), 6.95 (m, 1 h), 6.89 (t, *J* = 7.5 Hz, 1 h), 6.67 (d, *J* = 7.7 Hz, 1 h), 6.57 (m, 2 h), 2.4 (s, 3 h), 2.1 (s, 3 h); ^13^C NMR (100 MHz, DMSO-*d*_6_) *δ* ppm: 147.1, 144.1, 137.7, 136.1, 131.2, 128.7, 126.8, 126.8, 126.1, 126.0, 125.6, 121.5, 121.4, 119.5, 119.2, 116.1, 113.7, 113.26, 112.9, 111.6, 58.4, 29.8, 15.3; ESI-HRMS *m*/*z*; calcd for C_23_H_21_N_2_S [M + H]^+^ 357.1424; found: 357.1424.

#### SMJ-10

E)-N-(1-(4-aminophenyl)ethylidene)−2-(1H-indol-2-yl)aniline; cream color solid; yield 58%; ^1^H NMR (400 MHz, DMSO-*d*_6_) *δ* ppm:11.29 (s, 1 h), 7.45 (d, *J* = 7.1 Hz, 1 h), 7.32 (d, *J* = 8.0 Hz, 1 h), 7.15 (m, 3 h), 7.00 (t, *J* = 7.3 Hz, 1 h), 6.92 (m, 1 h), 6.83 (m, 1 h), 6.63 (d, *J* = 7.8 Hz, 1 h), 6.53 (t, *J* = 7.3 Hz, 1 h), 6.45 (d, *J* = 8.5 Hz, 2 h),6.35 (s, 1 h), 4.89 (s, 2 h), 1.96 (s, 3 h; ^13^C NMR (100 MHz, DMSO-*d*_6_) *δ* ppm: 128.5, 127.1, 121.3, 121.2, 119.4, 119.2, 115.7, 113.5, 113.1, 111.5, 30.0; ESI-HRMS *m*/*z*; calcd for C_22_H_20_N_3_[M + H]^+^ 326.1657; found: 326.1659.

### Bacterial strains, cell lines, and growth medium

The *S. aureus* SA-1199B with A116E GrlA substitution, over-expressing the NorA MDR efflux pump, SA-1199 (*norA* wild-type), SA-K-1758 (NCTC 8325–4 *norA* deletion mutant)*,* ATCC 33591, ATCC 29213 and 20 clinical isolates of *S. aureus* from GMCH were used in the present study. Human peripheral blood mononuclear cells (Himedia), THP-1 human monocytic cell line, and HEK-293 cells were used for *ex vivo* experiments. For the generation of macrophages, THP-1 cells were induced by phorbol 12-myristate 13-acetate at 100 ng/mL and incubated for 24 h.

All bacterial media and supplements included cation-adjusted BBL Mueller-Hinton II Broth (CA-MHB), Mueller-Hinton agar (MHA), and tryptic soya broth (TSB). Dulbecco's Modified Eagle Medium (DMEM) (ThermoFisher) and Fetal bovine serum (FBS) (Thermo Fisher) were used for cell culture experiments.

### MIC determination and synergy assay

The minimum inhibitory concentration was determined using broth microdilution according to the CLSI standards (47, 48). The experiment was conducted in 96-well round-bottom plates with a 200-µL total reaction volume. Twofold serial dilutions of the drugs [ciprofloxacin (256–2 µg/mL) and norfloxacin (256–2 µg/mL)] were prepared. The OD_600nm_ of the bacteria grown to the mid-exponential phase was adjusted to 0.25–0.3, and it was diluted such that each well finally contained 5 × 10^5^ CFU/mL, and the plates were incubated at 37°C for 18 h.

The synergistic efficacy of indole derivatives with norfloxacin (Sigma Aldrich) and ciprofloxacin (Sigma Aldrich) was identified by checkerboard assay (47, 48). The MIC of antibiotics and the indole derivatives were determined by varying the concentration of the indole derivatives and the drugs’ varying concentrations. The fractional inhibitory concentration index was calculated to identify the synergistic efficacy of indole derivatives using the following equation:

FICI = MIC (antibiotic in the presence of EPI) / MIC (antibiotic alone) + MIC (EPI in the presence of antibiotic) / MIC (EPI alone).

The FICI values of ≤0.5, >0.5–4, and >4 were considered to be synergistic, indifferent, and antagonistic, respectively (10).

### EtBr accumulation assay

As previously described (10, 35), fluorometric measurement of EtBr (Sigma Aldrich) absorption from *S. aureus* SA-1199B and SA-K-1758 was performed. Briefly, in uptake buffer (7-mM KCl, 110-mM NaCl, 0.4-mM Na2HPO4, 50-mM NH_4_Cl, 52-mM Tris base, and 0.2% glucose, adjusted to pH 7.5 with HCl), bacterial suspensions grown to mid-exponential phase in Mueller-Hinton broth (MHB) were diluted to an OD_600nm_ of 0.3. The bacterial suspensions were treated with 4-µg/mL EtBr in the presence of 1/4×, 1/8×, and 1/16× MIC of indole derivatives and reserpine (Sigma Aldrich), a recognized efflux pump inhibitor. In a Synergy microplate reader (BioTek, USA), the fluorescence gain was recorded after 20 min at λ_excitation_/λ_emission_ of 530 and 600 nm. The equation calculated the relative final fluorescence:

RFF = (RF_treated_ – RF_untreated_) / RF_untreated_,

where RF_treated_ represents the fluorescence in the presence of indole derivatives or reserpine at the last time point (20 min), and RF_untreated_ signifies the fluorescence in the control well (without EPI) at the last time point (20 min) (without EPI and indole derivatives).

When the activity index was greater than 0, the EtBr accumulated more in the presence of the EPI than in the absence of the EPI (untreated cells were considered 0). Negative RFF indicated that less EtBr was accumulated than in the untreated control. RFF greater than 1 in the presence of efflux inhibitors revealed increased EtBr accumulation in the cells (49).

### Measurement of norfloxacin accumulation

The norfloxacin accumulation in the *norA*-over-expressed strain *S. aureus* SA-1199B was measured by fluorescence assay (38, 50). The culture was grown to the mid-log phase. The bacterial suspensions of OD_600nm_ of 0.7 were treated with 16-µg/mL (1/4× MIC) norfloxacin, 1/4× MIC of indole derivatives and reserpine, and were incubated on ice for 5 min. The cells were resuspended in 1-mL glycine buffer (glycine = 0.1 M, HCl = 0.02 M, pH 3) after rinsing twice with phosphate buffer (Na_2_HPO_4_·7H_2_O = 0.0754M, NaH_2_PO_4_·H_2_O = 0.0246 M, pH 7.4). The samples were centrifuged after incubating for 20 min at room temperature, and 1:10 dilution of the supernatant was used to record the fluorescence at λ_excitation_ and λ_emission_ of 281 and 440 nm, respectively.

### Efflux inhibition study using ethidium bromide

The impact of indole derivatives on EtBr efflux in *S. aureus* SA-1199B was studied using fluorometry (10, 35). With shaking, *S. aureus* SA-1199B was grown to an OD_600nm_ of 0.7 at 37°C. The bacterial suspension was centrifuged, washed thrice, and resuspended in PBS (pH 7.4) (Lonza). Furthermore, in sub-inhibitory concentrations of indole derivatives, the bacterial suspensions were treated with 4-µg/mL (1/4 × MIC) EtBr. Allowing the highest amount of EtBr to accumulate, the suspensions were incubated for 60 min at 25°C. The suspensions were centrifuged to stop the reaction and remove excess EtBr at 4°C. The cells were then resuspended in ice-cold PBS, and fluorescence was measured at 530/600 nm at 37°C for 45 min at 9-min intervals. The experiment was carried out in a Synergy microplate reader.

### Membrane potentiation assay

*S. aureus* SA-1199B was grown at 37°C to mid-exponential phase. The cells were centrifuged, washed thrice at 4000 × *g* for 10 min, and resuspended in 50-mM 4-(2-hydroxyethyl)-1-piperazineethanesulfonic acid (HEPES) buffer and 0.1% glucose to achieve an OD_600_ of 0.1. DiSC_3_(5) (Sigma Aldrich) dye at 1-µM concentration and 300-mM KCl (to countervail the outer and cytoplasmic K^+^ concentrations) in HEPES buffer was added to the cells and incubated in the dark at room temperature for 10 min for the probe fluorescence to stabilize (51 – 54). The indole derivatives were added at their MIC to the cells, and valinomycin (Sigma Aldrich) (MIC = 16 µg/mL) was used as the positive control. Bacterial OD_600nm_ was then normalized for all the treatment groups and control. Then, the fluorescence leakage was measured immediately for 54 min at an interval of 9 min at a λ_excitation_/λ_emission_ of 622/670 nm.

### Membrane permeability assay

The membrane permeabilization assay measured the fluorescence of propidium iodide penetrating the membrane according to the Invitrogen Propidium Iodide kit (Thermo Fisher Scientific, Inc.) protocol with certain modifications. In CA-MHB, *S. aureus* SA-1199B was incubated and allowed to grow up to the mid-exponential stage with an OD_600nm_ of 0.3. The 500-µL bacterial suspension was treated with indole derivatives and paenibacillin at 1× MIC for 30 min. After treatment, bacterial cell OD_600nm_ was normalized, and then the culture was washed thrice with normal saline (0.85% NaCl) and suspended in PBS. The suspensions were treated with 30-µM PI and incubated at room temperature for 15–20 min. Any unbound PI was eliminated by centrifuging the suspensions at 13,000 × *g* for 5 min and then suspending in 500-µL PBS. Suspensions of 100 µL were dispensed in a 96-well plate, and fluorescence was measured at a λ_excitation_/ λ_emission_ of 490 and 635 nm, respectively, after 30 min.

### Determination of intracellular ATP levels

An ATP determination kit was used to measure the intracellular ATP levels of bacteria according to the manufacturer’s protocol (Invitrogen, Life Technologies, USA). *S. aureus* SA-1199B bacterial suspension of 500 µL was treated with indole derivatives separately at a sub-inhibitory concentration of 1/4× MIC and CCCP (Sigma Aldrich) at 0.5 µg/mL in MHB for 4 h at 37°C and 200 rpm. After treatment, bacterial cell OD_600nm_ was normalized, and then the cells were centrifuged and resuspended in 100 µL of PBS after incubation. Finally, the cells were sonicated for 15 min, followed by 10 min of alternate heat and cold shock for lyses of the cells. The supernatant was used to quantify total ATP represented as relative luminescence (34).

### Mammalian Ca^2+^ channel blocking assay

According to the manufacturer’s instructions, the Fluo-4 Direct Ca^2+^ channel test kit was used to examine the effect of SMJ-5 on human calcium channels (Life Technologies, Carlsbad, CA, USA) (23). HEK-293T cells (5 × 10^4^) were treated with Probenecid (5 mM) and Fluo-4 dye for 1 h, and the amount of fluorescence was measured. Then the cells were treated with indole derivatives at 1/4× MIC, DMSO (vehicle control), and verapamil at 50 µg/mL. Carbachol (50 µg/mL), the Ca^2+^ channel stimulator, was added after 2 min, and the fluorescence was recorded at 494/516 nm using a microplate reader.

### Hemolysis assay

Fresh rabbit blood erythrocytes were used in the hemolysis assay (55). Blood was collected in a heparin tube and then centrifuged and rinsed with PBS thrice. PBS was used to resuspend the RBCs at the final concentration of 4% (vol/vol). In 180 µL of freshly prepared 4% RBC, 20 µL of indole derivatives at a final concentration of 64, 128, 250, and 500 µg/mL, 0.1% Triton-X (positive control), DMSO (vehicle control) was added. DMSO was subtracted from the readings. The suspension was incubated for 1 h at 37°C before centrifuging for 5 min at 4°C at 1000 × *g*. The supernatant (100 µL) was shifted to a 96-well flat-bottom plate, and absorbance was measured at 570 nm (BioTek, US).

Ac = (Absorbance of control – Absorbance of blank)

### Mammalian cytotoxicity

The cytotoxicity of indole derivatives was investigated on human peripheral blood mononuclear cells (PBMC) and Human Embryonic Kidney 293T cells (HEK) (55). Approximately 20,000 PBMC and 7000 HEK cells were seeded separately per well in separate plates, and they were incubated in a CO_2_ incubator with 5% CO_2_ at 37°C for 24 h. Indole derivatives (250 and 500 µg/mL) were added and incubated at 37°C in a CO_2_ incubator. After 24 h of incubation, MTT (500 µg/mL) was added to the wells after discarding the medium. The formazan crystals were solubilized with a 100-µL stopping solution containing 40% (vol/vol) dimethylformamide (DMF) in 2% (vol/vol) glacial acetic acid and 16% (wt/vol) sodium dodecyl sulfate (SDS) at 4.7 pH. The absorbance was assessed at 570 nm (BioTek, USA).

The given equation was used to calculate the percent viability:

% Cell viability = A_t_/ _Ac_;

A_t_ = (absorbance of test compound – absorbance of blank);

A_c_ = (absorbance of control – absorbance of blank)

### Crystal violet biofilm inhibition

For biofilm formation, *S. aureus* SA-1199B was grown overnight in the Soyabean Casein Digest Medium (Tryptone Soya Broth) (HIMEDIA) with 2.5% glucose. The culture was then diluted to 1 × 10^9^ CFU/mL. Microtiter plates containing indole derivatives and ciprofloxacin combination as detailed for the synergy studies. The wells were washed with PBS twice after 48-h incubation at 37°C to remove the planktonic cells, followed by fixation with methanol for 15 min and air-drying at 37°C in an inverted position for 10 min. The cells in the dried wells were stained with 0.05% (wt/vol) crystal violet (CV) and incubated for 10 min at room temperature. The cells were rinsed in PBS until the control wells holding media only were colorless. 200 µL of ethanol: acetone (80:20) mixture was added to the wells and incubated at room temperature for 10 min. A Synergy H.T. multi-mode microplate reader measured absorbance at 570 nm (BioTek) (56). Compared to the control wells, a reduction of ≥50% absorbance was defined as Minimal biofilm inhibitory concentration (MBIC_50_).

### Crystal violet biofilm eradication

The first step was to generate biofilms, *S. aureus* SA-1199B was grown overnight in the Soyabean Casein Digest Medium (Tryptone Soya Broth) (HIMEDIA) with 2.5% glucose. The culture was then diluted (1 × 10^9^ CFU/mL). The plates were incubated for 48 h. Indole derivatives and ciprofloxacin combinations, as detailed for the synergy studies, were introduced simultaneously to eradicate the pre-formed biofilm at the maturation stage (48-h biofilms) with 200 µL of TSB. Untreated biofilms were employed as control. The plates were incubated at 37°C for 24. The non-adherent cells were removed from each well the next day, and the adhering biofilm was washed twice with PBS, followed by fixation with methanol for 15 min and air-drying at 37°C in an inverted position for 10 min. CV was used to assess the biofilm, and biofilm eradication was estimated as specified. The cells in the dried wells were stained with 0.05% (wt/vol) CV and incubated for 10 min at room temperature. The cells were rinsed in PBS until the control wells holding the medium were colorless. Ethanol:acetone (80:20) mixture of 200 µL was added to the wells and incubated at room temperature for 10 min. A Synergy H.T. multi-mode microplate reader measured absorbance at 570 nm (BioTek) (56). Compared to the control wells, a ≥50% absorbance reduction was defined as minimal biofilm eradication concentration (MBEC_50_).

### Identifying viable cell populations in biofilms

We also used the MTT test to count the number of live cells in the biofilm. The experiment was carried out exactly as stated before, but instead of a CV, MTT (1-mg/mL final concentration) was employed. MTT was added after cell washing, and plates were incubated at 37°C for 4 h. The MTT was solubilized in a solubilizing solution. The formazan crystals were solubilized with a 100-µL stopping solution containing 40% (vol/vol) DMF in 2% (vol/vol) glacial acetic acid and 16% (wt/vol) SDS at 4.7 pH. The absorbance was assessed at 570 nm (BioTek, USA). The findings are shown as MBEC_50_ of live adhering bacteria in the biofilm compared to the control (untreated biofilm) (57).

### Confocal microscopy

SYTO9 dye (Invitrogen, Life Technologies) was used to stain nucleic acid of the cells in biofilms and visualized using confocal laser scanning microscopy (CLSM). CLSM was used to confirm the impact of ciprofloxacin (4 and 16 µg/mL), SMJ-5 (32 µg/mL), and a combination of ciprofloxacin (1 µg/mL), and SMJ-5 (32 µg/mL) was used at MBEC_50_ to visualize the effect. *S. aureus* SA-1199B biofilm was grown on sterile 18-mm glass coverslips coated with poly-L-lysine placed in a 12-well polystyrene plate (Falcon) (58). Ciprofloxacin was utilized at 4 and 16 µg/mL; SMJ-5 was used at 32 µg/mL; and a combination of ciprofloxacin at 1 µg/mL and SMJ-5 at 32 µg/mL was used at MBEC_50_ to visualize the effect. The plates were incubated at 37°C for 24 h. The plates were washed using normal saline to remove planktonic cells. SYTO9 was diluted 1000 times in PBS, poured into the biofilm wells, and left at room temperature for 20–30 min. After incubation, tfhree more saline washes were given, and on an oil immersion lens (×40), images were visualized using Nikon A1 plus Ti confocal microscope with a Nikon A1R scan head. Images were captured using NIS elements software.

### Staphyloxanthin extraction and spectrometric measurement of its biosynthetic intermediates

In CA-MHB, *S. aureus* ATCC 29213 was incubated and grown overnight. The culture was then diluted 1,000×, and 5-mL culture was treated with indole derivatives at a sub-inhibitory concentration (1/4× MIC) for 48 h. The treated and untreated bacterial cells’ optical densities (OD) were then normalized and centrifuged at 8,000 rpm for 10 min to extract carotenoid pigments. PBS was used to rinse the cell pellets before they were suspended in 500 µL of methanol. The tubes were shaken for 5 h at 400 rpm and 55°C while covered in aluminum foil. The extracted pigments were then separated from the supernatant by centrifuging the tubes. Using a plate reader and various wavelengths, the staphyloxanthin intermediates present in the collected supernatant was quantified. The wavelengths used for the absorbance measurements were 286 nm for 4,4′-diapophytoene, 435 nm for 4,4′-diaponeurosporene, 455 nm for 4,4′-diaponeurosporenic acid, and 462 nm for staphyloxanthin (59). As a negative control, methanol was employed.

### Post-antibiotic assay

In CA-MHB, *S. aureus* SA-1199B was grown to the mid-log phase. The culture was then adjusted to an OD_600nm_ of 0.25. Further, the bacterial culture was diluted a hundred times, and 1-mL bacterial culture was treated with ciprofloxacin at 1×, 1/2×, and 1/4× MIC alone and in combination with SMJ-3, SMJ-5, SMJ-9, and SMJ-10 (1/4× MIC) at 37°C for 2 h. After that, bacterial suspension OD_600nm_ was normalized for all the treatment groups and control. Then, the bacterial suspensions were centrifuged for 10 min at 1200 × *g* and washed thrice in new media to eliminate the drug’s carry-over effect. The untreated bacterial suspension was included as the control. A 300-µL bacterial suspension was added to the wells of a transparent bottom 96-well flat-bottom plate in triplicate and incubated at 37°C for 18 h. PAE was determined using the formula PAE = *T*_50_ − *C*_50_, where *T*_50_ and *C*_50_ refer to the time taken by the treated and untreated cultures to reach a 50% OD of the final absorbance achieved by the control, respectively (60).

### MPC

*S. aureus* SA-1199B (10^10^ CFU) bacterial suspensions were spread on MHA plates with varying concentrations of ciprofloxacin alone and in conjunction with SMJ-5 at 32 µg/L and were incubated at 37°C. After 48 h, the colonies were enumerated, and the mutation frequency was estimated by dividing the number of survivors by the number of CFU plated. The MPC was considered to be the concentration at which no colony appeared (61).

### Time-kill kinetics

The *S. aureus* SA-1199B was grown to the mid-exponential phase, and a suspension (OD_600nm_ ~0.3) was prepared. The initial inoculum of the *S. aureus* SA-1199B was challenged with ciprofloxacin at 8 and 2 µg/mL separately. Likewise, SMJ-5 was tested at its sub-inhibitory concentration of 32 µg/mL both in the presence and absence of 2 µg/mL of ciprofloxacin. At different time points (0, 2, 4, 6, 8, 12, and 24 h), the culture was assessed for the bacterial count and expressed as CFU per milliliter (62).

### Persisters assay

*S. aureus* ATCC 29213 was grown for 24 h to investigate the antibiotic-induced persister cell production. These stationary-phase cells were treated with 10 µg/mL (20× MIC) of ciprofloxacin for 3 h. Aliquots were withdrawn at intervals of 60 min for 3 h to determine the number of viable bacteria that survived ciprofloxacin exposure. DMSO was used as vehicle control. The viable colonies were determined after 24 h of incubation and represented as log_10_ CFU/mL. After incubation, the remaining cells were pelleted, washed, and allowed to flow in a fresh medium overnight. And the process was repeated for 3 days to check the heritability of persister formation. On the fourth day, the cells were treated with SMJ-5 (32 µg/mL) and ciprofloxacin (10 µg/mL), and aliquots were withdrawn every 60 min for 3 h to determine the viable count (63).

### Extraction of total RNA and semi-quantitative RT-PCR

*S. aureus* SA-1199B was grown overnight. The culture was diluted 1,000× and then treated with SMJ-5 (32 µg/mL), ciprofloxacin (2 µg/mL), and SMJ-5 (32 µg/mL) + ciprofloxacin (0.25 µg/mL) for 16 h. The culture was centrifuged at 10,000 × *g* and treated with 5 M guanidine thiocyanate for 5 min, then again centrifuged, pelleted, and stored at −80°C until RNA isolation. The pellet was then washed with Diethyl pyrocarbonate (DEPC) water and resuspended in 1-mL TRIzol reagent (Thermo Fisher Scientific, Inc.), and then lysis was performed using a bead beater for 30 s at an interval of 3–5 min thrice at a speed of 6 rpm. The suspension was placed on ice instantly after every cycle. Total RNA was purified from the bacterial suspension using the Qiagen RNeasy Mini kit following the manufacturer’s protocol.

The reverse transcription PCR was conducted using the SuperScript III Platinum One-Step qRT-PCR Kit (Thermo Fisher Scientific, Inc.) to synthesis cDNA following the manufacturer’s protocol. The following were the PCR thermocycling parameters: 50°C for 14.02 min, 95°C for 2 min, 40 cycles of 95°C for 15 s, 60°C for 30 s, and a melt curve of 95°C for 15 s, and 60°C for 1 min. Relative gene expression was determined using the power 2^−ΔΔCT^ technique. The housekeeping gene 16s rRNA was used as an endogenous control to normalize the expression levels of target genes. Each sample was analyzed in duplicate, and all samples were examined parallel for their expression. The fold induction was calculated using the average replicate values. In *norA* gene expression analysis in clinical strains, the *S. aureus* ATCC 29213 strain was used as a reference sample strain. The primer sequences for the *norA* (28) and 16s rRNA genes are available in the supplemental materials (Table S10).

### Macrophage invasion assay

The THP-1 macrophage cell line was used to study *S. aureus* intracellular invasion in the presence of SMJ-5 (35). *S. aureus* SA-1199B, SA-1199, and K-1758 (10^6^ CFU/well) were employed to infect macrophages (10^5^ cells/well with or without SMJ-5 (32 µg/mL) and were incubated for 2 h. After washing out the extracellular bacteria three times with PBS, the cells were treated for 30 min with gentamicin (50 µg/mL). The cells were treated with 0.1% saponin for a short interval, and the viability of intracellular bacteria was calculated by plating on MHA.

### Acute toxicity, blood-biochemical, and histopathological studies in animal model

This experiment was performed in two stages (30). The outcome from each stage determined the next step (i.e., whether to halt or continue to the next level). Stage 1 consisted of three groups, and stage 2 consisted of two groups; each group contained three mice each. SMJ-5 was administered sub-cutaneously at a dose of 50 mg/kg (stage 1, group 1), 100 mg/kg (stage 1, group 2), and 250 mg/kg (stage 1, group 3) and in stage 2 and at a dose of 500 mg/kg (stage 2, group 1) and 1,000 mg/kg (stage 2; group 2) (Table S8). Animals were observed every 2 h after administration for 12 h and then every 12 h for a period of 7 days. Mortality and behavioral toxicity signs were recorded.

Mice were given an intraperitoneal injection of ketamine (90 mg/kg) and xylazine (10 mg/kg) (64) at the end of 7 days. After that, a blood drop was collected from the tail vein of the anesthetized mouse to check the blood glucose level. Blood (400–500 µL) was collected through the retro-orbital route from mice.

The blood in MCT was left undisturbed for 30 min at room temperature and then centrifuged for 10 min at 1,000 × *g*. After that, the serum was collected and refrigerated at −80°C. The mice were dissected, and six organs, namely, the lung, heart, brain, liver, kidney, and spleen, were removed and stored in 10% neutral buffered formalin. The blood-biochemical and histopathological studies were outsourced from a private medicos center.

### *In vivo* thigh infection model

Female BALB/c mice received two intraperitoneal doses of 150 and 100 mg/kg of cyclophosphamide for 4 days to introduce neutropenia. The mice were after that injected intramuscularly with 50 µL of *S. aureus* SA-1199B 3.2 × 10^7^ CFU/mL). Four hours post-infection, a single sub-cutaneous dose of 10 mg/kg of SMJ-5 and 10 mg/kg of ciprofloxacin (Sigma Aldrich) alone and in combination was administered in mice. The fourth group was administered 50 mg/kg of ciprofloxacin alone to check if the combination was better than the high-dose ciprofloxacin. One group of infected mice was euthanized pre-treatment by cervical dislocation after 4 h of infection. The right thighs were removed and homogenized, and CFUs were determined. The other four groups of mice were euthanized by cervical dislocation 20 h after injecting the administered dose of SMJ-5 and ciprofloxacin. All four groups’ CFU was enumerated from the right thigh (65).

### Mouse peritonitis model

The experiment was carried out as stated previously, with a few changes (66). The mice were given 500 µL of 2 × 10^9^ CFU/mL *S. aureus* SA-1199B suspension prepared in 5% mucin intraperitoneally. All groups (*n* = 10) were sub-cutaneously administered at 1, 3, and 5 h after infection with 10 mg/kg of SMJ-5 and 10 mg/kg of ciprofloxacin alone and in combination. The survival condition and weight variations of mice were monitored for 7 days. The mice that survived until the seventh day were euthanized by cervical dislocation.

### Statistical analyses

Most trials used biological replicates, and consistent outcomes were attained each time. The information is shown as mean and standard deviation. Different mean *t*-tests (two-tailed) were employed to find group differences. *P* values of 0.05 (*) were regarded as statistically significant, and 0.01 (**), 0.001 (***), and 0.0001 (***) were considered highly significant.

## AFTER EPUB

*[This article was published on 27 September 2023 without the supplemental material. The supplemental material was included in the current version, posted on 17 October 2023.]*

## SUPPLEMENTAL MATERIAL

Supplemental material spectrum.04876-22-s0001.docx Tables S.1 to S.10 and Fig. S.1 to S.5.

ASM does not own the copyrights to Supplemental Material that may be linked to, or accessed through, an article. The authors have granted ASM a non-exclusive, world-wide license to publish the Supplemental Material files. Please contact the corresponding author directly for reuse.
