# Supplementary material for: Efflux pump inhibitory potential of indole derivatives as an arsenal against norA over-expressing Staphylococcus aureus
Source: Microbiol Spectr. 2023 Sep 27;11(5):e04876-22. doi: 10.1128/spectrum.04876-22 (PMC10581058; doi:10.1128/spectrum.04876-22)
Supplement: Supplemental material — Tables S.1 to S.10 and Fig. S.1 to S.5. [file spectrum.04876-22-s0001.docx]

**Efflux pump inhibitory potential of indole derivatives as an arsenal against *norA* over-expressing *Staphylococcus aureus***

Nishtha Chandal^1,2^, Rushikesh Tambat^1^, Ritu Kalia^3^, Gautam Kumar^4^, Nisha Mahey^1,2^, Sanjay Jachak^#,3^, and Hemraj Nandanwar^1,2*^

^1^Clinical Microbiology & Antimicrobial Research Laboratory, CSIR-Institute of Microbial Technology, Sector 39-A, Chandigarh-160036, India

^2^Academy of Scientific & Innovative Research (AcSIR), Ghaziabad, Uttar Pradesh-201002, India

^3^ Department of Natural Products, National Institute of Pharmaceutical Education and Research Mohali-160062, India

^4^Department of Natural Products, Chemical Sciences, National Institute of Pharmaceutical Education and Research- Hyderabad. Balanagar, 500037, India

***Address for correspondence:**

Dr. Hemraj Nandanwar, Chief Scientist

Council of Scientific & Industrial Research-Institute of Microbial Technology, Sector 39-A, Chandigarh-160036

E-mail ID: [hemraj@imtech.res.in](mailto:hemraj@imtech.res.in)

Telephone: +91-172-2880338 Fax: +91-172-2690585/2690632

**^#^Co-corresponing Author**

Prof. Sanjay Jachak

Department of Natural Products, National Institute of Pharmaceutical Education and Research Mohali-160062, India

E-mail ID: [sjachak11@gmail.com](mailto:sjachak11@gmail.com)

**Running Title:** Indole-based NorA efflux pump inhibitors

**Table S.1.** Scope of the 2-(2’-Aminophenyl) indole [RP2] derivatives synthesis

| S. no. | 4 | 6 | Yield (%) |
| --- | --- | --- | --- |
| 1. |  |  | 82 |
| 2. | **4** |  | 76 |
| 3. | **4** |  | 91 |
| 4. | **4** |  | 54 |
| 5. | **4** |  | 81 |
| 6. | **4** |  | 77 |
| 7. | **4** |  | 82 |
| 8. | **4** |  | 58 |

All tested compounds were tested for the purity check using the Waters HPLC system (equipped with 600E pump, an in4-channelhannel degasser, a 717autosamplerampler, a temperature control module, a 2998 photodiode array (PDA) detector and Empower™2 software database version 6.10 (Waters, USA). All compounds were >95 % pure before any experiments were done.

In some of the spectra, residual solvent peaks were there, which were removed after spectral data were recorded and further used for biological activities.

**Table S.2:** Checkerboard synergy assay of SMJ 1-10 against K1758 (*norA* Deletion strain)

| 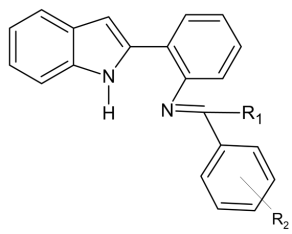 | | | | | | | | | | | | | |
| --- | --- | --- | --- | --- | --- | --- | --- | --- | --- | --- | --- | --- | --- |
| Compound | **R_1_** | | | **R_2_** | **Intrinsic MIC (µg/mL)** | Concentration | **Norfloxacin** | | | **Ciprofloxacin** | | | |
|  |  | |  | |  |  | **MIC (µg/mL)** | **Modulation Factor** | **FICI** | **MIC (µg/mL)** | **Modulation Factor** | | **FICI** |
| SMJ-1 | CH_3_ | -CH_3_ | | | 2 | 0.5 | 0.125 | 2 | 0.75 | 0.0625 | | 2 | 0.75 |
|  |  |  | | |  | 0.25 | 0.25 | - | 1.125 | 0.125 | | - | 1.125 |
|  |  |  | | |  | 0.125 | 0.25 | - | 1.062 | 0.125 | | - | 1.062 |
|  |  |  | | |  | 0 | 0.25 | - | 1 | 0.125 | | - | 1 |
| SMJ-3 | CH_3_ | -OH | | | 8 | 2 | 0.125 | 2 | 0.75 | 0.062 | | 2 | 0.75 |
|  |  |  | | |  | 1 | 0.25 | - | 1.125 | 0.125 | | - | 1.125 |
|  |  |  | | |  | 0.5 | 0.25 | - | 1.062 | 0.125 | | - | 1.062 |
|  |  |  | | |  | 0 | 0.25 | - | 1 | 0.125 | | - | 1 |
| SMJ-5 | CH_3_ | -dihydroxyl | | | 64 | 16 | 0.25 | - | 1.25 | 0.125 | | - | 1.25 |
|  |  |  | | |  | 8 | 0.25 | - | 1.125 | 0.125 | | - | 1.125 |
|  |  |  | | |  | 4 | 0.25 | - | 1.062 | 0.125 | | - | 1.062 |
|  |  |  | | |  | 0 | 0.25 | - | 1 | 0.125 | | - | 1 |
| SMJ-6 | H | -F | | | 4 | 1 | 0.25 | - | 1.25 | 0.125 | | - | 1.25 |
|  |  |  | | |  | 0.5 | 0.25 | - | 1.125 | 0.125 | | - | 1.125 |
|  |  |  | | |  | 0.25 | 0.25 | - | 1.062 | 0.125 | | - | 1.062 |
|  |  |  | | |  | 0 | 0.25 | - | 1 | 0.125 | | - | 1 |
| SMJ-7 | CH_3_ | -NO_2_ | | | 4 | 1 | 0.25 | - | 1.25 | 0.125 | | - | 1.25 |
|  |  |  | | |  | 0.5 | 0.25 | - | 1.125 | 0.125 | | - | 1.125 |
|  |  |  | | |  | 0.25 | 0.25 | - | 1.062 | 0.125 | | - | 1.062 |
|  |  |  | | |  | 0 | 0.25 | - | 1 | 0.125 | | - | 1 |
| SMJ-8 | H | -CF_3_ | | | 16 | 4 | 0.25 | - | 1.25 | 0.125 | | - | 1.25 |
|  |  |  | | |  | 2 | 0.25 | - | 1.125 | 0.125 | | - | 1.125 |
|  |  |  | | |  | 1 | 0.25 | - | 1.062 | 0.125 | | - | 1.062 |
|  |  |  | | |  | 0 | 0.25 | - | 1 | 0.125 | | - | 1 |
| SMJ-9 | CH_3_ | -SCH_3_ | | | 2 | 0.5 | 0.25 | - | 1.25 | 0.125 | | - | 1.25 |
|  |  |  | | |  | 0.25 | 0.25 | - | 1.125 | 0.125 | | - | 1.125 |
|  |  |  | | |  | 0.125 | 0.25 | - | 1.062 | 0.125 | | - | 1.062 |
|  |  |  | | |  | 0 | 0.25 | - | 1 | 0.125 | | - | 1 |
| SMJ-10 | CH_3_ | NH_2_ | | | 64 | 16 | 0.25 | - | 1.25 | 0.125 | | - | 1.25 |
|  |  |  | | |  | 8 | 0.25 | - | 1.125 | 0.125 | | - | 1.125 |
|  |  |  | | |  | 4 | 0.25 | - | 1.062 | 0.125 | | - | 1.062 |
|  |  |  | | |  | 0 | 0.25 | - | 1 | 0.125 | | - | 1 |
| Reserpine |  |  | | | 128 | 32 | 0.0625 | 4 | 0.5 | 0.0312 | | 4 | 0.5 |
|  |  |  | | |  | 16 | 0.25 | - | 1.125 | 0.125 | | - | 1.125 |
|  |  |  | | |  | 8 | 0.25 | - | 1.062 | 0.125 | | - | 1.062 |
|  |  |  | | |  | 0 | 0.25 | - | 1 | 0.125 | | - | 1 |

**Table S.3: Checkerboard synergy assay of SMJ 1-10 against *S. aureus* RN-4220 (MsrA over-expressed)**

| 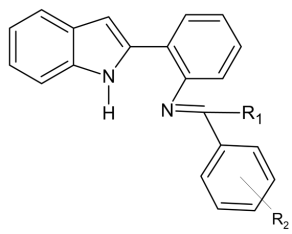 | | | | | | | | | |
| --- | --- | --- | --- | --- | --- | --- | --- | --- | --- |
| Compound | **R_1_** | **R_2_** | | **Intrinsic MIC (µg/ml)** | | **Concentration** | **Erythromycin** | | |
|  |  |  |  |  |  |  | **MIC** | **Modulation Factor** | **FICI** |
| SMJ-1 | CH_3_ | | -CH_3_ | 16 | 4 | | 32 | 4 | 0.5 |
|  |  | |  |  | 2 | | 64 | 2 | 0.625 |
|  |  | |  |  | 1 | | 128 | - | 1.0625 |
|  |  | |  |  | 0 | | 128 | - | 1 |
| SMJ-3 | CH_3_ | | -OH | 32 | 8 | | 64 | 2 | 0.75 |
|  |  | |  |  | 4 | | 128 | - | 1.125 |
|  |  | |  |  | 2 | | 128 | - | 1.0625 |
|  |  | |  |  | 0 | | 128 | - | 1 |
| SMJ-5 | CH_3_ | | -dihydroxyl | 128 | 32 | | 64 | 2 | 0.75 |
|  |  | |  |  | 16 | | 64 | 2 | 0.625 |
|  |  | |  |  | 8 | | 64 | 2 | 0.5625 |
|  |  | |  |  | 0 | | 128 | - | 1 |
| SMJ-6 | H | | -F | 128 | 32 | | 128 | - | 1.25 |
|  |  | |  |  | 16 | | 128 | - | 1.125 |
|  |  | |  |  | 8 | | 128 | - | 1.0625 |
|  |  | |  |  | 0 | | 128 | - | 0 |
| SMJ-7 | CH_3_ | | -NO_2_ | 64 | 16 | | 128 | - | 1.25 |
|  |  | |  |  | 8 | | 128 | - | 1.125 |
|  |  | |  |  | 4 | | 128 | - | 1.0625 |
|  |  | |  |  | 0 | | 128 | - | 1 |
| SMJ-8 | H | | -CF_3_ | 64 | 16 | | 128 | - | 1.25 |
|  |  | |  |  | 8 | | 128 | - | 1.125 |
|  |  | |  |  | 4 | | 128 | - | 1.0625 |
|  |  | |  |  | 0 | | 128 | - | 1 |
| SMJ-9 | CH_3_ | | -SCH_3_ | 64 | 16 | | 128 | - | 1.25 |
|  |  | |  |  | 8 | | 64 | 2 | 0.625 |
|  |  | |  |  | 4 | | 64 | 2 | 0.5625 |
|  |  | |  |  | 0 | | 128 | - | 1 |
| SMJ-10 | CH_3_ | | NH_2_ | 128 | 32 | | 64 | 2 | 0.75 |
|  |  | |  |  | 16 | | 64 | 2 | 0.625 |
|  |  | |  |  | 8 | | 128 | - | 1.0625 |
|  |  | |  |  | 0 | | 128 | - | 1 |
| Reserpine |  | |  | 128 | 32 | | 64 | 2 | 0.75 |
|  |  | |  |  | 16 | | 128 | - | 1.125 |
|  |  | |  |  | 8 | | 128 | - | 1.0625 |
|  |  | |  |  | 0 | | 128 | - | 1 |

**Table S.4.a: Checkerboard synergy assay of indole derivatives against *S. aureus* SA-1199B taking moxifloxacin as the antibiotic**

| 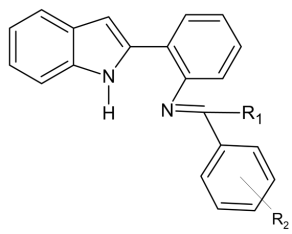 | | | | | | | |
| --- | --- | --- | --- | --- | --- | --- | --- |
| Compound | **R_1_** | **R_2_** | **Intrinsic MIC (µg/ml)** | **Concentration** | **Moxifloxacin** | | |
|  |  |  |  |  | **MIC** | **Modulation Factor** | **FICI** |
| SMJ-1 | CH_3_ | -CH_3_ | 2 | 0.5 | 0.25 | - | 1.25 |
|  |  |  |  | 0.25 | 0.25 | - | 1.125 |
|  |  |  |  | 0.125 | 0.25 | - | 1.0625 |
|  |  |  |  | 0 | 0.25 | - | 1 |
| SMJ-3 | CH_3_ | -OH | 16 | 4 | 0.25 | - | 1.25 |
|  |  |  |  | 2 | 0.25 | - | 1.125 |
|  |  |  |  | 1 | 0.25 | - | 1.0625 |
|  |  |  |  | 0 | 0.25 | - | 1 |
| SMJ-5 | CH_3_ | -dihydroxyl | 128 | 32 | 0.25 | - | 1.25 |
|  |  |  |  | 16 | 0.25 | - | 1.125 |
|  |  |  |  | 8 | 0.25 | - | 1.0625 |
|  |  |  |  | 0 | 0.25 | - | 1 |
| SMJ-6 | H | -F | 4 | 1 | 0.25 | - | 1.25 |
|  |  |  |  | 0.5 | 0.25 | - | 1.125 |
|  |  |  |  | 0.25 | 0.25 | - | 1.0625 |
|  |  |  |  | 0 | 0.25 | - | 1 |
| SMJ-7 | CH_3_ | -NO_2_ | 128 | 32 | 0.25 | - | 1.25 |
|  |  |  |  | 16 | 0.25 | - | 1.125 |
|  |  |  |  | 8 | 0.25 | - | 1.0625 |
|  |  |  |  | 0 | 0.25 | - | 1 |
| SMJ-8 | H | -CF_3_ | 64 | 16 | 0.25 | - | 1.25 |
|  |  |  |  | 8 | 0.25 | - | 1.125 |
|  |  |  |  | 4 | 0.25 | - | 1.0625 |
|  |  |  |  | 0 | 0.25 | - | 1 |
| SMJ-9 | CH_3_ | -SCH_3_ | 16 | 4 | 0.125 | 2 | 0.75 |
|  |  |  |  | 2 | 0.25 | - | 1.125 |
|  |  |  |  | 1 | 0.25 | - | 1.0625 |
|  |  |  |  | 0 | 0.25 | - | 1 |
| SMJ-10 | CH_3_ | NH_2_ | 128 | 32 | 0.25 | - | 1.25 |
|  |  |  |  | 16 | 0.25 | - | 1.125 |
|  |  |  |  | 8 | 0.25 | - | 1.0625 |
|  |  |  |  | 0 | 0.25 | - | 1 |

**Table S.4.b: MIC of various antibiotics on *S. aureus* SA-1199B, SA-1199, and K1758 strains**

|  | ***S. aureus* SA-1199B** | ***S. aureus* SA-1199** | ***S. aureus* K1758** |
| --- | --- | --- | --- |
|  | **MIC (µg/mL)** | | |
| **Ciprofloxacin** | 8 | 0.25 | 0.125 |
| **Norfloxacin** | 64 | 0.5 | 0.25 |
| **Levofloxacin** | 0.25 | 0.125 | 0.25 |
| **Moxifloxacin** | 0.25 | 0.125 | 0.125 |
| **Tetracycline** | 1 | 1 | 1 |

**Table S.5:** Combination effect of Ciprofloxacin and SMJ-5 at 1/4 × MIC on clinical strains

| Strain | MIC^a^ | MIC^b^ | MIC^c^ |
| --- | --- | --- | --- |
| MRSA-1 | 16 | 128 | 1 |
| MRSA-2 | 16 | 128 | 8 |
| MRSA-3 | 16 | 128 | 0.25 |
| MRSA-4 | 128 | 128 | 64 |
| GMCH *S. aureus* 6188 | 16 | >128 | 0.5 |
| GMCH *S. aureus* 6005 | 32 | 128 | 8 |
| GMCH *S. aureus* 5712 | 32 | 128 | 8 |
| GMCH *S. aureus* 6187 | 16 | 128 | 4 |
| GMCH *S. aureus* 5898 | 16 | 128 | 4 |
| GMCH *S. aureus* 5946 | 64 | 128 | 8 |
| GMCH *S. aureus* 5690 | 8 | >128 | 2 |
| GMCH *S. aureus* 6004 | 8 | 128 | 4 |
| GMCH *S. aureus* 3939 | 8 | 128 | 2 |
| GMCH *S. aureus* 5657 | 8 | 128 | 2 |
| GMCH *S. aureus* 3934 | 8 | 128 | 2 |
| GMCH *S. aureus* 3691 | 8 | 128 | 2 |
| GMCH *S. aureus* 6011 | 8 | 128 | 1 |

**MIC^a^represents MIC of ciprofloxacin alone; MIC^b^ represents MIC of SMJ-5 alone; MIC^c^ represents MIC of ciprofloxacin with SMJ-5 at 32 µg/mL concentration.**

**Table S.6:** Combination effect of erythromycin and moxifloxacin with SMJ-5 at 1/4 × MIC on clinical strains (MRSA-1, MRSA-3, GMCH *S. aureus* 6188, and GMCH *S. aureus* 5946)

|  | Erythromycin (µg/mL) | | | Moxifloxacin (µg/mL) | | |
| --- | --- | --- | --- | --- | --- | --- |
| Strain | MIC^a^ | MIC^b^ | MIC^c^ | MIC^a^ | MIC^b^ | MIC^c^ |
|  |  |  |  |  |  |  |
| MRSA-1 | >500 | 128 | >500 | 4 | 128 | 4 |
| MRSA-3 | >500 | 128 | >500 | 0.5 | 128 | 0.5 |
| GMCH *S. aureus* 6188 | >500 | 128 | >500 | 0.5 | 128 | 0.25 |
| GMCH *S. aureus* 5946 | >500 | 128 | >500 | 0.25 | 128 | 0.25 |

**MIC^a^represents MIC of antibiotic alone; MIC^b^ represents MIC of SMJ-5 alone; MIC^c^ represents MIC of antibiotic with SMJ-5 at 32 µg/mL concentration.**

**Table S.7.a:** Inhibition of biofilm biomass was determined by using crystal violet assay, and eradication of biofilm biomass and viable bacterial cells in the mature biofilm was determined by using crystal violet and MTT assay using *S. aureus* SA1199B

| Compounds | MBIC_50_ of ciprofloxacin (µg/mL) | MBEC_50_ of ciprofloxacin (µg/mL) | MBEC_50_ of ciprofloxacin (µg/mL) |
| --- | --- | --- | --- |
|  | **Crystal violet assay** | | **MTT assay** |
| Ciprofloxacin alone | 8 | 16 | 8 |
| SMJ-1 (0.5 µg/mL) | 4 | 8 | 4 |
| SMJ-3 (4 µg/mL) | 2 | 4 | 2 |
| SMJ-5 (32 µg/mL) | 0.5 | 1 | 1 |
| SMJ-6 (1 µg/mL) | 4 | 8 | 4 |
| SMJ-7 (32 µg/mL) | 1 | 4 | 1 |
| SMJ-8 (16 µg/mL) | 4 | 16 | 4 |
| SMJ-9 (4 µg/mL) | 1 | 2 | 4 |
| SMJ-10 (32 µg/mL) | 2 | 4 | 1 |

**Table S.7.b:** Checkerboard synergy assay of SMJ-5 with ciprofloxacin against *S. aureus* SA1199 and inhibition of biofilm biomass was determined by using crystal violet assay, and eradication of biofilm biomass and viable bacterial cells in the mature biofilm was determined by using crystal violet and MTT assay using *S. aureus* SA1199

| Checkerboard synergy assay of SMJ-5 with ciprofloxacin against *S. aureus* SA1199 | | | | | |
| --- | --- | --- | --- | --- | --- |
| Compound | **Intrinsic MIC (µg/mL)** | Concentration | Cipro**floxacin** | | |
|  |  |  | **MIC (µg/mL)** | **Modulation Factor** | **FICI** |
| SMJ-5 | 128 | 32 | 0.0625 | 4 | 0.5 |
|  |  | 16 | 0.125 | 2 | 0.625 |
|  |  | 8 | 0.25 | - | 1.0625 |
|  |  | 0 | 0.25 | - | 1 |
|  |  |  |  |  |  |
| Biofilm assays | | | | | |

| **Compound** | **MBIC_50_ of ciprofloxacin (µg/mL)** | **MBEC_50_ of ciprofloxacin (µg/mL)** | | | **MBEC_50_ of ciprofloxacin (µg/mL)** | |
| --- | --- | --- | --- | --- | --- | --- |
|  | **Crystal violet assay** | | | **MTT assay** | |  |
| **Ciprofloxacin alone** | 0.25 | | 0.5 | 0.5 | | |
| **SMJ-5 (32 µg/mL)** | 0.125 | | 0.25 | 0.25 | | |

**Table S.8:** Acute toxicity doses at various stages

| Stage | Doses in mg/Kg | | |
| --- | --- | --- | --- |
|  | **Group 1** | **Group 2** | **Group 3** |
| 1 | 50 | 100 | 250 |
| 2 | 500 | 1000 |  |

**Table S.9:** Biochemical test from blood serum of drug-treated mice at various concentrations

| **Stage 2** | | | |
| --- | --- | --- | --- |
| **Investigation** | **Group 2 (1000 mg/Kg)** | **Group 1**  **(500 mg/Kg)** | **Control** |
| **Body weight (Before dosing)** | 15.073 ± 1.127 | 16.136 ± 0.470 | 16.306 ± 1.277 |
| **Body weight (After dosing)** | 14.486 ± 0.776 | 16.46 ± 0.850 | 16.603 ± 1.291 |
| **Blood Glucose (After dosing)** | 90 | 79 | 83 |
|  | | | |
| **Renal Function test complete (Kidney Panel)** | | | |
| **Calcium** | 9.3 ± 0.424 | 9.45 ± 0.212 | 9.35 ± 0.070 |
| **Blood urea** | 38 ± 5.656 | 36 ± 0.0 | 33 ± 1.414 |
| **Serum Creatinine** | 0.295 ± 0.021 | 0.28 ± 0.028 | 0.255 ± 0.021 |
| **Alkaline Phosphatase (ALP)** | 92.5 ± 79.903 | 135 ± 36.769 | 110.5 ± 54.447 |
| **Phosphorus** | 9.35 ± 1.202 | 11.25 ± 2.05 | 10.05 ± 0.353 |
| **Uric acid** | 2.7 ± 0.282 | 3.5 ± 1.272 | 2.1 ± 0.848 |
|  | | | |
| **Liver Function Test with GGTP** | | | |
| **Bilirubin (Total)** | 0.215 ± 0.021 | 0.195 ± 0.035 | 0.245 ± 0.021 |
| **Bilirubin; Direct (Conjugated)** | 0.105 ± 0.007 | 0.075 ± 0.035 | 0.105 ± 0.007 |
| **Bilirubin; Indirect (Unconjugated)** | 0.11 ± 0.014 | 0.12 ± 0.0 | 0.14 ± 0.028 |
| **SGOT (AST)** | 134 ± 50.911 | 305.5 ± 258.093 | 109 ± 36.769 |
| **SGPT (ALT)** | 34 ± 8.485 | 75 ± 50.911 | 36.5 ± 10.606 |
| **Alkaline Phosphatase** | 92.5 ± 79.903 | 135 ± 36.769 | 110.5 ± 54.447 |
| **Total Protein** | 5.65 ± 1.060 | 5.65 ± 0.494 | 5.3 ± 0.989 |
| **Albumins** | 2.7 ± 0.141 | 2.85 ± 0.070 | 2.7 ± 0.0 |
| **Globulin** | 2.95 ± 1.202 | 2.8 ± 0.565 | 2.6 ± 0.989 |
| **A/G Ratio** | 1 ± 0.424 | 1.05 ± 0.212 | 1.1 ± 0.424 |
| **Triglycerides** | 129 ± 16.970 | 143 ± 9.899 | 92.5 ± 4.949 |
| **Total Cholesterol** | 106.5 ± 16.263 | 111 ± 2.828 | 100 ± 1.414 |
|  |  |  |  |

**Table S.10: Primers used for** ***norA*, *mgrA* and 16s rRNA.**

| **Gene** | **Primer** | **Sequence (5'‐3')** |
| --- | --- | --- |
| *norA* | Sense | GTTGCTGCTTTCGCCTTATCTC |
| *norA* | Antisense | GGCATAACCATACCAGCACTCA |
| *mgrA* | Sense | TGCTCAAAGACAAGTTAATCGCT |
| *mgrA* | Antisense | CTGTACCAGTATCGAGTGCTAA |
| 16s | Sense | CGAAGAACCTTACCAAATCTTGACA |
| 16s | Antisense | AATGATGGCAACTAAGCTTAAGGG |

**rRNA, ribosomal RNA**

**Supplementary figure**

**RP2**

**
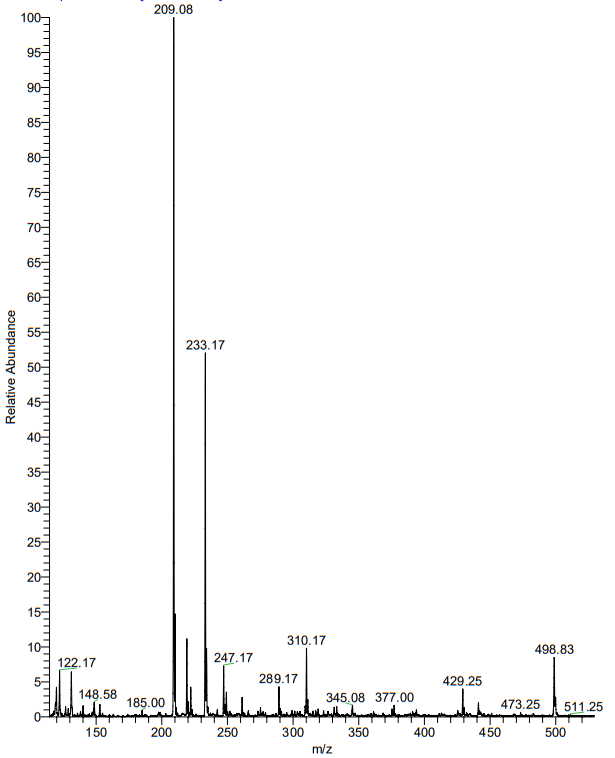
**

**
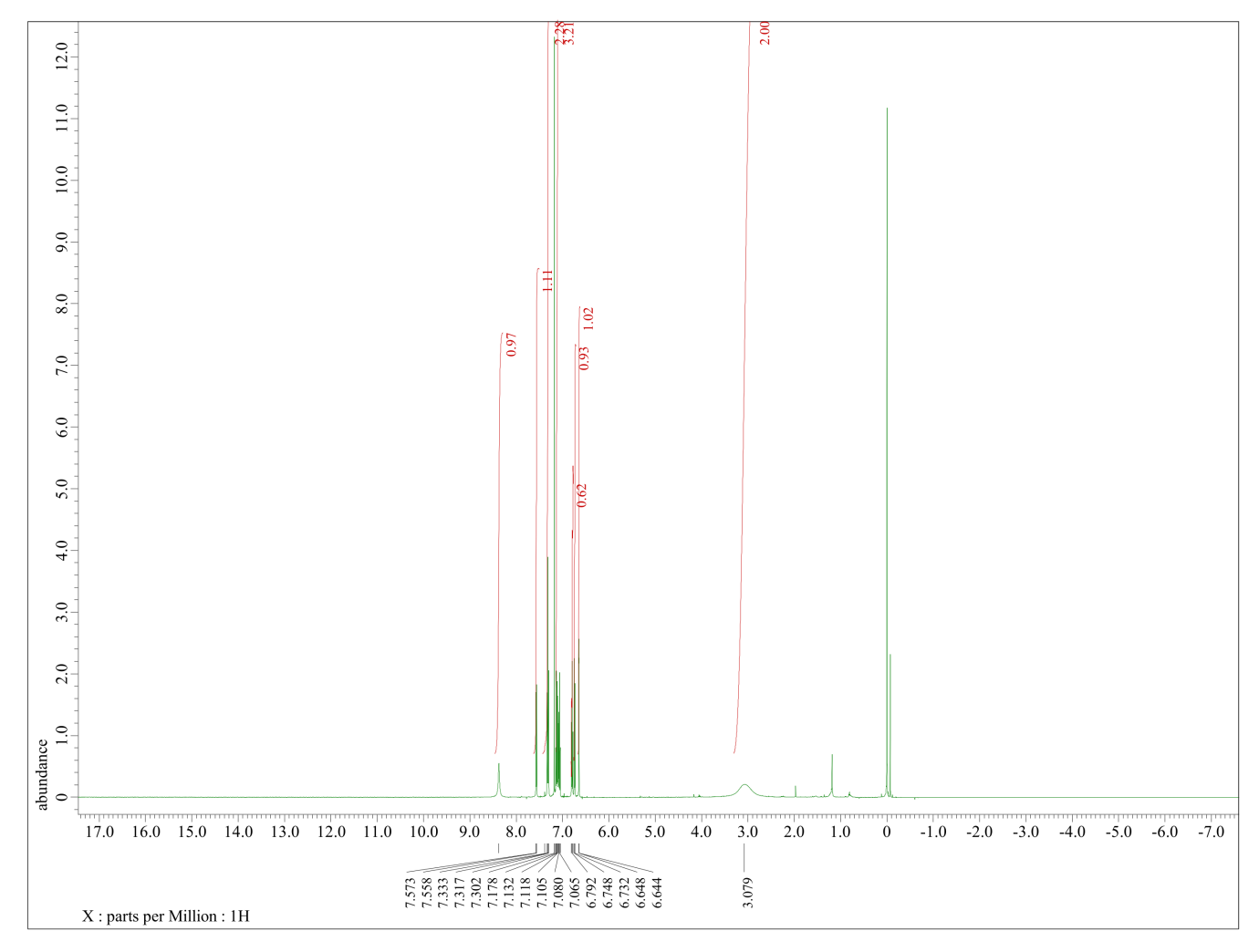
**

**Figure S.1:** Spectra of RP2, **A.** The mass spectra were recorded on a Thermo LTQ-XL spectrometer, **B.** JNM-ECA 500 series NMR Jeol spectrometer (JEOL, Japan) at 500 MHz was used to record ^1^H NMR. **Note:** In some spectra, residual solvent peaks are observed in respective solvents.

**SMJ-1**

1. **
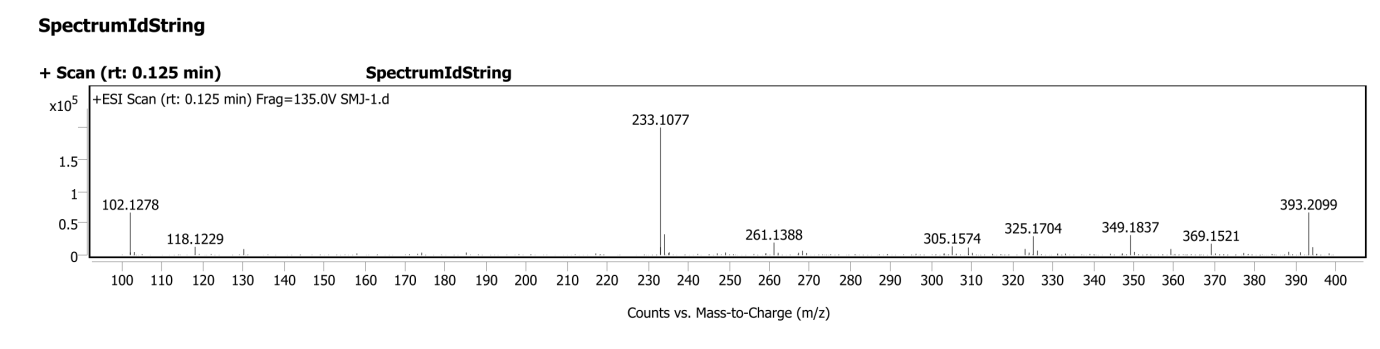

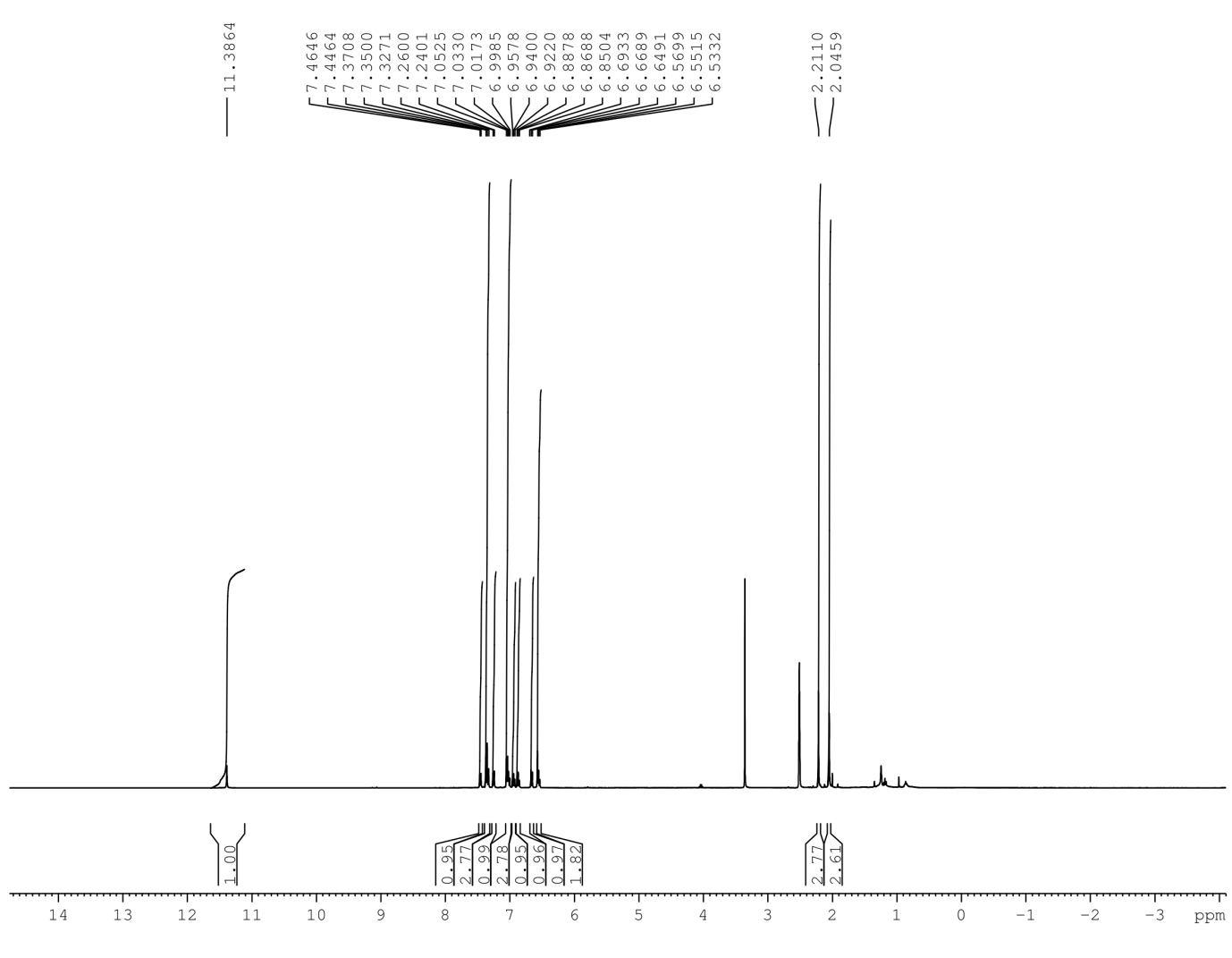
**

**
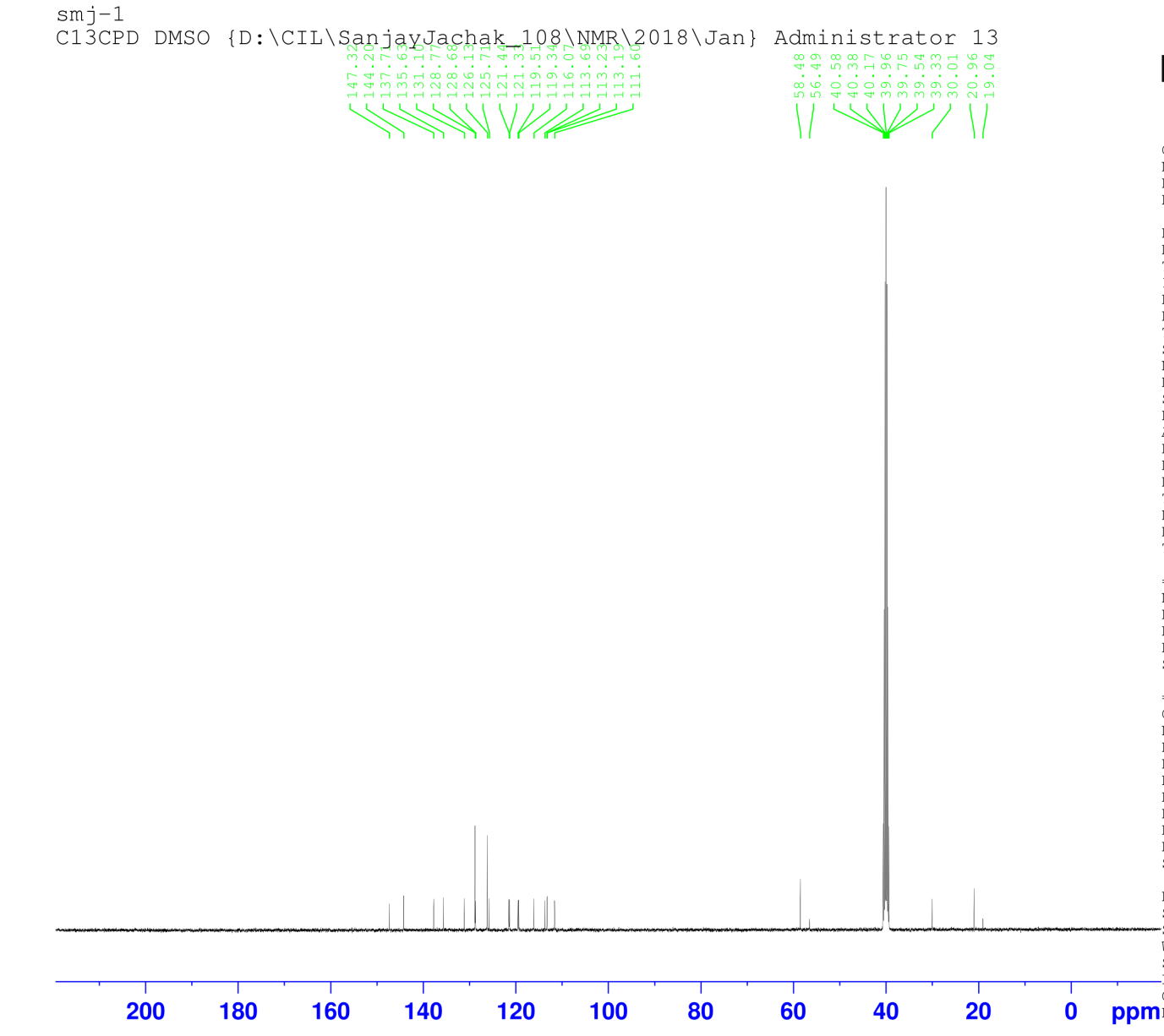
**

**SMJ-3**

**
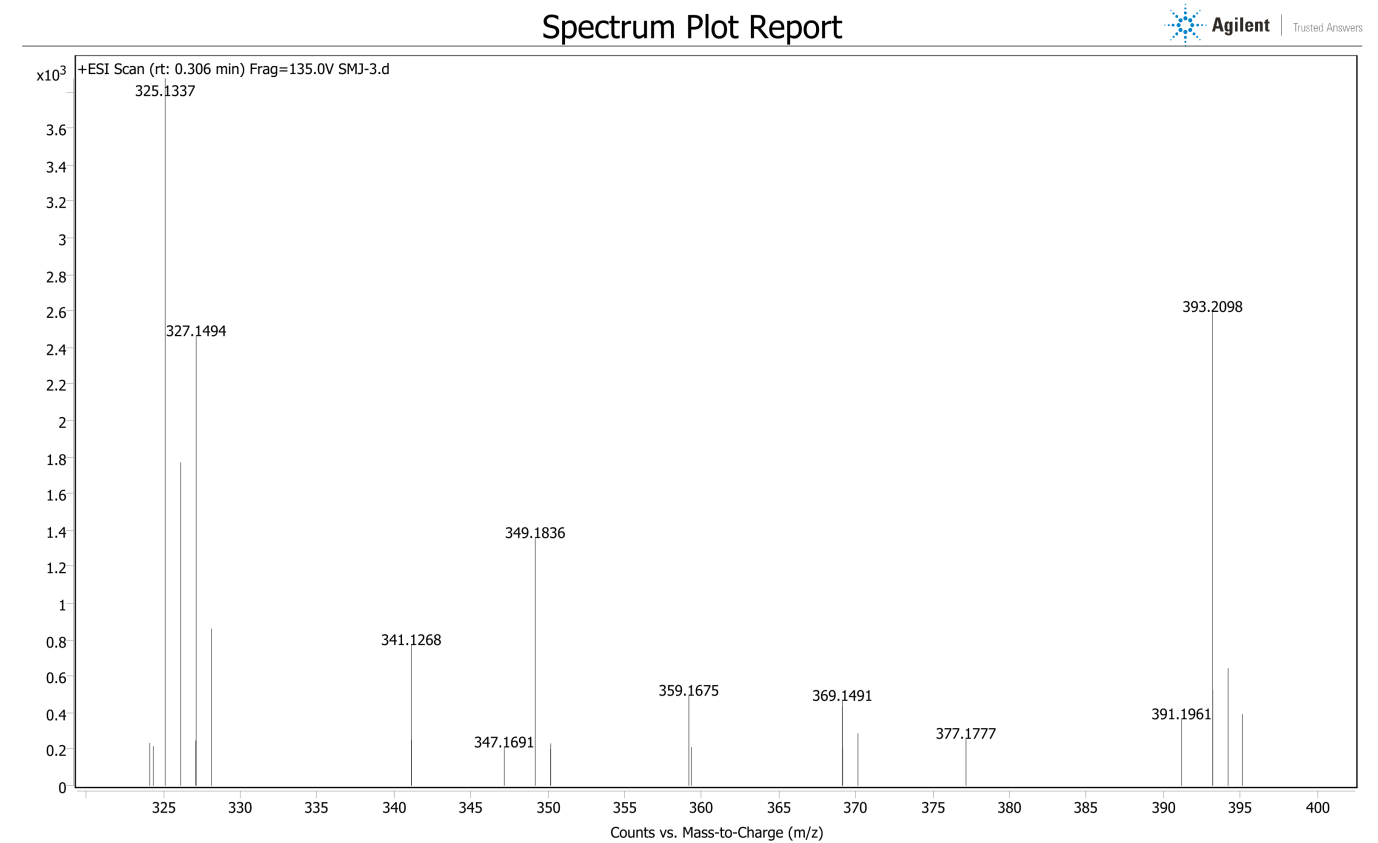
**

**
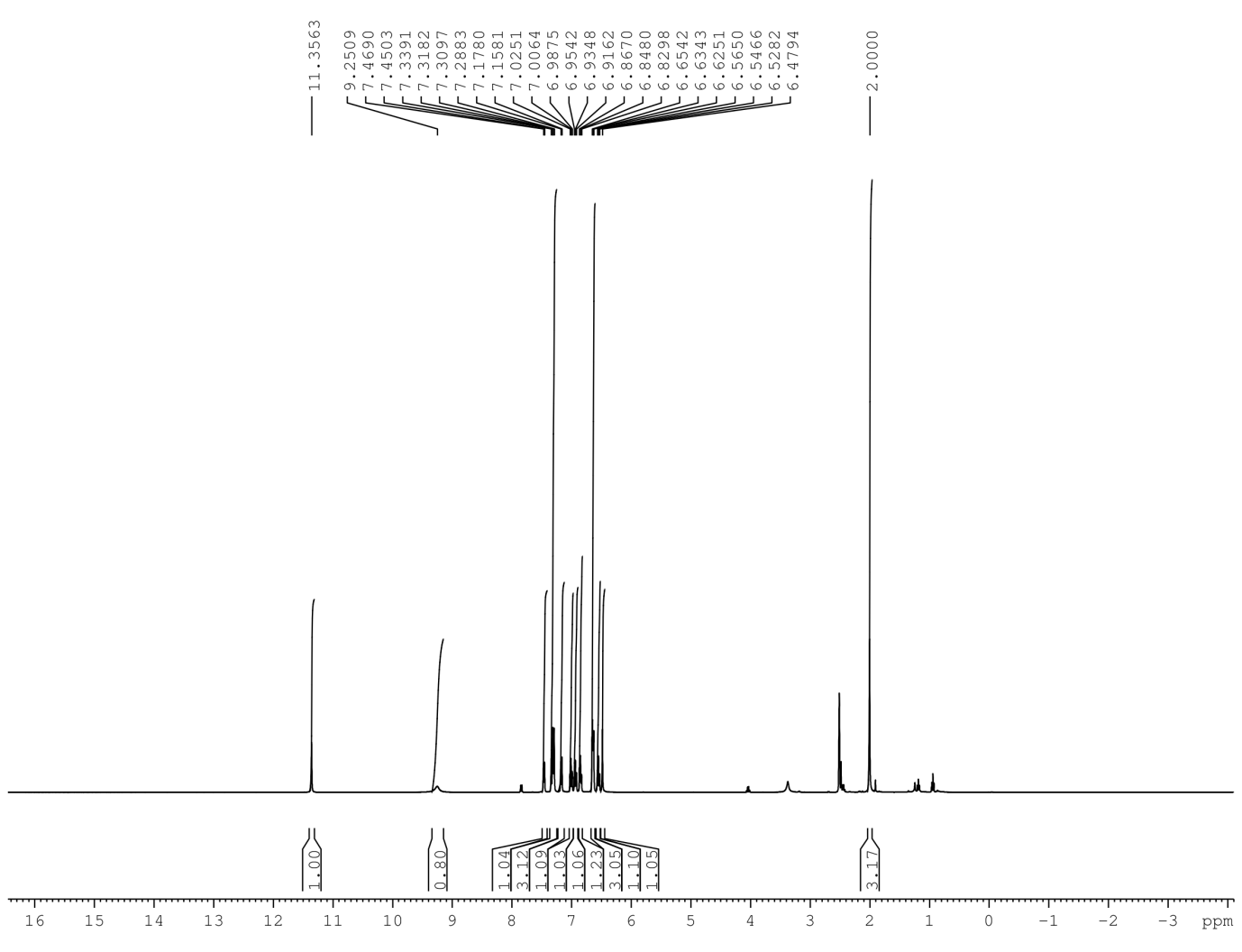
**

**
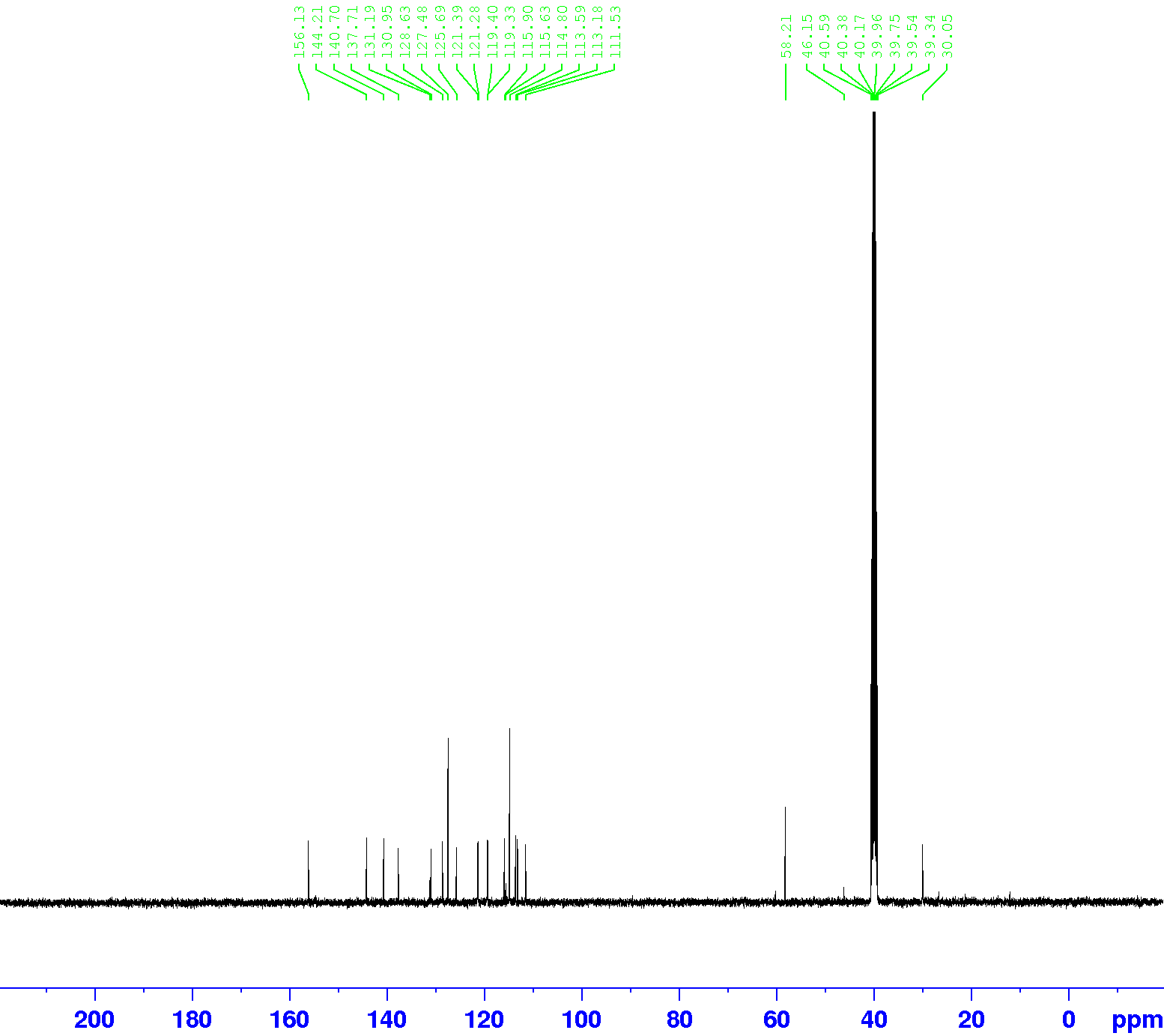
**

**SMJ-5**

**
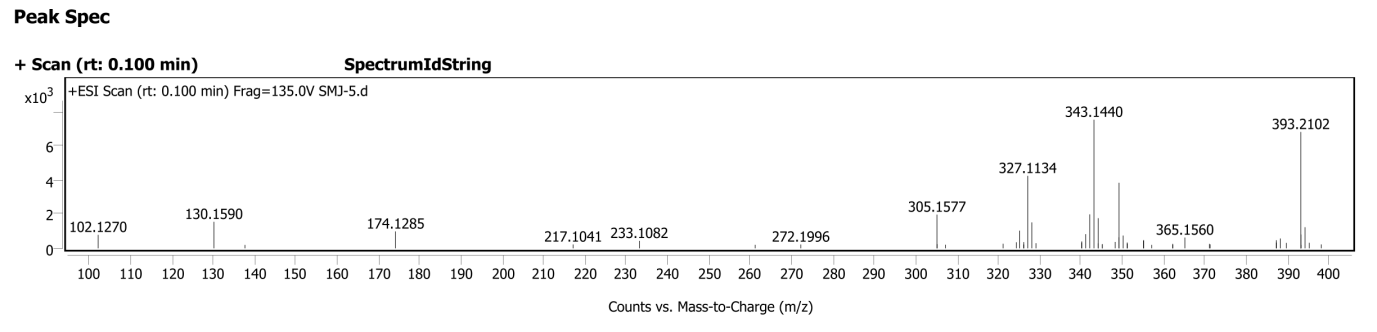
**

**
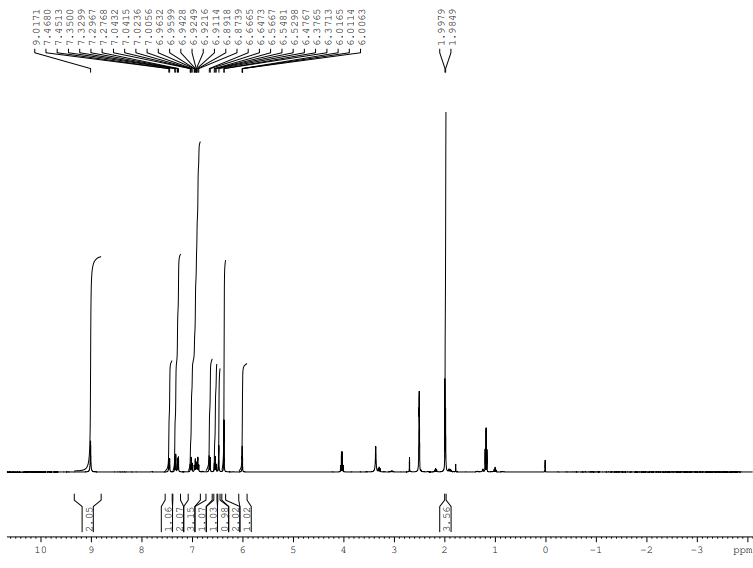
**

**C.**

**
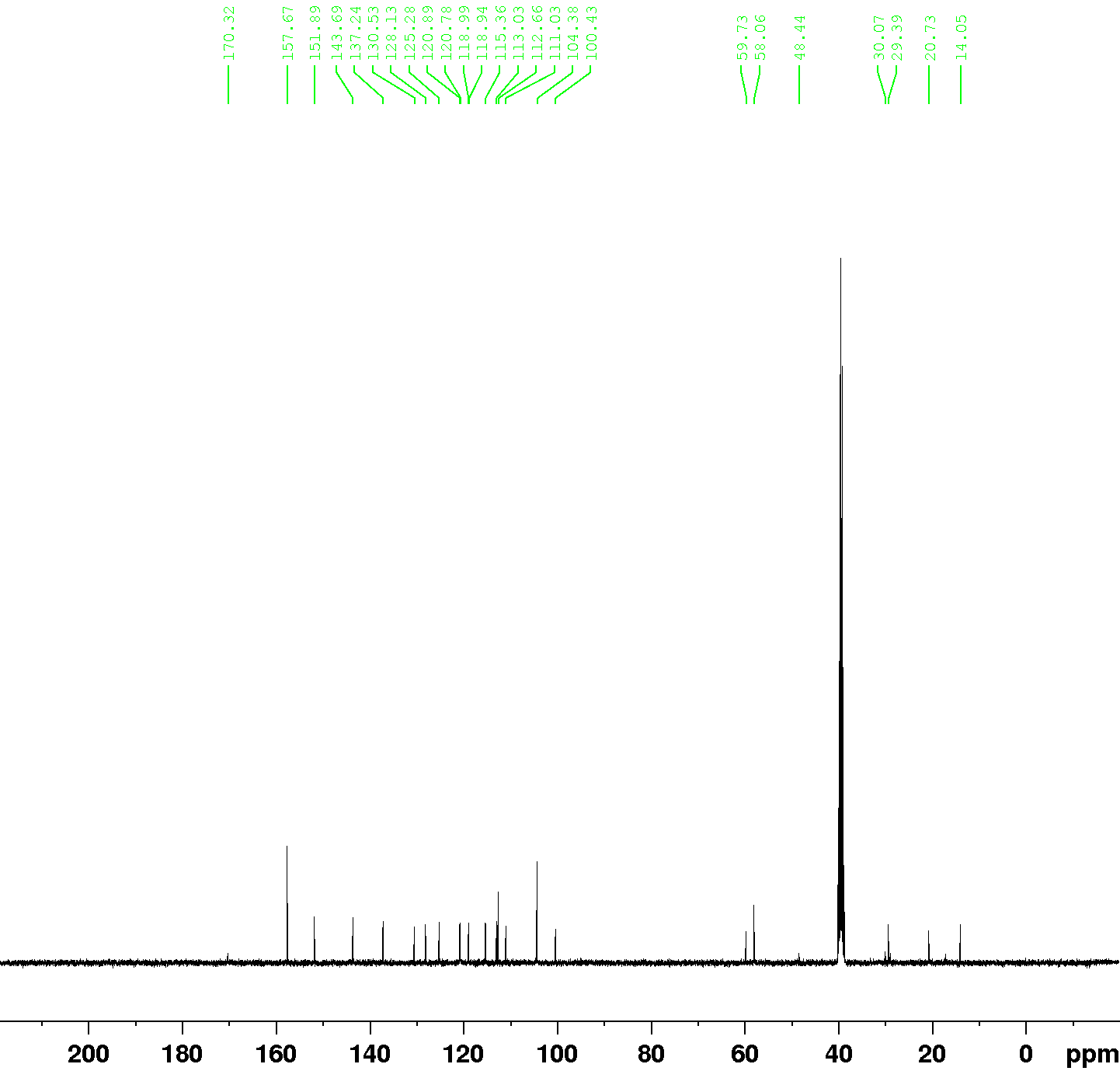
**

**SMJ-6**

**
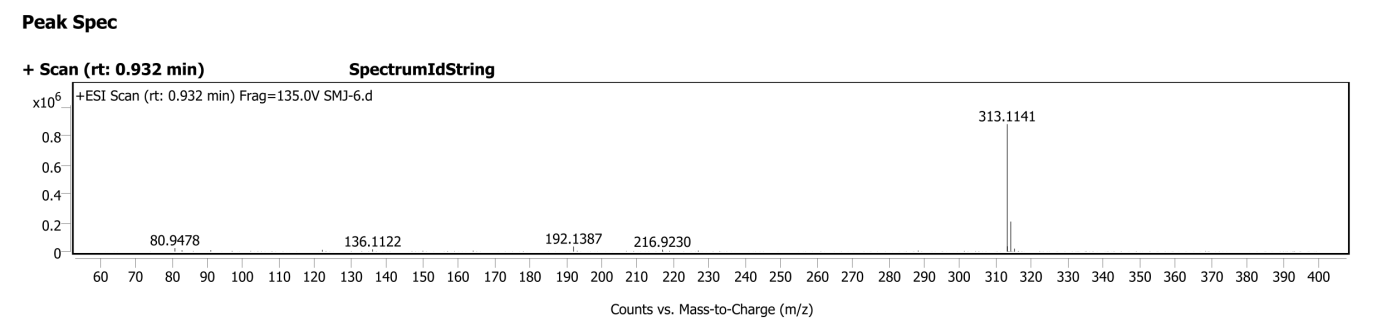
**

**
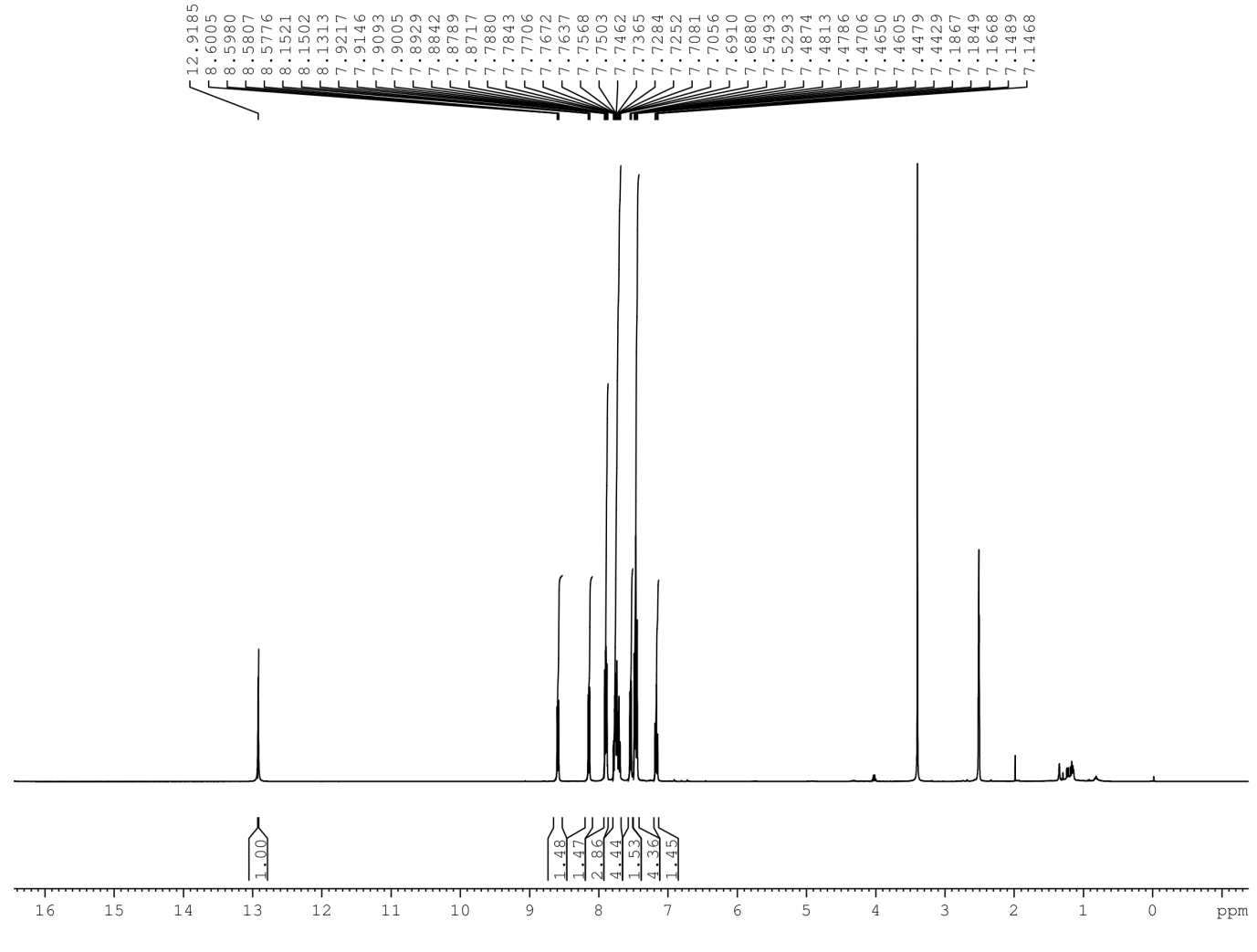
**

**
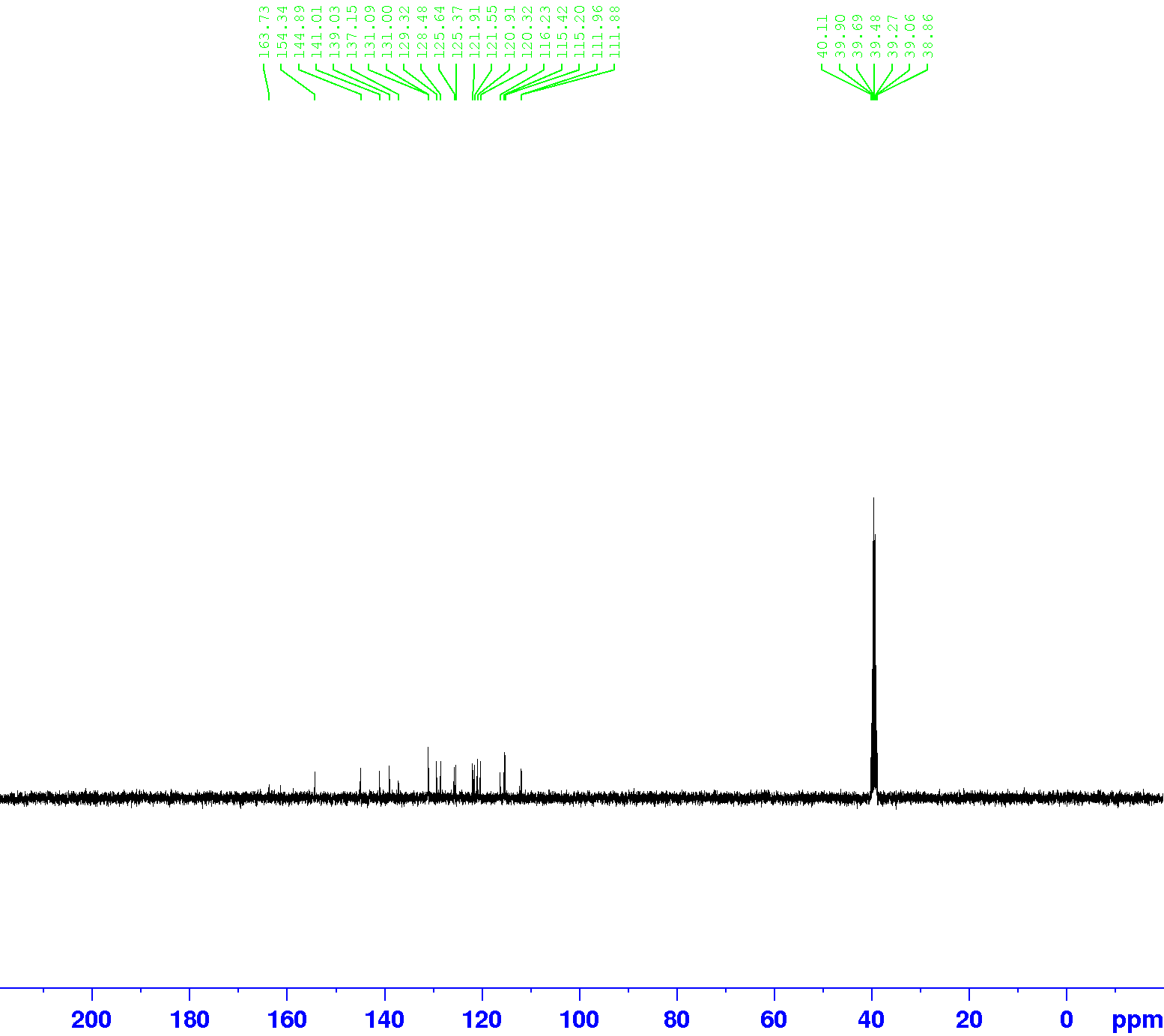
**

**SMJ-7**

**
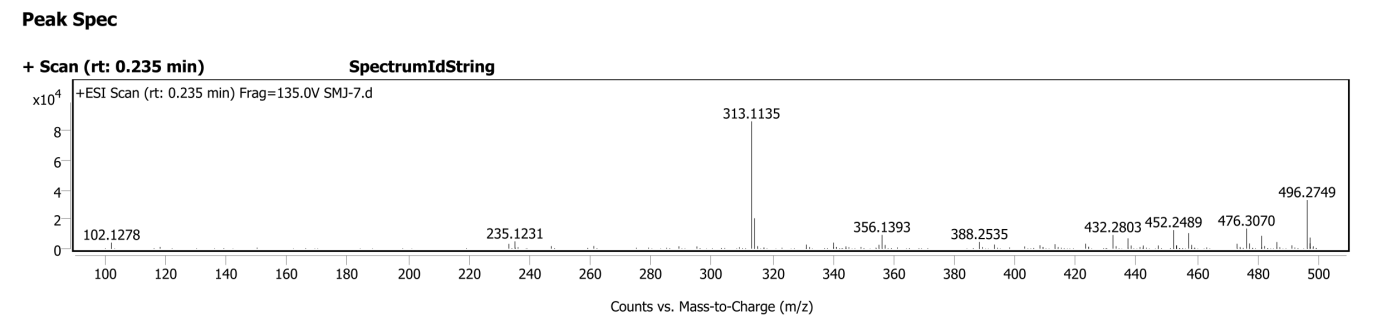
**

**
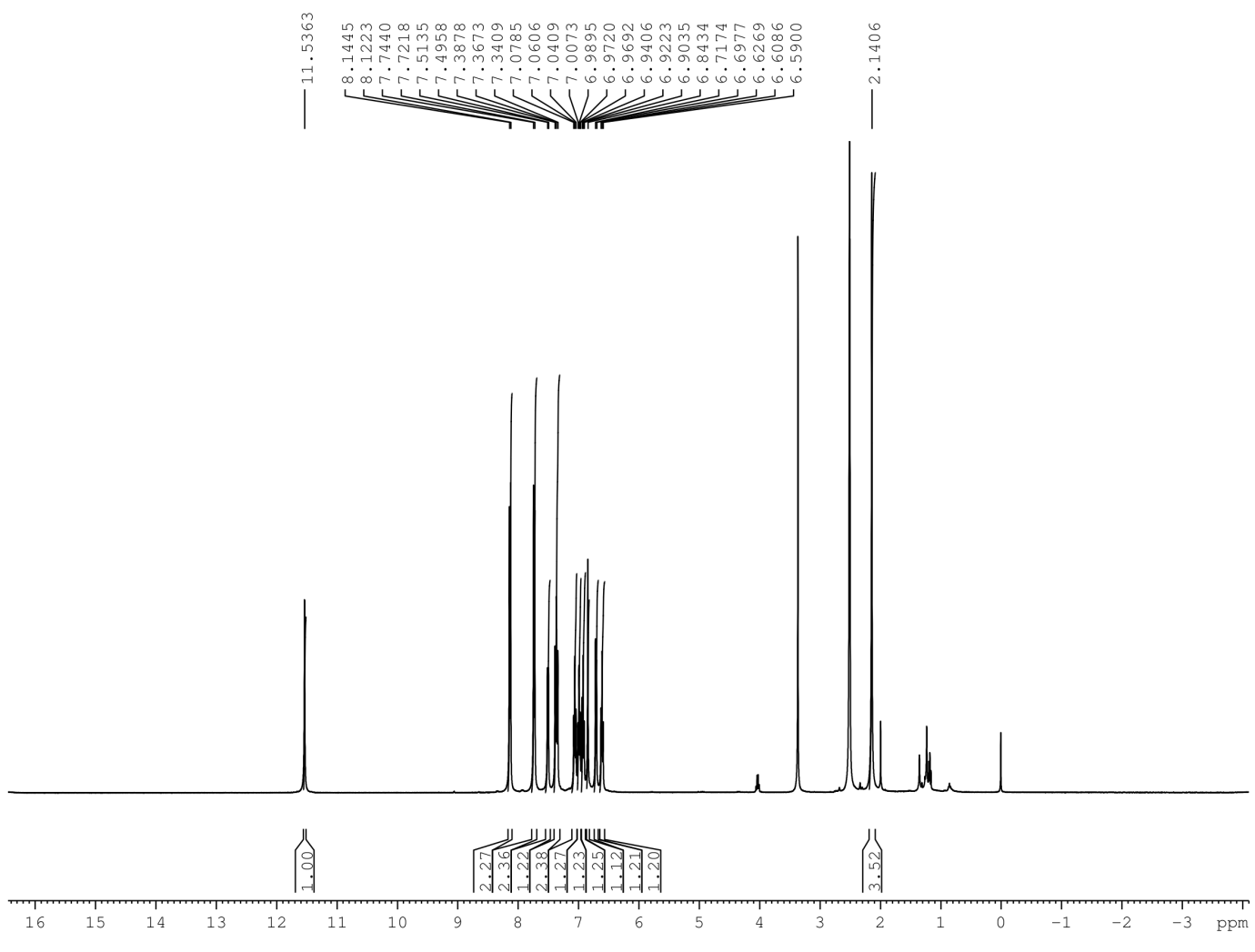
**

**
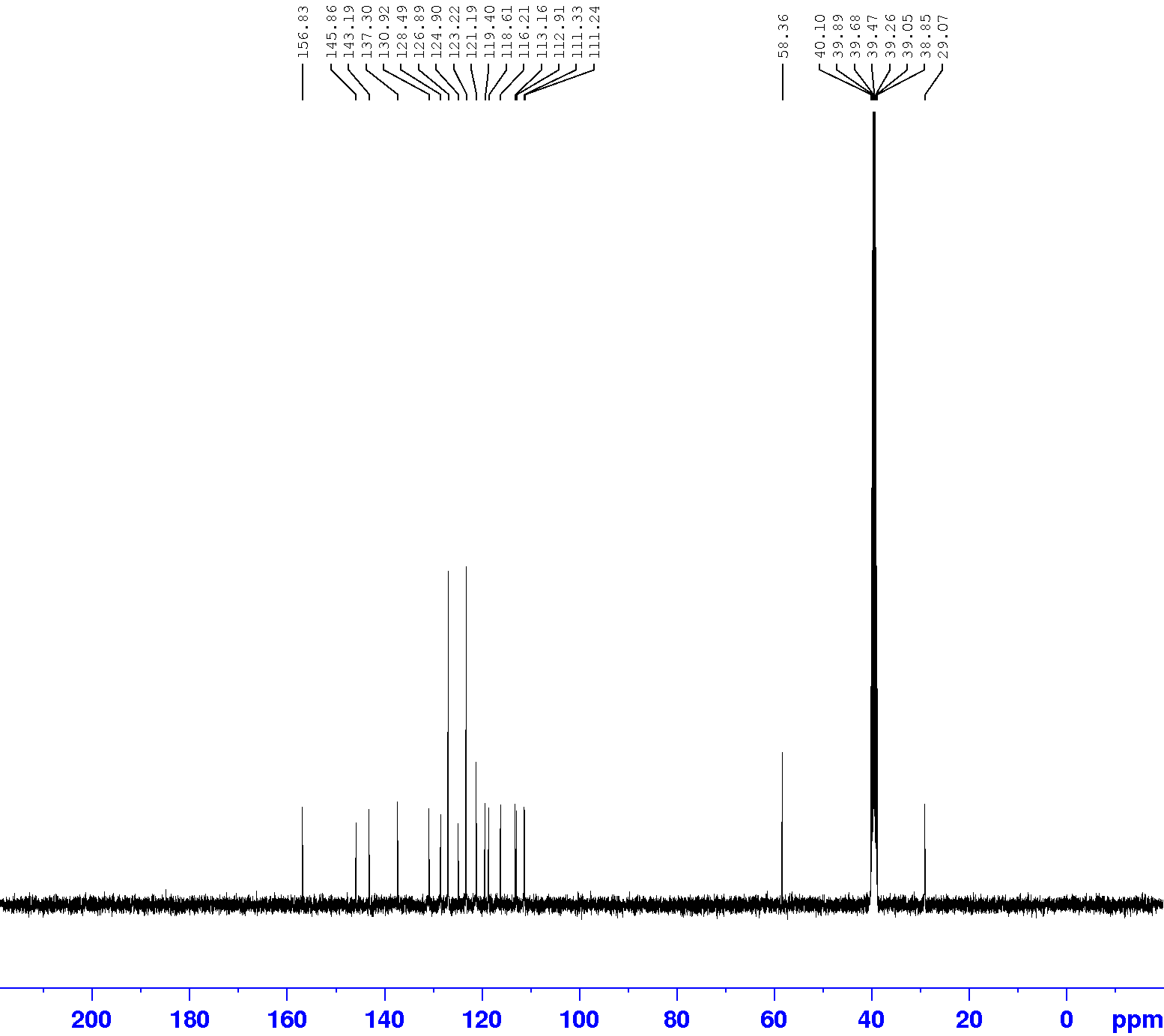
**

**SMJ-8**

**
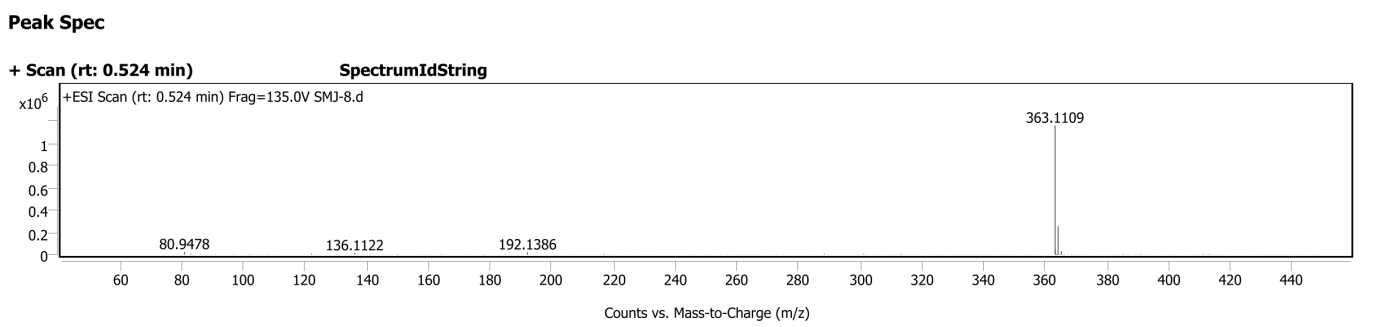
**

**
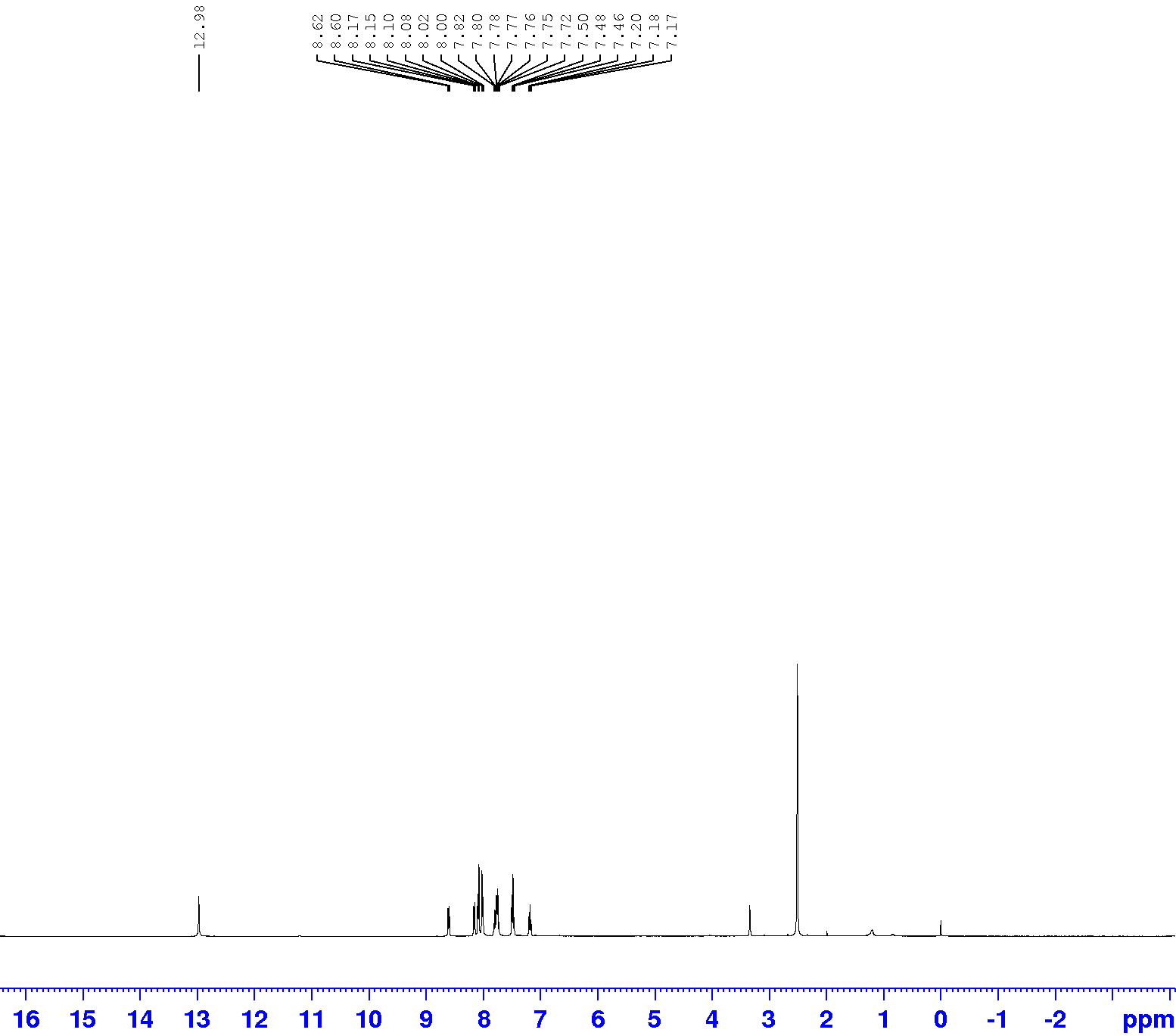
**

**
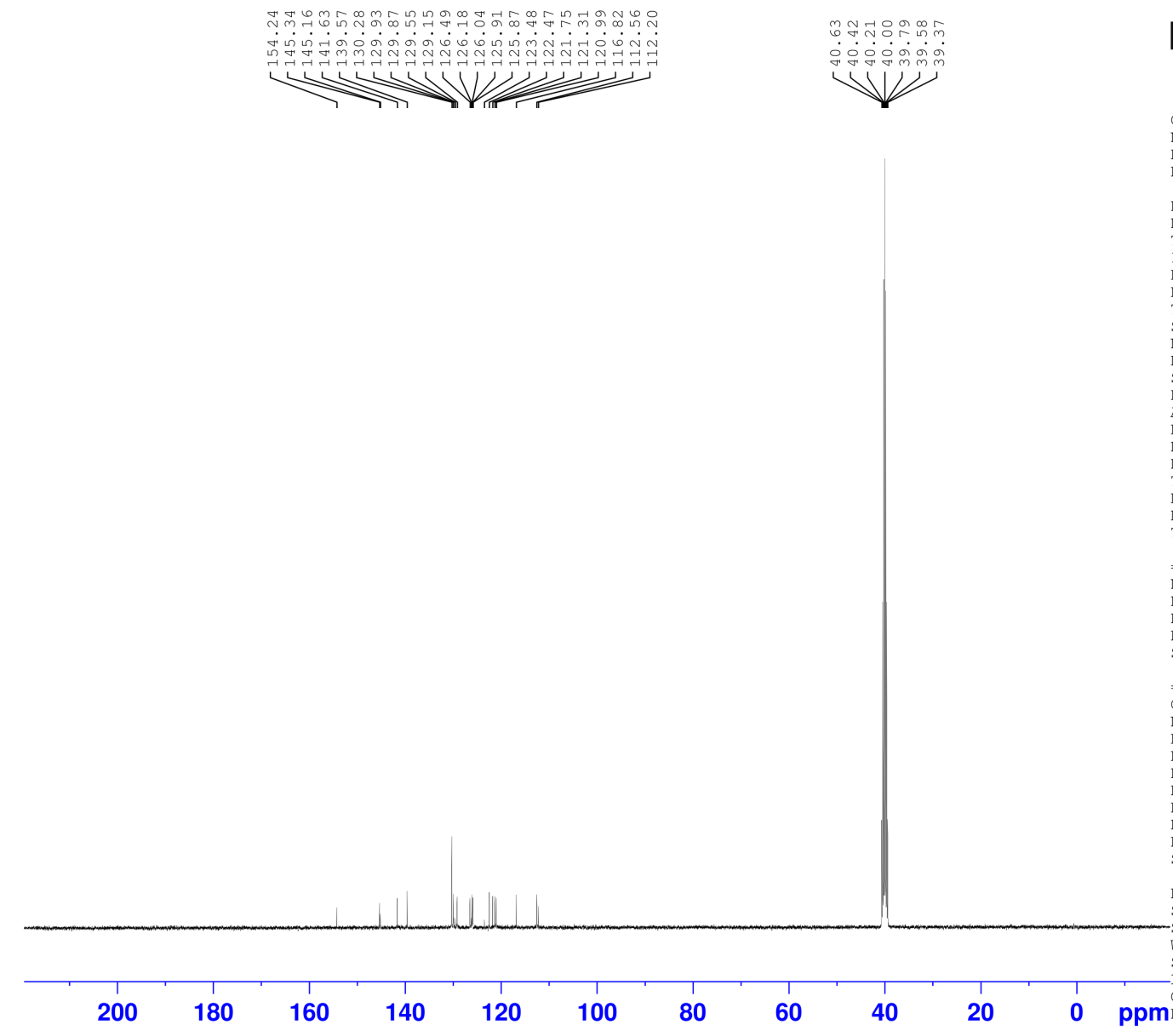
**

**SMJ-9**

**
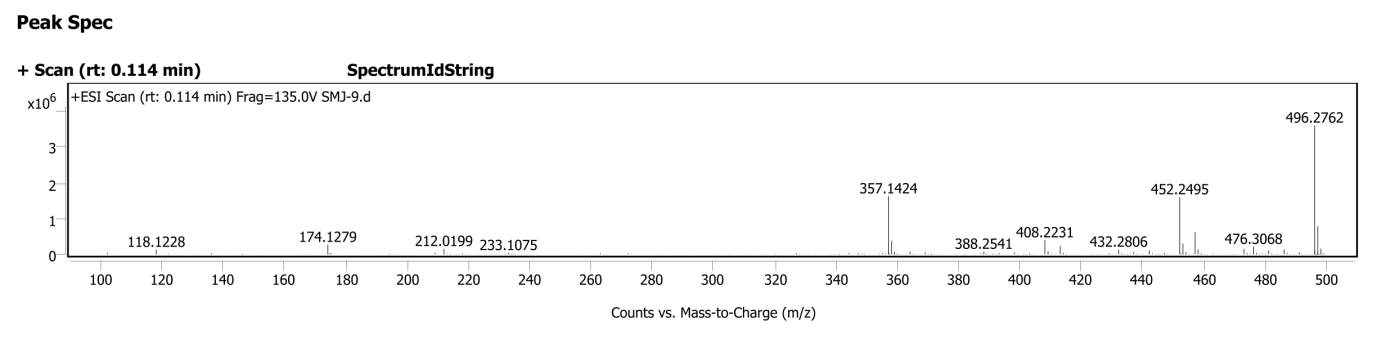
**

**
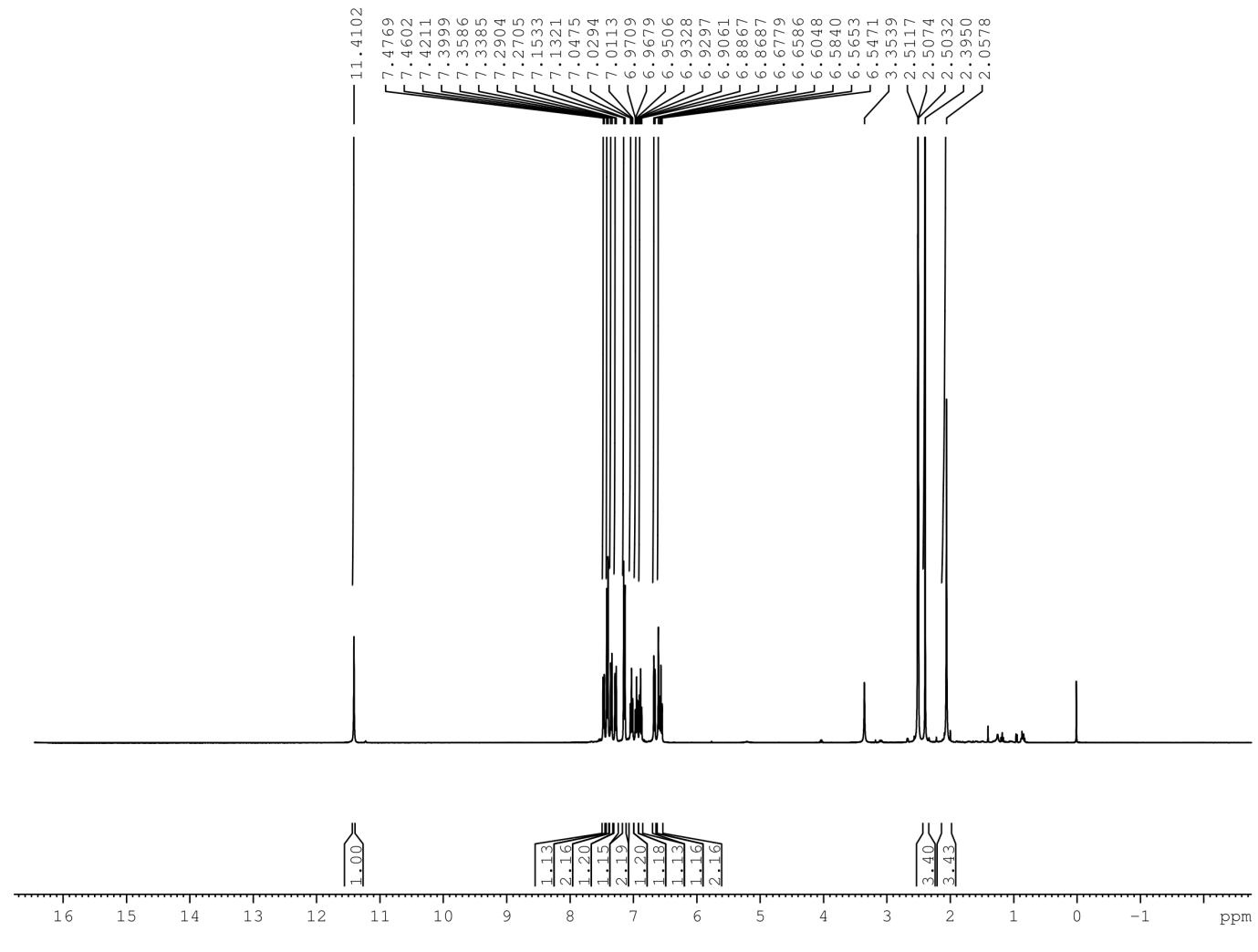
**

**
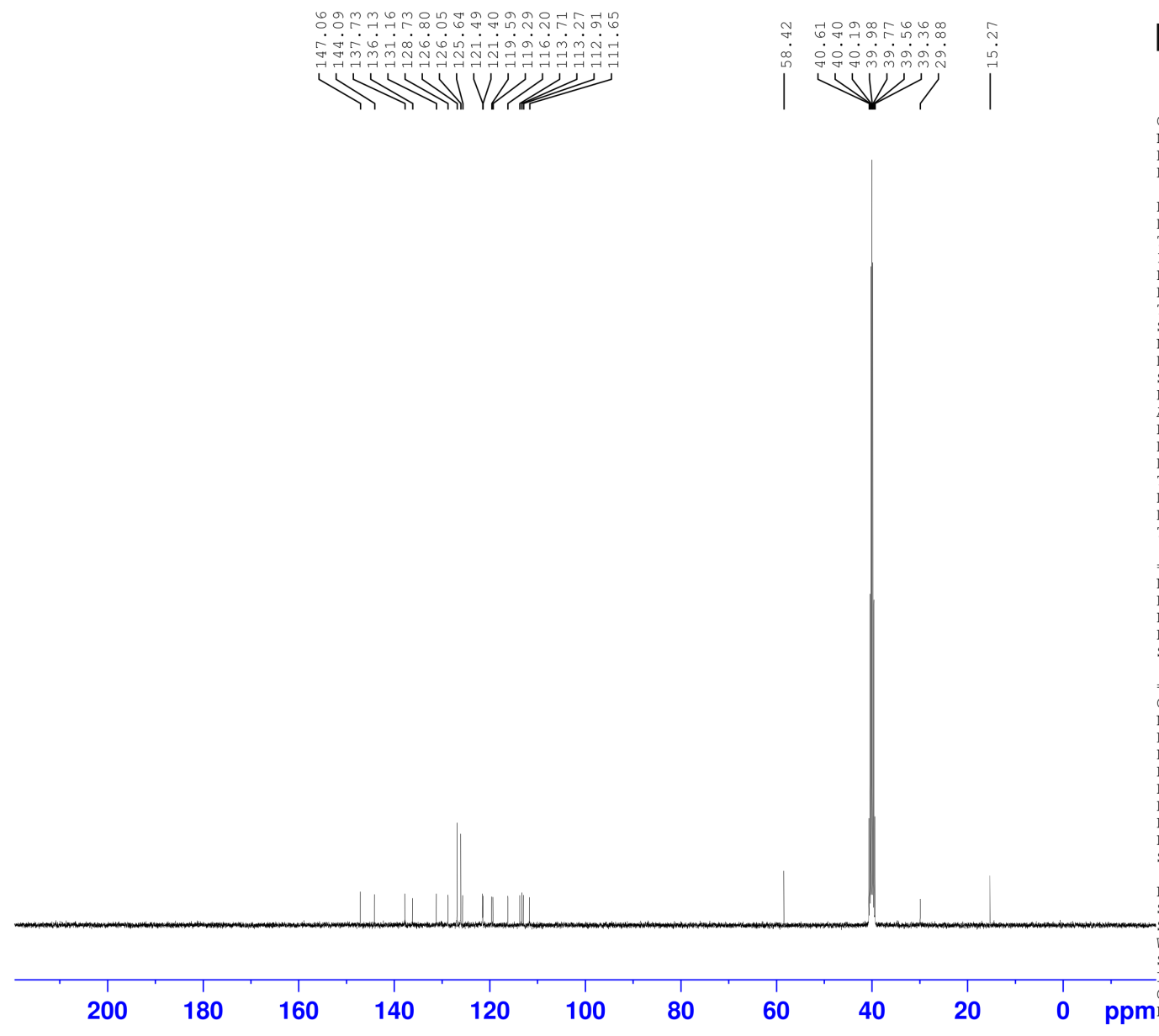
**

**SMJ-10**

**
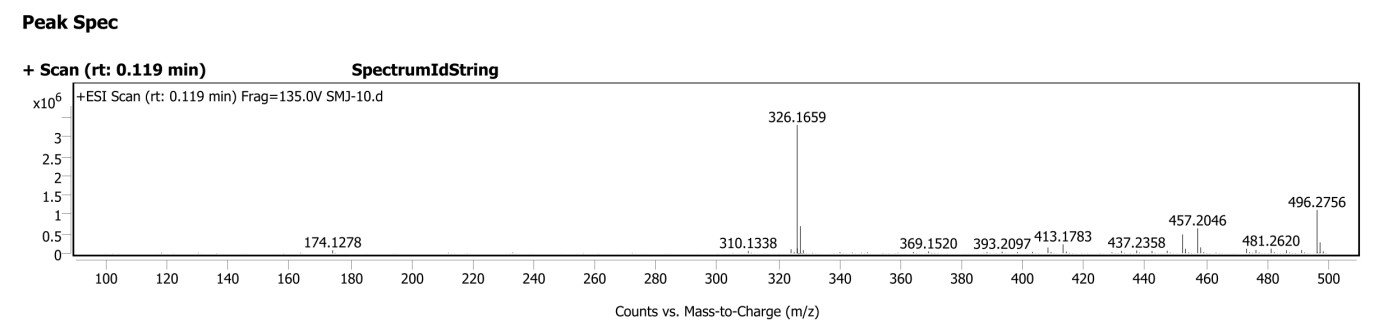
**

**
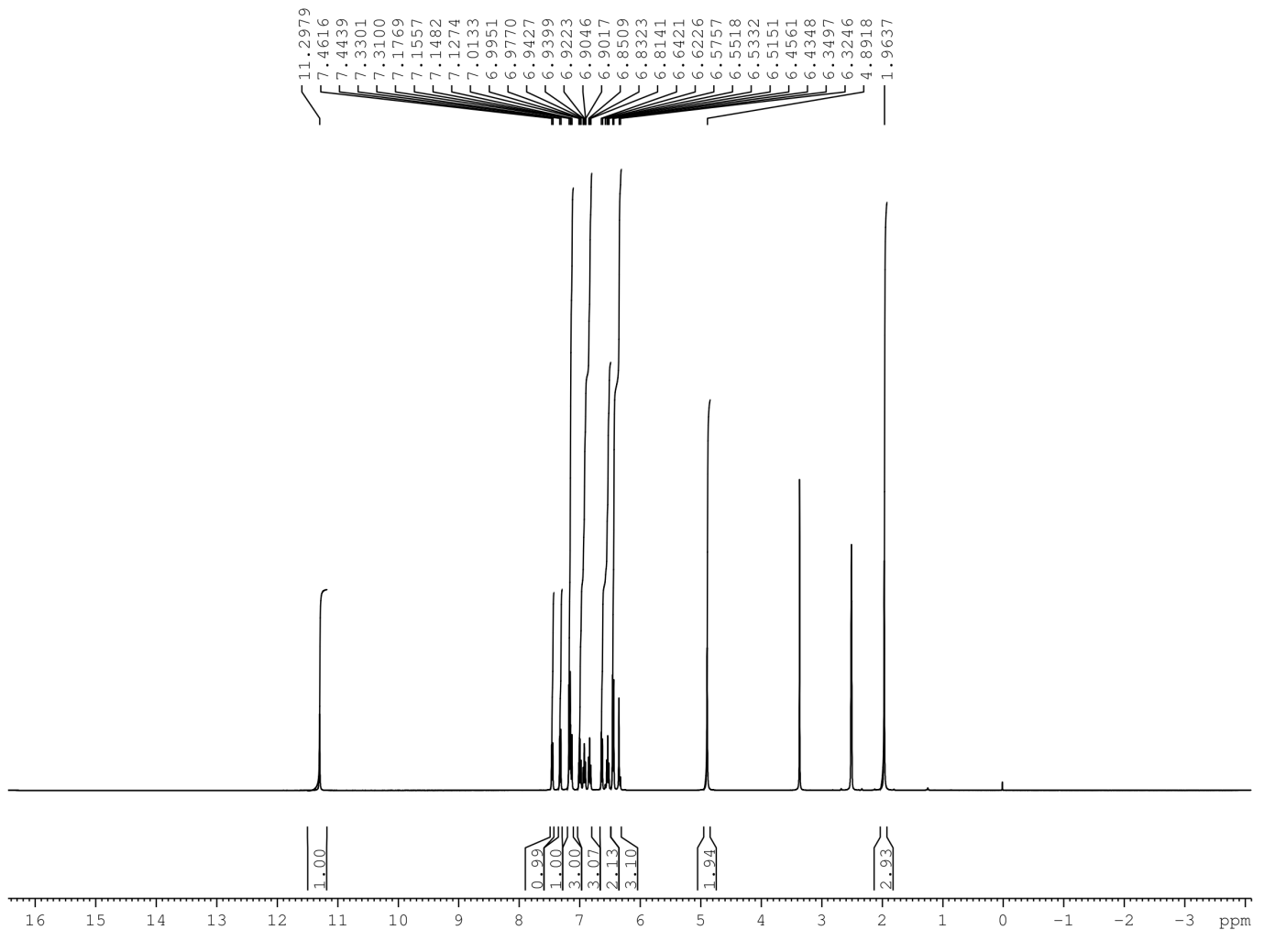
**

**
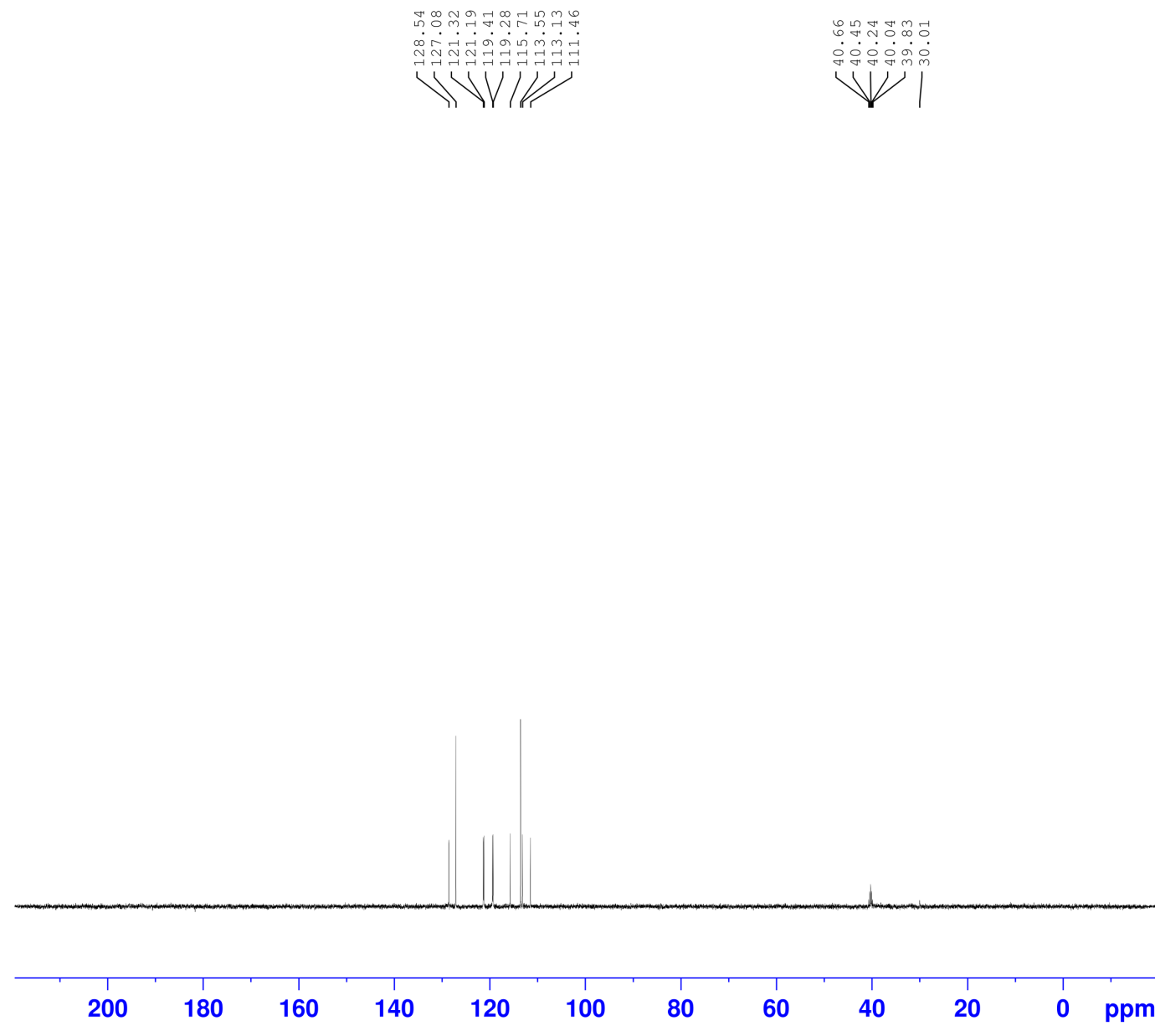
**

**Figure S.2:** Spectra of respective final compounds (SMJ-1 to SMJ-10 namely), **A.** The mass spectra were recorded on Agilent 6200 series TOF/6500 series Q-TOF 10.1 instrument, **B.**^1^H NMR were recorded using BrukerAvance DPX 400 spectrometer (Brukers, Germany) at 400 MHz **C.**^13^C NMR spectra were recorded on BrukerAvance DPX 400 spectrometer (Brukers, Germany) at 100 MHz, respectively, **Note:** In some spectra, residual solvent peaks are observed in respective solvents.


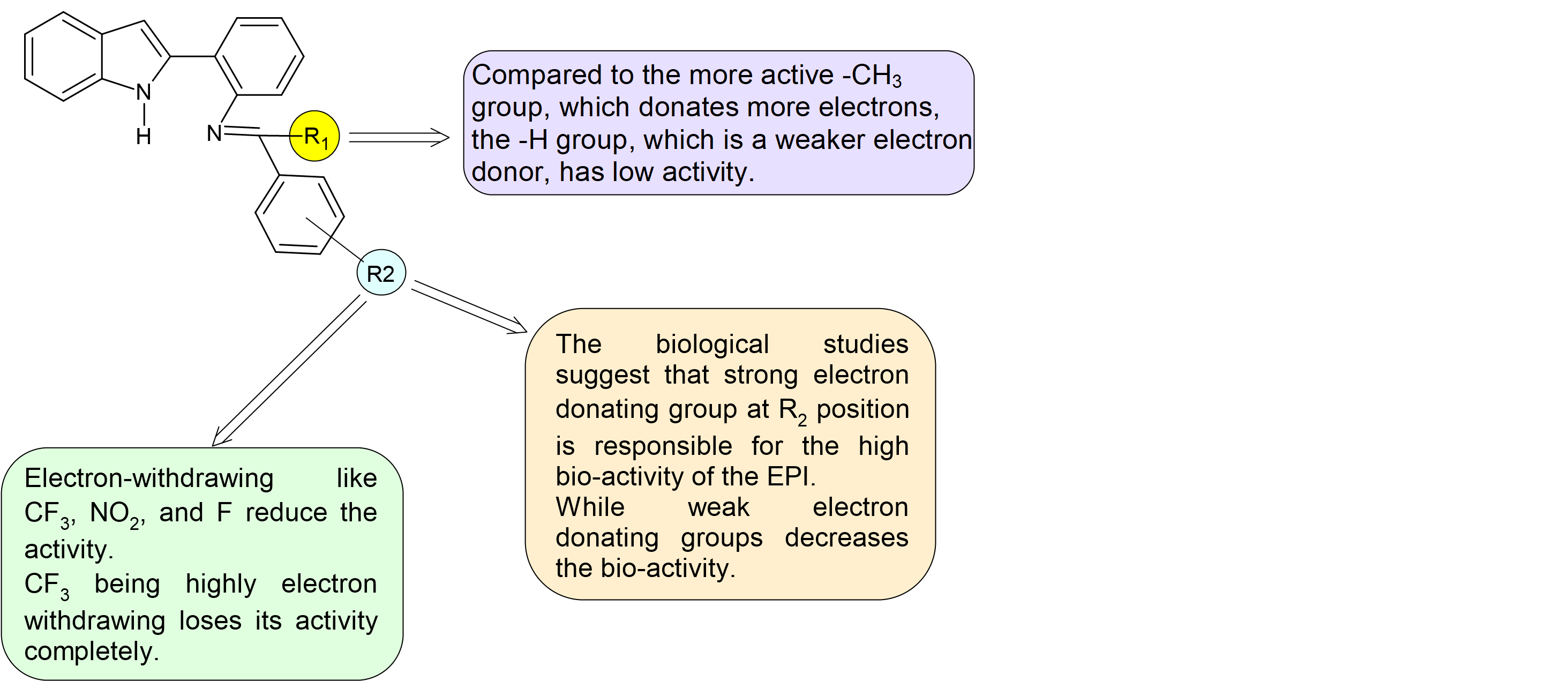


**Figure S.3:** Structure-activity relationship between synthesized derivatives.

**
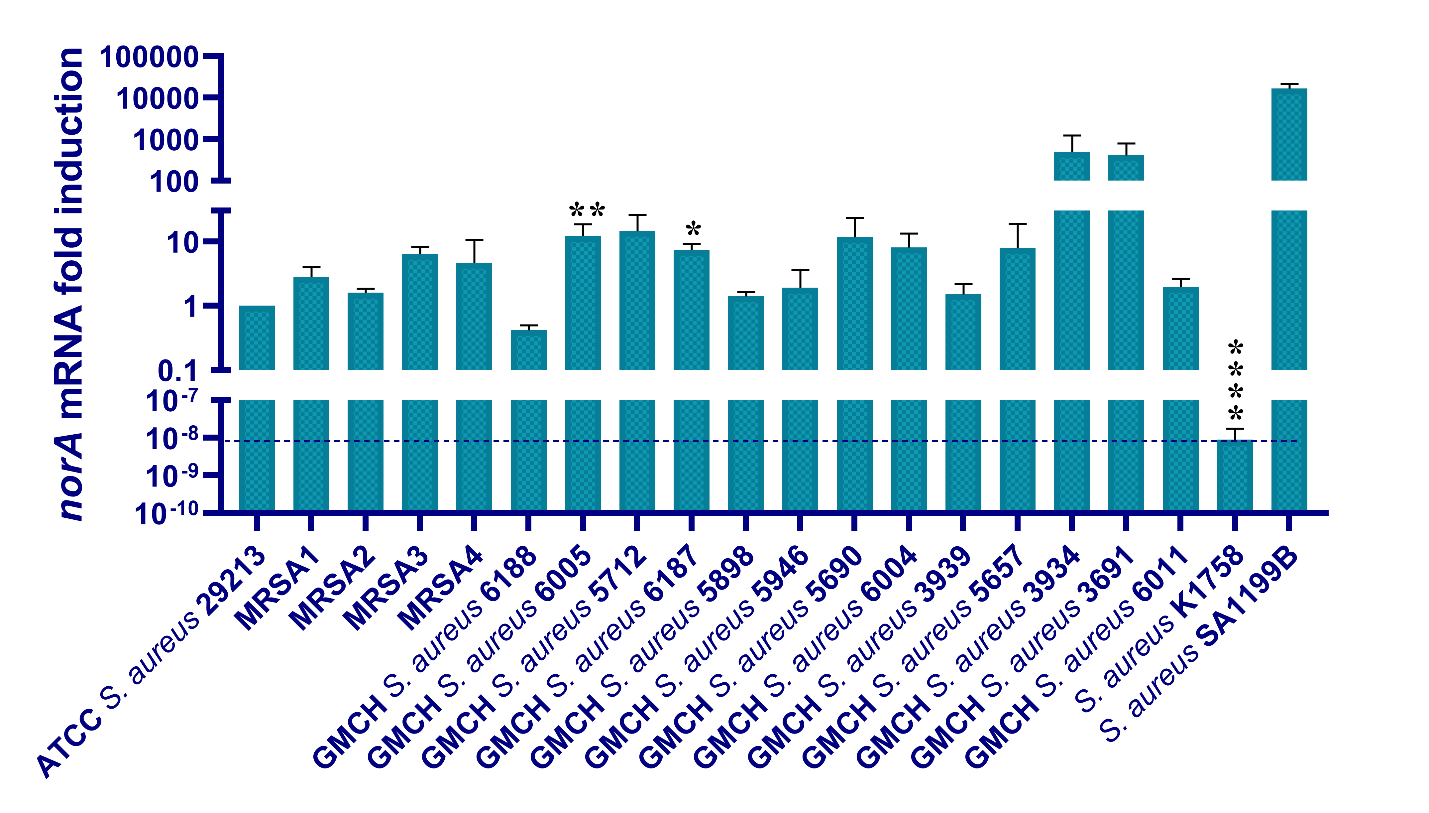
**

**Figure S.4:** Expression analysis of *norA* gene in clinical strains of *S. aureus*. ATCC *S. aureus* 29213 was used as a reference sample strain. Relative gene expression was determined using the power 2^-ΔΔCT^ technique. The housekeeping gene 16s rRNA was used as an endogenous control to normalize the expression levels of target genes. **** represents *P* value <0.0001, calculated using a 95% class interval.

**
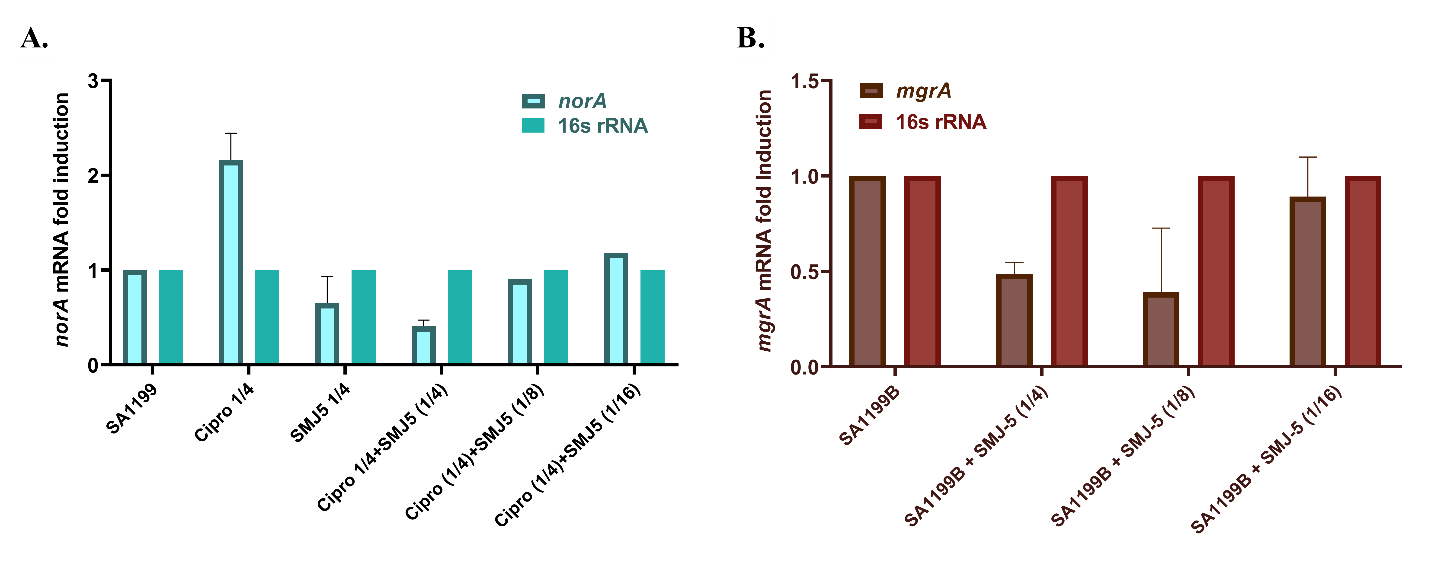
**

**Figure S.5:** **A.** RT-PCR of *norA* gene of *S.aureus* SA-1199 *(norA* wild-type) in untreated, ciprofloxacin (1/4 × MIC), SMJ-5 (1/4 × MIC), and ciprofloxacin (1/32 × MIC) + SMJ-5 (1/4 × MIC), ciprofloxacin (1/32 × MIC) + SMJ-5 (1/8 × MIC) and ciprofloxacin (1/32 × MIC) + SMJ-5 (1/16 × MIC), **B.** RT-PCR of *mgrA* gene of *S.aureus* SA-1199B untreated and SMJ-5 (1/4 × MIC), SMJ-5 (1/8 × MIC), SMJ-5 (1/16 × MIC) treated culture.
